# A general strategy to the intracellular sensing of glycosidases using AIE-based glycoclusters†

**DOI:** 10.1039/d1sc05057e

**Published:** 2021-12-07

**Authors:** Lei Dong, Min-Yu Zhang, Hai-Hao Han, Yi Zang, Guo-Rong Chen, Jia Li, Xiao-Peng He, Sébastien Vidal

**Affiliations:** Key Laboratory for Advanced Materials and Joint International Research Laboratory of Precision Chemistry and Molecular Engineering, Feringa Nobel Prize Scientist Joint Research Center, Frontiers Center for Materiobiology and Dynamic Chemistry, School of Chemistry and Molecular Engineering, East China University of Science and Technology 130 Meilong Rd. Shanghai 200237 P. R. China xphe@ecust.edu.cn; Institut de Chimie et Biochimie Moléculaires et Supramoléculaires, Laboratoire de Chimie Organique 2-Glycochimie, UMR 5246, CNRS, Université Claude Bernard Lyon 1, Université de Lyon 1 Rue Victor Grignard F-69622 Villeurbanne France sebastien.vidal@cnrs.fr; National Centre for Drug Screening, State Key Laboratory of Drug Research, Shanghai Institute of Materia Medica, Chinese Academy of Sciences 189 Guo Shoujing Rd. Shanghai 201203 P. R. China jli@simm.ac.cn; Université Paris-Saclay, CNRS, Institut de Chimie des Substances Naturelles, UPR 2301 91198 Gif-sur-Yvette France

## Abstract

Glycosidases, which are the enzymes responsible for the removal of residual monosaccharides from glycoconjugates, are involved in many different biological and pathological events. The ability to detect sensitively the activity and spatiotemporal distribution of glycosidases in cells will provide useful tools for disease diagnosis. However, the currently developed fluorogenic probes for glycosidases are generally based on the glycosylation of the phenol group of a donor–acceptor type fluorogen. This molecular scaffold has potential drawbacks in terms of substrate scope, sensitivity because of aggregation-caused quenching (ACQ), and the inability for long-term cell tracking. Here, we developed glycoclusters characterized by aggregation-induced emission (AIE) properties as a general platform for the sensing of a variety of glycosidases. To overcome the low chemical reactivity associated with phenol glycosylation, here we developed an AIE-based scaffold, which is composed of tetraphenylethylene conjugated with dicyanomethylene-4*H*-pyran (TPE–DCM) with a red fluorescence emission. Subsequently, a pair of dendritic linkages was introduced to both sides of the fluorophore, to which six copies of monosaccharides (d-glucose, d-galactose or l-fucose) were introduced through azide–alkyne click chemistry. The resulting AIE-active glycoclusters were shown to be capable of (1) fluorogenic sensing of a diverse range of glycosidases including β-d-galactosidase, β-d-glucosidase and α-l-fucosidase through the AIE mechanism, (2) fluorescence imaging of the endogenous glycosidase activities in healthy and cancer cells, and during cell senescence, and (3) glycosidase-activated, long-term imaging of cells. The present study provides a general strategy to the functional, *in situ* imaging of glycosidase activities through the multivalent display of sugar epitopes of interest onto properly designed AIE-active fluorogens.

## Abstract 2

We report a general strategy for the fluorogenic sensing of glycosidases in cells based on aggregation-induced emission of glycoclusters.

## Graphical abstract

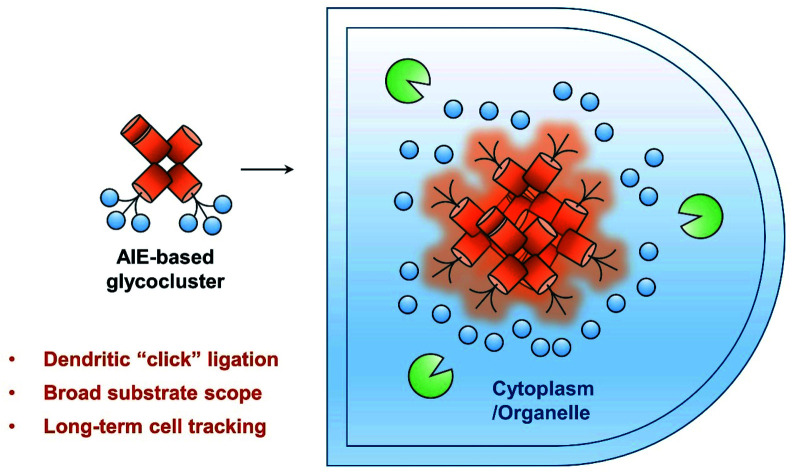

## Introduction

Glycosidases play critical roles in many biological processes through the hydrolysis of monosaccharides from polysaccharides and glycoconjugates (glycolipids or glycoproteins).^1^ Among them, β-d-galactosidase (β-Gal) is an essential enzyme involved in monitoring the efficiency of transcription and transfection of genes, and has also been demonstrated as a key biomarker for cell senescence, ovarian cancer and other pathological processes.^2–4^ α-Fucosidase (AFU) is commonly found in mammalian cells, which hydrolyzes an l-fucosyl residue from glycoconjugates under acidic pH (pH 4 to 6.5). AFU is involved in many biological processes such as inflammation, growth regulation, ligand–receptor recognition, antigenicity, and is also implicated in human diseases including fucosidosis^5^ and carcinoma.^6–8^ Therefore, the ability to accurately monitor the activity of endogenous glycosidases is of prime importance for the basic study of glycobiology as well as for disease diagnosis.

**Fig. 1:**
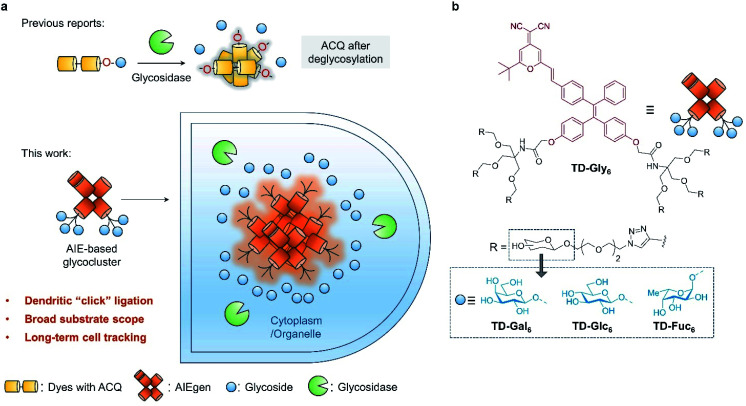
(a) Previously reported glycosidase-activated probes typically with aggregation-caused quenching (ACQ) based on phenol glycosylation, and the development of aggregation-induced emission (AIE)-based glycoclusters (this work) as a general platform for the detection of a wider range of glycosidases, enabling the long-term, glycosidase-activated fluorescence tracking of cells. (b) Structure of the AIE glycoclusters synthesized in this study including AIE-based galactosyl cluster TD-Gal_6_, glucosyl cluster TD-Glc_6_, and fucosyl cluster TD-Fuc_6_.

Compared to traditional colorimetric and enzyme-linked immunosorbent assays, fluorescent probes have been viewed as a more privileged technique for the detection of enzymatic activities due to their high sensitivity, simplicity in design and preparation, and the ability for the *in situ* imaging of enzymatic activities in cells and *in vivo*.^9–14^ The rationale by which glycosidase probes are designed typically relied on the conjugation of one molecule of monosaccharide to the phenol group of a donor–acceptor (D–A) type fluorogen (Table S1†). This glycosylation reaction quenches the intrinsic fluorescence of the phenol dye through modulation of the intramolecular charge transfer (ICT) or photo-induced electronic transfer (PeT) process.^15–18^ Subsequently, hydrolysis of the glycosyl group from the dye by a selective glycosidase causes the fluorescence to recover (fluorescence *off–on*) (Fig. 1a). A number of fluorogenic glycosidase probes has been developed based on this strategy with good sensitivity and selectivity.^18–21^ To minimize background fluorescence and light scattering, the phenol glycosylation strategy has been extended to near-infrared (NIR) dyes for the monitoring of glycosidase activities *in vivo*.^22–28^ However, the previously used dyes for glycosidase detection generally suffer from aggregation-caused quenching (ACQ) in aqueous media,^26,29^ and are easily bound unselectively to biomacromolecules leading to possible false-positive signals.^30–32^

Here, we develop glycocluster-based probes characteristic of aggregation-induced emission (AIE) for the fluorogenic sensing and long-term cell imaging of a diverse range of glycosidases. We have previously developed a D–A-type AIE fluorogen (AIEgen) based on a tetraphenylethylene (TPE) core conjugated with a dicyano-methylene-4*H*-pyran (DCM) derivative (TPE–DCM or TD) with a reddish fluorescence emission (*λ*^em^_max_ = 625 nm).^33,34^ TPE and DCM were conjugated to each other through an ethylene bridge to increase the delocalization of electrons in the system. Unlike previous reports that directly glycosylate phenol groups of conjugated dyes, we introduced a pair of dendritic arms on both phenol moieties of TPE (Fig. 1a). This enables the grafting of six copies of glycosyl epitopes pre-functionalized with an azido-triethyleneglycol to the fluorophore through azide–alkyne click chemistry (Fig. 1b). By comparison to a number of other synthetic glycoclusters bearing different linkages between the glycoside and the AIEgen, the dendritic strategy has proven to be the most effective yielding a sensitive fluorogenic response to glycosidases. The AIE-based glycoclusters were successfully used for (1) the fluorogenic detection of functionally diverse glycosidases including galactosidase, glucosidase and fucosidase, (2) the fluorescence imaging of the endogenous glycosidase activities in healthy and cancer cells, and during cell senescence, and (3) glycosidase-activated, long-term imaging of healthy and cancer cells.

## Results and discussion

### Synthesis and structure–activity relationship of TPE–DCM-based glycoclusters

**Scheme 1:**
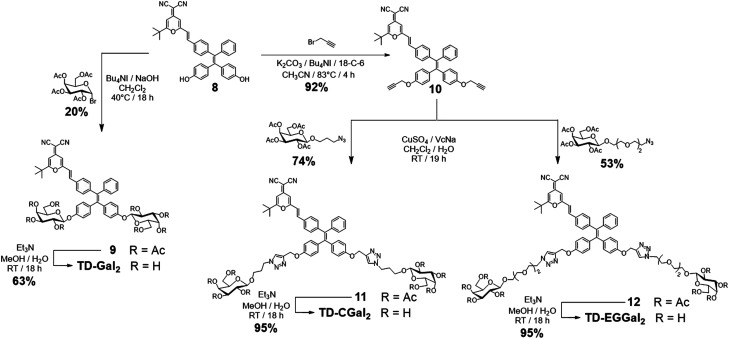
Synthesis of TD-Gal_2_, TD-CGal_2_ and TD-EGGal_2_.

**Scheme 2:**
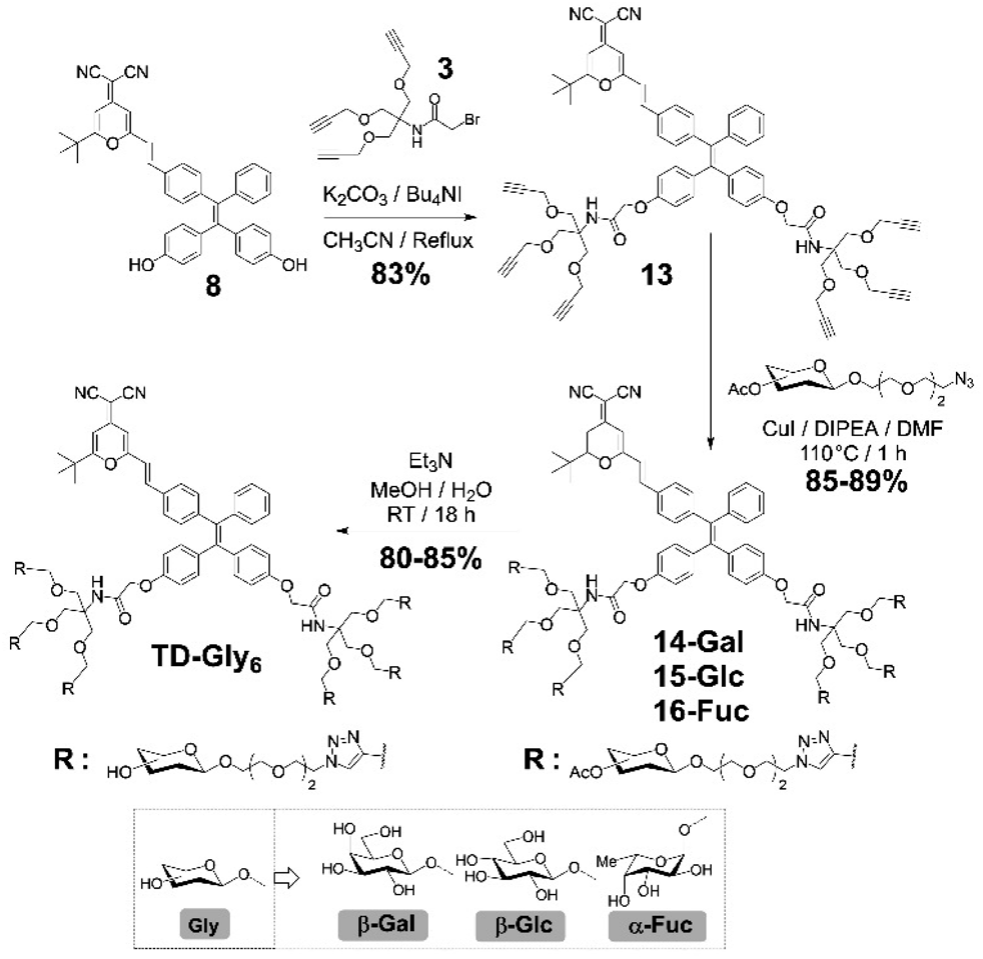
Synthesis of the TD-Gal_6_, TD-Glc_6_ and TD-Fuc_6_.

Four series of TPD–DCM-based glycoclusters with different linking patterns between TPE–DCM and monosaccharides were designed and synthesized; d-galactose was preliminarily used as a model for the study of structure–activity relationship.^35–37^ The galactosyl groups were introduced to the AIEgen by direct phenol glycosylation (linker free) (TD-Gal_2_) or azide–alkyne click chemistry conjugation through structurally different linker arms (short-arm, propyl: TD-CGal_2_; long-arm, triethyleneglycol: TD-EGGal_2_; dendritic-arm: TD-Gal_6_) (Schemes 1 and 2).

The linker-free glycocluster TD-Gal_2_ was synthesized from intermediate 8. The known TPE-aldehyde 5^35^ was coupled to DCM 6^38^ through aldolization/crotonization. The resultant conjugate 7 with a red fluorescence emission showed a gradual fluorescence enhancement with increasing water fraction in THF, which is characteristic of AIE (Fig. S1†).^39^ Demethylation of 7 with BBr_3_ in CH_2_Cl_2_ afforded the bis-phenolic 8 (Scheme S1†). Subsequently, glycosylation under phase transfer conditions with bromo tetra-*O*-acetyl-d-galactose afforded the desired bis-glycosylated derivative 9. As expected, this bis-phenol glycosylation led to a poor yield of 20%; the stereoselectivity of the glycosylation was verified by analysis of the ^1^H NMR spectrum of the compound, suggesting a β-anomeric configuration (*J*_1,2_ = 8.2 Hz). Solvolysis of the acetyl protecting groups afforded the desired glycocluster TD-Gal_2_ (Scheme 1).

With TD-Gal_2_ in hand, we tested its fluorescence response to β-Gal (purified from *E. coli*)^40^ in a phosphate buffered saline (PBS) at pH 7.4 (Fig. S2a†). The fluorescence of the probe was observed to slightly decrease in the first 10 min, and then sharply increase from 10 to 40 min, in the presence of β-Gal. However, we determined that the fluorescence of TD-Gal_2_ in the absence of the enzyme also increased with time in PBS (pH 7.4). This suggests that (1) the initial fluorescence emission intensity of the probe in PBS was unstable probably due to a slow rate of molecular aggregation, and, thus exhibited a time-dependent AIE-based fluorescence enhancement, and (2) the two galactosyl residues modified on the phenol groups did not sufficiently quench the fluorescence of the AIEgen.

With the traditional phenol glycosylation strategy being unsatisfactory, we then sought to enhance the intrinsic AIE property of TPE–DCM through improving the water solubility of the glycoclusters. The better water solubility of the glycoclusters might lead to a lowered initial fluorescence emission of the system. When the glycosyl groups are removed from the dye, the decreased water solubility of TPE–DCM could lead to a drastically enhanced emission through AIE (Fig. 1a). To achieve this goal, additional linker arms (propyl, triethyleneglycol or dendritic) were introduced to TPE–DCM between the phenolic AIEgen and the galactosyl groups. The linker arms are retained on the dye scaffold even after hydrolysis of the glycosyl groups, thereby facilitating the formation of TPE–DCM-based amphiphilic micelles enhancing the AIE fluorescence.

The Cu(i)-catalyzed azide–alkyne cycloaddition (CuAAC) “click” chemistry was used for conjugation between the dye and monosaccharides.^41–43^ Starting from compound 8, propargylation was first attempted using potassium carbonate with propargyl bromide, which led to a poor chemical yield (<15%). Addition of Bu_4_NI and 18-crown-6 enhanced the reactivity of phenolates for a proper etherification in 92% yield to obtain the bis-propargyl compound 10 (Scheme 1). Galactosides with a 3-azidopropyl^44^ (short-arm) or azido-triethyleneglycol^45^ (long-arm) aglycon were then conjugated to compound 10 in the presence of CuSO_4_/sodium ascorbate (VcNa) as catalyst.^33,46^ Solvolysis of the ester protecting groups afforded the TD-CGal_2_ and TD-EGGal_2_ probes from compound 11 and 12, respectively (Scheme 1). The fluorescence of TD-CGal_2_ and TD-EGGal_2_ was then measured without β-Gal in PBS buffer at pH 7.4 (Fig. S2b and c†). Both glycoclusters showed a strong fluorescence emission, suggesting that the amphiphilic molecules are partially aggregated before reacting with the enzyme. Upon addition of the enzyme, the fluorescence of both probes decreased slightly. This might be the result of the formation of a poorly water-soluble AIE species after the enzymatic hydrolysis. These results suggest that the conjugation of only two glycosides onto the AIEgen is not sufficient to substantially enhance the water solubility of the dye.

We then sought the possibility of introducing more than two glycosyl groups to the phenolic positions of the AIEgen in order to substantially enhance the water solubility, achieving an optimal “*off–on*” fluorescence response upon incubation with glycosidases. Consequently, a trivalent dendron was used to incorporate three glycosyl residues with each phenol group of TPE–DCM. Tris(hydroxymethyl)aminomethane (Tris) was converted into the tris-propargylated precursor 3 by a sequential synthetic route including Boc-protection, propargylation, removal of the Boc-carbamate and 2-bromo-acetamidation (Scheme 2). 2-Bromo-acetamide 3 was then conjugated to the TPE–DCM bis-phenol 8 to afford the hexakis-propargylated compound 13. CuAAC coupling of 13 with azido-triethyleneglycol galactoside was achieved under microwave activation to maximize the reactivity and completeness of the reaction.^47,48^ Removal of the protecting groups afforded the water-soluble hexavalent glycocluster TD-Gal_6_. The same synthetic strategy was also applied to the synthesis of the hexavalent glucosylated probe TD-Glc_6_ and fucosylated probe TD-Fuc_6_ (Scheme 2).

Subsequently, a comparative study of the AIE properties of TD-EGGal_2_, TD-Gal_6_, TD-Glc_6_ and TD-Fuc_6_ (10 μM) in a mixture solvent system of THF/PBS at different ratios (pH 7.4) was carried out (Fig. S3†). TD-EGGal_2_ displayed a typical fluorescence enhancement upon increase of PBS ratio in the solvent mixture (Fig. S3a†). This observation suggests that the presence of only two glycosides is not sufficient for TD-EGGal_2_ to remain well dispersible in water. In contrast, the fluorescence intensity of the hexakis-glycosylated probe TD-Gal_6_ in THF decreased with PBS (Fig. S3b†). The well quenched fluorescence of the probe in full PBS suggests that TD-Gal_6_ was hardly aggregated in aqueous solution. Other hexakis-glycosylated probes, TD-Glc_6_ (Fig. S3c†) and TD-Fuc_6_ (Fig. S3d†), showed a similar trend of fluorescence quenching with PBS ratio being increased. We noticed a slight fluorescence recovery of TD-Glc_6_ and TD-Fuc_6_ in 100% PBS, which we ascribe to a slightly different aggregation manner of the compounds in this fully aqueous environment. However, when the concentration of the probes was gradually decreased from 10 to 2.5 μM, the fluorescence enhancement became neglectable (Fig. S4†).

### Glycosidase sensing with the AIE-based hexavalent glycoclusters

With the TPE–DCM-based hexavalent glycoclusters (TD-Gly_6_) in hand, we then tested their fluorescence responses to glycosidases in PBS. TD-Gal_6_ with six galactosyl epitopes was first used for the detection of β-Gal purified from bacteria. The β-Gal isolated from *Escherichia coli* (β-Gal/*E. coli*) has been extensively used for analysis of the sensing performances of fluorogenic β-Gal probes.^28^ However, the working pH of this enzyme is restrained to 6.5–7.5.^40^ Alternatively, Gao *et al.* reported the use of β-Gal purified from *Aspergillus oryzae* (β-Gal/*A. oryzae*) with a more acidic working pH range of 4.0–5.0, which is more similar to that of lysosomes.^49,50^ As a result, we attempted the detection of β-Gals isolated from different origins (bacterium or fungus) in order to examine the sensitivity of the AIE-based glycoclusters developed.

**Fig. 2:**
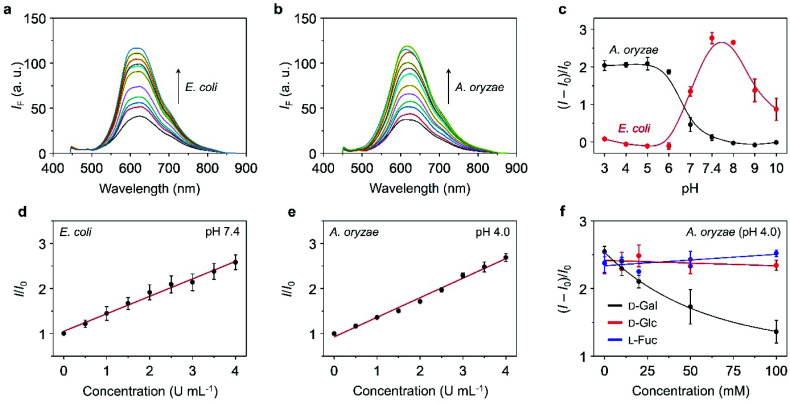
Fluorescence emission spectra of TD-Gal_6_ (10 μM) with (a) increasing β-Gal (0–7 U mL^−1^) purified from *E. coli* at pH 7.4, and (b) β-Gal (0–7 U mL^−1^) purified from *A. oryzae* at pH 4.0. (c) Plotting the fluorescence changes of TD-Gal_6_ (10 μM) in the presence of β-Gal (5 U mL^−1^) purified from *E. coli* and that (5 U mL^−1^) purified from *A. oryzae* as a function of pH. Plotting the fluorescence changes of TD-Gal_6_ (10 μM) as a function of (d) increasing β-Gal (0–4 U mL^−1^; interval: 0.5 U mL^−1^) purified from *E. coli* at pH 7.4, and (e) increasing β-Gal (0–4 U mL^−1^; interval: 0.5 U mL^−1^) purified from *A. oryzae* at pH 4.0. (f) Plotting the fluorescence changes of TD-Gal_6_ (10 μM) with β-Gal (5 U mL^−1^) purified from *A. oryzae* at pH 4.0 as a function of increasing free d-Gal, d-Glc and l-Fuc. *I* and *I*_0_ are the fluorescence intensity of TD-Gal_6_ in the presence and absence of an analyte, respectively. All fluorescence spectra were recorded in PBS (0.01 M, pH as indicated) with an excitation at 420 nm.

TD-Gal_6_ was incubated separately with β-Gal/*E. coli* (pH 7.4) (Fig. 2a) and β-Gal/*A. oryzae* (pH 4.0) (Fig. 2b). We observed a concentration-dependent fluorescence enhancement of the probe for both galactosidases at their optimal pH. A good linearity was observed by plotting the fluorescence enhancement of TD-Gal_6_ as a function of the concentration (0–4.0 U mL^−1^) of β-Gal/*E. coli* (Fig. 2d) and β-Gal/*A. oryzae* (Fig. 2e) at pH 7.4 and 4.0, respectively. The limit of detection of the probe for β-Gal/*E. coli* and β-Gal/*A. oryzae* was determined to be 0.015 U mL^−1^ and 0.014 U mL^−1^, respectively. Comparing to previously reported fluorogenic probe, TD-Gal_6_ is among the most sensitive for β-Gal, and its unique structural feature of possessing multiple glycosyl epitopes endows the glycocluster with a better water solubility and potentially good biocompatibility for biological assays (Table S1†). In addition, we also determined that the AIE-based fluorescence enhancement is relevant to the specific working pH range of both enzymes (Fig. 2c). Within an acidic pH range (3–6), TD-Gal_6_ responded stably to β-Gal/*A. oryzae*, whereas the fluorescence of the probe only enhanced at a relatively neutral-basic pH (7–9) for β-Gal/*E. coli*. This suggests that the AIE-based glycocluster probe can differentiate the working pH of homogeneous glycosidases derived from different source (bacterium or fungus).

Next, the sensing properties of TD-Gal_6_ was tested in more detail. Owing to the sufficient water-solubility due to the presence of six galactosyl groups, the fluorescence of TD-Gal_6_ remained stable in PBS (pH 4.0 or 7.4) during 60 min of incubation (Fig. S5†). In the presence of β-Gal/*E. coli*, the fluorescence (*Φ*_F_ = 0.005) of TD-Gal_6_ enhanced by *ca.* 5-fold (*Φ*_F_ = 0.026) at pH 7.4 (Fig. S6†). Similarly, a drastic fluorescence enhancement of *ca.* 4.5-fold (*Φ*_F_ from 0.006 to 0.027) was observed for the glycocluster with β-Gal/*A. oryzae* at pH 4.0 (Fig. S7†). This suggests that the cleavage of the glycosidic bonds in the probe by the enzyme reduced the hydrophilicity of the fluorescent dye, thereby leading to molecular aggregation. This action allowed for the detection of β-Gal through AIE effect in full PBS solution without the need for any organic solvent being added.

To better understand the sensing mechanism of TD-Gal_6_ for β-Gal, dynamic light scattering (DLS) and transmission-electron microscopy (TEM) were used to determine the hydrodynamic size and morphology of the molecular aggregates of TD-Gal_6_ in PBS solution before and after treatment with β-Gal, respectively (Fig. S8a and b†). The hydrodynamic diameter of the probe alone was determined to be around 10 nm in PBS buffer. In addition, the solution of TD-Gal_6_ displayed a negligible Tyndall effect, similar to that of control (distilled water) (Fig. S8c†). This observation agrees with the probe being well-dispersed in aqueous solution. After addition of β-Gal/*E. coli* at pH 7.4, the galactosyl groups on the probe were hydrolyzed to decrease the water-solubility of the dye, thereby producing more aggregated species with a particle size of 162 nm (Fig. S8b†). A bright light path was also observed, which we ascribed to the Tyndall effect, corroborating the aggregation with an enhanced fluorescence emission of the residues after enzyme hydrolysis (Fig. S8c†). In their representative TEM images, we also observed a morphological change of TD-Gal_6_ from amorphous to particle-like after being hydrolyzed by β-Gal (Fig. S8,† inset). The mass peak of the enzymatically hydrolyzed residue of TD-Gal_6_ with hexakis hydroxyl moieties was detected by mass spectrometry. Molecular ions of the residual products with one or two galactosides remaining on the dye scaffold could also be detected by mass spectrometry and are probably present in traces amounts (Fig. S9†). Moreover, a competition assay was also carried out. Pre-treatment of β-Gal/*A. oryzae* (pH 4.0) (Fig. 2f) and β-Gal/*E. coli* (pH 7.4) (Fig. S10a†) with free d-galactose (as β-Gal inhibitor) led to the concentration-dependent fluorescence suppression of TD-Gal_6_. In contrast, the pre-treatment of d-glucose and l-fucose did not cause the fluorescence of the probe to decrease in the presence of β-Gal/*A. oryzae* or β-Gal/*E. coli*. These data suggest that the enzymatic sensing was achieved by TD-Gal_6_ being selectively hydrolyzed by a galactosidase, leading to molecular aggregation with an enhanced fluorescence.

Next, we tested the fluorescence response of other hexavalent glycoclusters to their corresponding glycosidase. We observed a significant fluorescence enhancement of TD-Glc_6_ and TD-Fuc_6_ with a β-d-glucosidase (β-Glc, purified from almonds)^51^ and an α-l-fucosidase (AFU, purified from bovine kidney),^12^ respectively (Fig. S11†). By incubation of β-Glc (10 U mL^−1^) with TD-Glc_6_ for 2.5 h, a fluorescence enhancement of 3.7-fold (*Φ*_F_ from 0.010 to 0.037) was achieved (Fig. S11a†). A good linearity was produced by plotting the fluorescence enhancement of TD-Glc_6_ as a function of increasing β-Glc from 0 to 2 U mL^−1^ (Fig. S12a†), and a LOD of 0.01 U mL^−1^ was determined (3*δ*/*k*, Fig. S12b†). This is lower than the previously reported glucosidase probes.^51^ Similarly, TD-Fuc_6_ displayed a fluorescence increase (1.6-fold, *Φ*_F_ from 0.006 to 0.0096) after incubation with AFU (0.12 U mL^−1^) (Fig. S11b†). Meanwhile, the emission intensity of TD-Fuc_6_ enhanced gradually with an increase of AFU concentration from 0 to 0.12 U mL^−1^ (Fig. S13†).

**Fig. 3:**
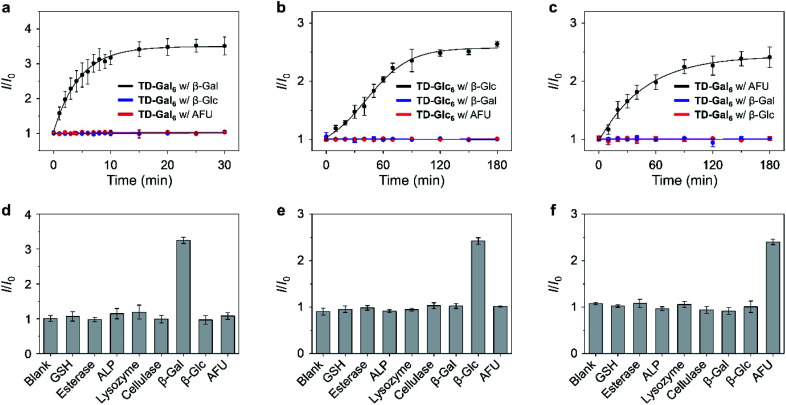
Plotting the fluorescence changes of (a) TD-Gal_6_ (10 μM) with β-Gal (5 U mL^−1^) at pH 4.0, (b) TD-Glc_6_ (10 μM) with β-Glc (5 U mL^−1^) at pH 5.0, and (c) TD-Fuc_6_ (2 μM) with AFU (0.12 U mL^−1^) at pH 7.4 as a function of time. Fluorescence changes of (d) TD-Gal_6_ (10 μM), (e) TD-Glc_6_ (10 μM) and (f) TD-Fuc_6_ (2 μM) with different analytes including GSH (100 μM), esterase (5.0 U mL^−1^), ALP (5.0 U mL^−1^), lysozyme (5.0 U mL^−1^), cellulase (5.0 U mL^−1^), β-Gal (5.0 U mL^−1^), β-Glc (5.0 U mL^−1^) and AFU (0.12 U mL^−1^). *I* and *I*_0_ are the fluorescence intensity of a glycocluster in the presence and absence of an analyte, respectively. All fluorescence spectra were recorded in PBS (0.01 M, pH as indicated) with an excitation at 420 nm.

A kinetic study showed that the fluorescence of the hexavalent AIE-based glycoclusters is enhanced in a time-dependent manner in the presence of the specific glycosidase, including TD-Gal_6_ with β-Gal (Fig. 3a/*A. oryzae* and Fig. S10b†/*E. coli*), TD-Glc_6_ with β-Glc (Fig. 3b) and TD-Fuc_6_ with AFU (Fig. 3c). In contrast, the long-time incubation of the glycoclusters with non-specific glycosidases TD-Gal_6_ with β-Glc/AFU (Fig. 3a and S10b†), TD-Glc_6_ with β-Gal/AFU (Fig. 3b) and TD-Fuc_6_ with β-Gal/β-Glc (Fig. 3c) did not cause the fluorescence of the probes to increase. Then, a broader range of relevant biomolecules including glutathione (GSH), esterase, alkaline phosphatase (ALP), lysozyme (Lyso) and cellulase (Cel) were used to assess the selectivity of the glycoclusters. To our delight, the glycoclusters showed a fluorescence enhancement only in the presence of their specific glycosidase (TD-Gal_6_ with β-Gal (Fig. 3d/*A. oryzae* and Fig. S10c†/*E. coli*), TD-Glc_6_ with β-Glc (Fig. 3e) and TD-Fuc_6_ with AFU (Fig. 3f)), corroborating the good enzyme specificity of the probes.

### Fluorescence imaging of glycosidases in cells with the hexavalent glycoclusters

**Fig. 4:**
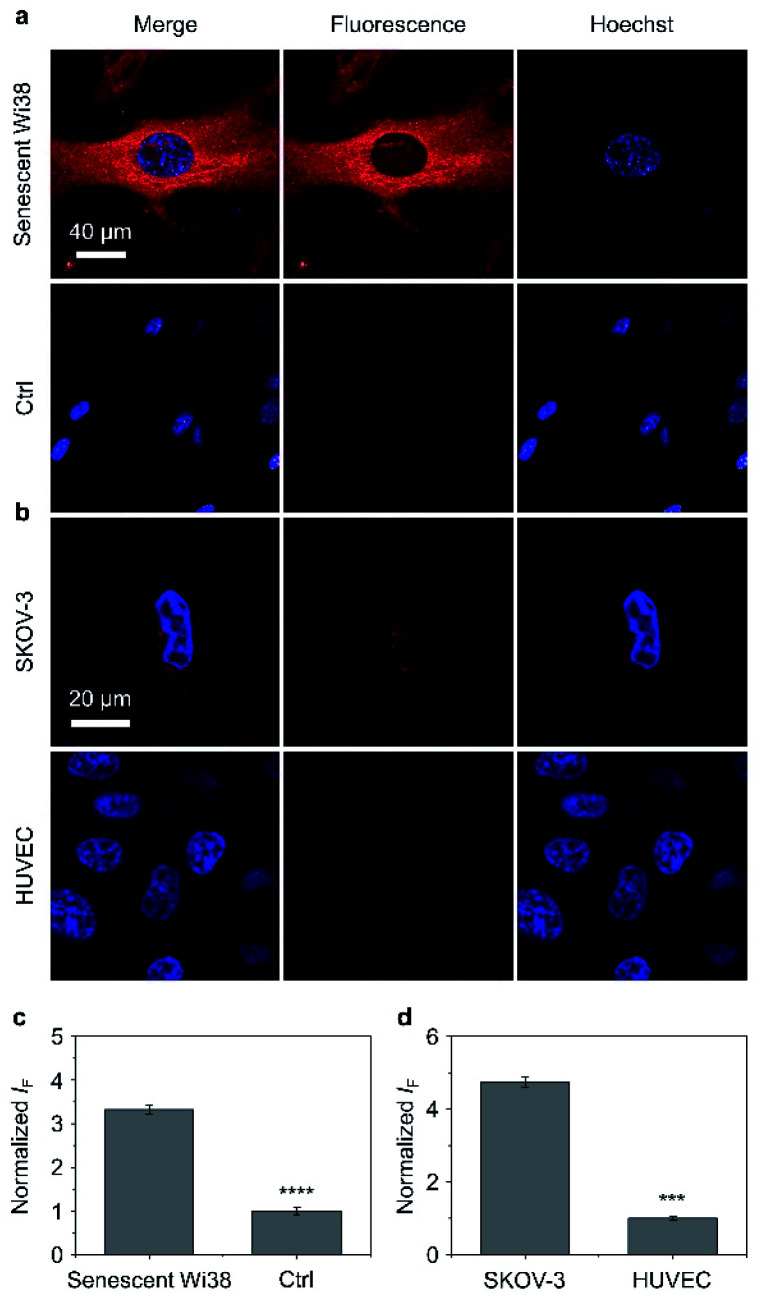
(a) Fluorescence imaging and (c) quantification of senescent Wi38 cells and untreated Wi38 cells (ctrl) after incubation with TD-Gal_6_ (10 μM) for 40 min. (b) Fluorescence imaging and (d) quantification of SKOV-3 (human ovarian carcinoma cells) and HUVEC (human umbilical vein endothelium cells) cells after incubation with TD-Gal_6_ (10 μM) for 40 min. The excitation and emission channels used for TD-Gal_6_ are 488 nm and 580–620 nm, respectively; those for Hoechst 33342 are 405 nm and 440–480 nm, respectively. S. D. means standard deviation (*n* = 3). ***P < 0.001, ****P < 0.0001. Statistical analysis was performed using Student's unpaired *t*-test. All experiments were repeated three times with representative data shown.

Having determined the outstanding sensitivity and selectivity of the AIE-based glycoclusters for glycosidases in solution, we turned our attention to the use of these probes for the fluorescence imaging of glycosidases activity in cells. β-Gal is a secretory protein that is over-expressed during cell senescence^52^ and in human ovarian cancer cells.^53^ The ability to accurately image β-Gal's activity at the cellular level can help advance glycobiology and offer useful tools for cancer diagnosis. Therefore, we examined the cell imaging performances of TD-Gal_6_ for endogenously produced β-Gal in Wi38 (fibroblasts derived from lung tissue) and SKOV-3 (human ovarian cancer) cells. We treated Wi38 cells with H_2_O_2_ leading to oxidative stress-induced cell senescence, and thus an overexpression of lysosomal β-Gal.^3^ For the cell imaging experiment with SKOV-3, HUVEC (human umbilical vein endothelial) cells that hardly express β-Gal were used as control (Fig. 4).^54^

We determined that the fluorescence intensity of TD-Gal_6_ was negligible in untreated Wi38 cells (control) due to the absence of intracellular β-Gal. In contrast, in senescent Wi38 cells treated by H_2_O_2_, an intensive red fluorescence signal corresponding to the de-galactosylated AIE dye was observed (Fig. 4a). Similarly, TD-Gal_6_ produced a strong fluorescence in ovarian cancer cells (SKOV-3 cells, Fig. 4b), but not in HUVEC cells that lack the expression of β-Gal. A subsequent fluorescence quantification showed a 3.4-fold (Fig. 4c) and 4.7-fold (Fig. 4d) stronger fluorescence emission intensity of the glycocluster in senescent Wi38 and SKOV-3 cells than in untreated Wi38 and HUVEC cells, respectively. A cell viability assay showed that TD-Gal_6_ did not impact cell proliferation of SKOV-3 and Wi38 cells even at a concentration of 100 μM, which is 10-fold higher than that used for cell imaging (Fig. S14†). These data suggest the ability of the AIE-based glycocluster for the fluorescence-based imaging of intracellular glycosidase activity.

**Fig. 5:**
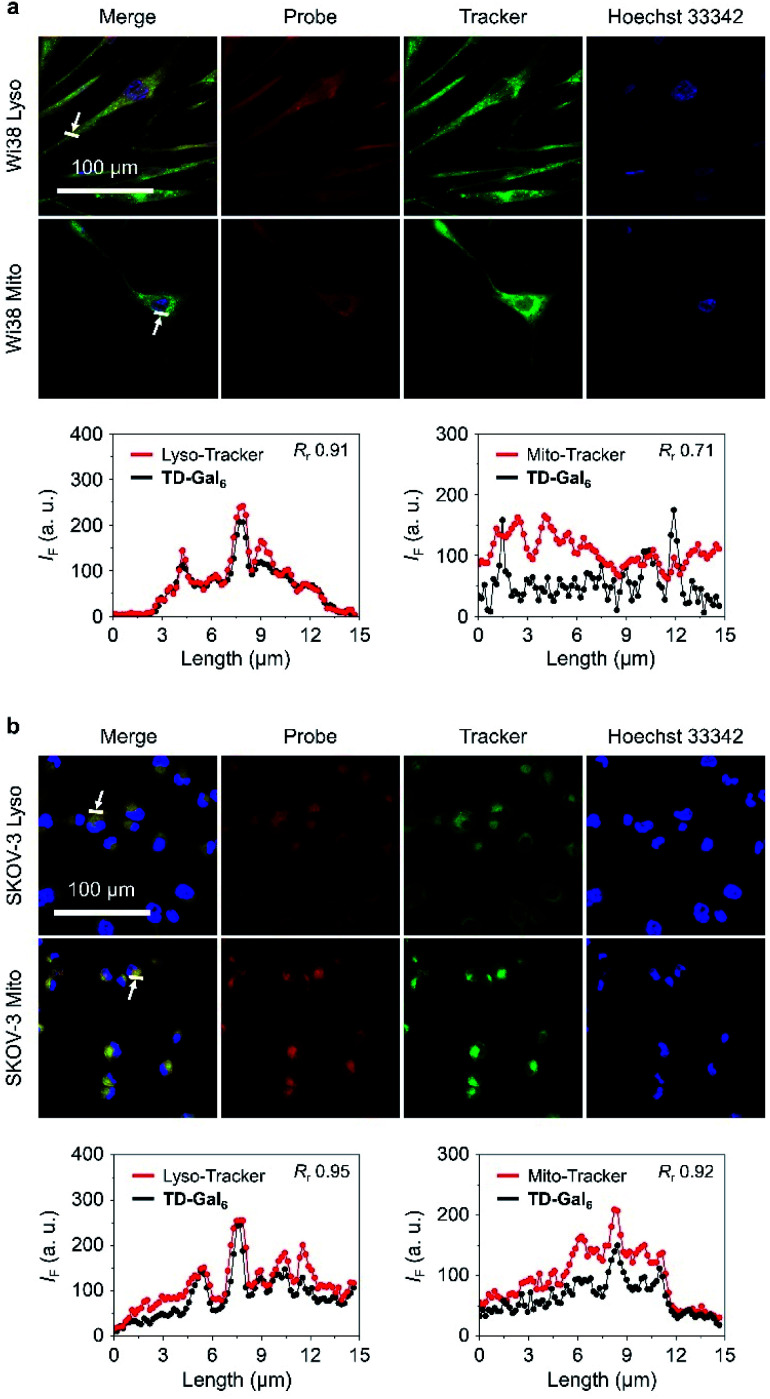
Fluorescence imaging and quantification (the arrow-pointed areas) of (a) senescent Wi38 (fibroblasts derived from lung tissue) cells and (b) SKOV-3 (human ovarian carcinoma) after incubation with TD-Gal_6_ (10 μM) for 4 h. Commercial Mito- and Lyso-Tracker were used for fluorescence co-localization with the probe. The excitation and emission channels used for TD-Gal_6_ are 488 nm and 580–620 nm, respectively; those for Lyso-Tracker are 633 nm and 650–670 nm, respectively; those for Mito-Tracker are 633 nm and 650–670 nm, respectively. *R*_r_ means Pearson's coefficient.

It is reported that the subcellular localization of endogenous β-Gal is different between senescent and ovarian cells.^54^ To test whether TD-Gal_6_ was able to differentiate the subcellular localization of β-Gal in different cell lines, a co-staining assay was carried out with commercial subcellular trackers including a Lyso-Tracker (for lysosomes), Mito-Tracker (for mitochondria) and Hoechst 33342 (for cell nucleus) (Fig. 5). We first observed that, the red fluorescence of the AIEgen produced by *in situ* β-Gal hydrolysis in senescent Wi38 cells overlapped well with the green fluorescence of Lyso-Tracker with a Pearson's coefficient of 0.91 (Fig. 5a). In contrast, a lower Pearson's coefficient of 0.71 was determined by overlapping the AIE fluorescence with that of the Mito-Tracker (Fig. 5a). By co-incubation of the subcellular trackers with TD-Gal_6_ in SKOV-3 cells, a good fluorescence overlap with both organelles was observed (Fig. 5b). A high Pearson's coefficient of 0.95 and 0.92 was determined for the probe with Lyso-Tracker and Mito-Tracker, respectively. These results prove the effectiveness of AIE-based glycoclusters to precisely localize β-Gal activity in cells with different pathological states.

**Fig. 6:**
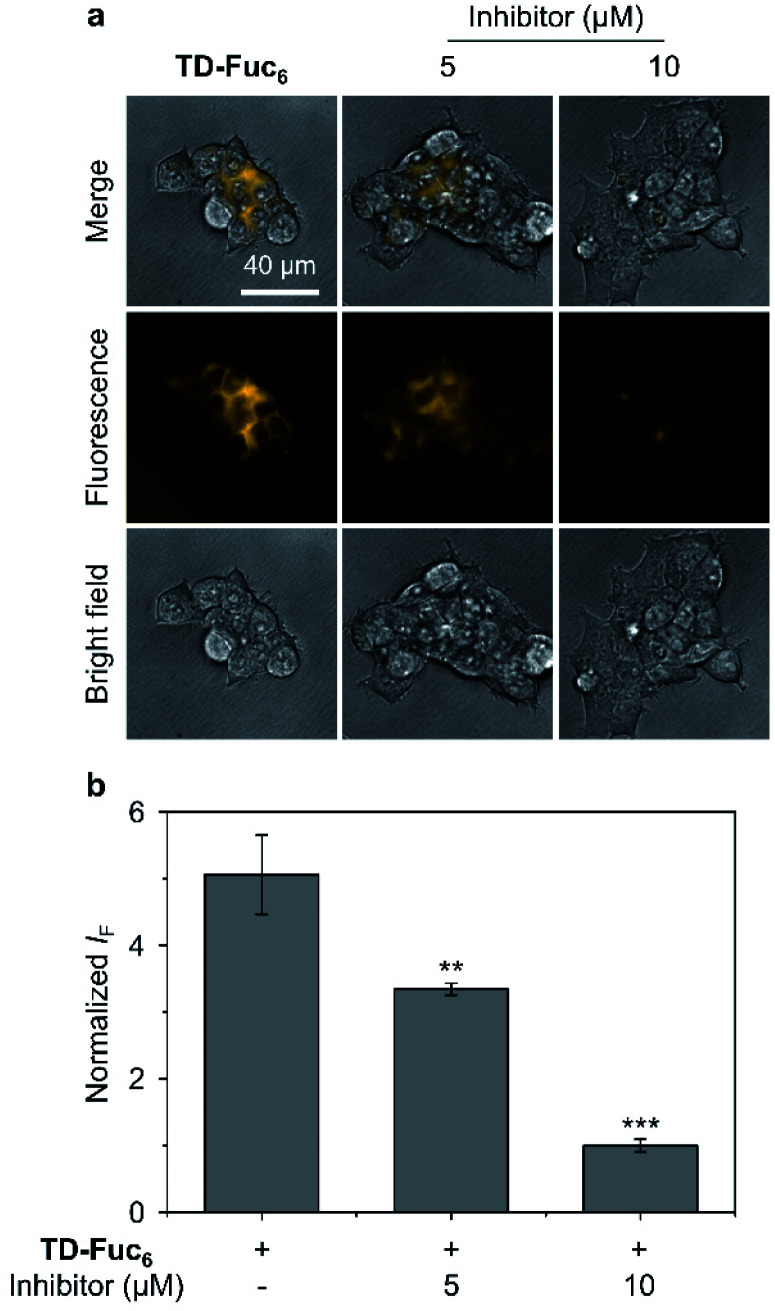
(a) Fluorescence imaging and (b) quantification of 293T (human embryonic kidney cells) cells without and with pre-treatment of a known fucosidase inhibitor (1-deoxyfuconojirimycin) after incubation with TD-Fuc_6_ (10 μM) for 40 min. The excitation and emission channels used for TD-Gal_6_ are 488 nm and 570–630 nm, respectively. S. D. means standard deviation (*n* = 3). **P < 0.01, ***P < 0.001. Statistical analysis was performed using Student's unpaired *t*-test. All experiments were repeated three times with representative data shown.

To demonstrate the scope of the AIE-based glycocluster for glycosidase sensing at the cellular level, we further used TD-Fuc_6_ for probing the endogenous AFU activity in 293T (human embryonic kidneys) cells.^55^ Cells pre-treated with a known AFU inhibitor (1-deoxyfuconojirimycin)^56^ were used as control. To our delight, an intensive fluorescence emission corresponding to the residual AIEgen of TD-Fuc_6_ after removal of the fucosyl epitopes was observed in the cytoplasm of 293T cells (Fig. 6a). However, the fluorescence intensity of the probe decreased gradually with increasing AFU inhibitor (5 μM and 10 μM) (Fig. 6). This suggests that the fluorescence production of TD-Fuc_6_ in 293T cells is dependent on the intracellular AFU activity. In addition, cell viability assay illustrated that TD-Fuc_6_ did not impact cell proliferation of 293T for 72 h incubation even at a concentration of 100 μM (Fig. S15†). A survey of previous literature reports demonstrates a scarcity in the achievement of fluorescence-based imaging of AFU activity in live cells. This is probably owing to the difficulty in the synthesis of fluorogenic α-l-fucosyl probes through direct phenol glycosylation. The successful development of TD-Fuc_6_ offers a powerful imaging tool for the in-depth study of AFU-relevant biological and pathological processes in different types of mammalian cells.

**Fig. 7:**
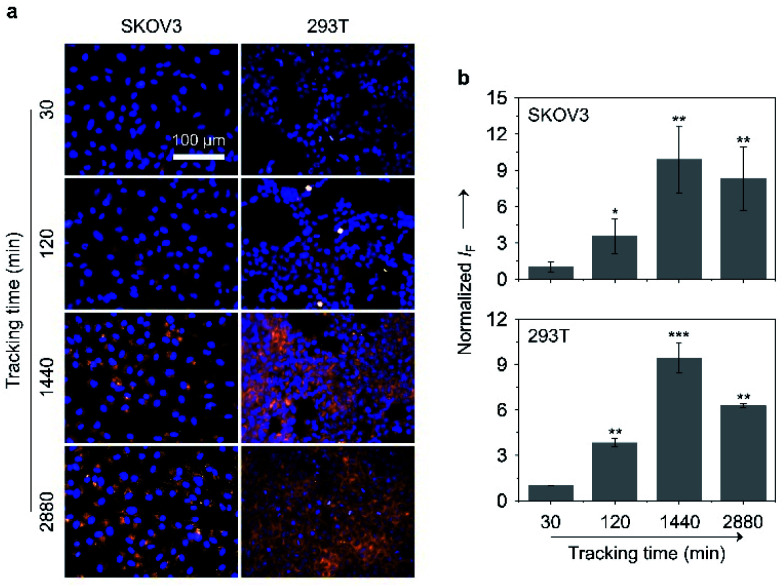
(a) Fluorescence imaging and (b) quantification of SKOV-3 (human ovarian carcinoma cells) and 293T (human embryonic kidney cells) cells after incubation with TD-Gal_6_ (10 μM) and TD-Fuc_6_ (10 μM), respectively, with time. The excitation and emission channels used for TD-Gal_6_/TD-Fuc_6_ are 488 nm and 580–620 nm, respectively; those for Hoechst 33342 are 405 nm and 440–480 nm, respectively. S. D. means standard deviation (*n* = 3). *P < 0.1, **P < 0.01, ***P < 0.001. Statistical analysis was performed using Student's unpaired *t*-test. All experiments were repeated three times with representative data shown.

Large-sized aggregates and particles were shown to have a slower exocytotic rate than dispersed small molecules due to a prolonged intracellular retention thanks to the size effect.^57–59^ Given the AIE feature of the glycoclusters, we also tested their cell imaging capacity with time (Fig. 7). After treating SKOV-3 cells with TD-Gal_6_ for 30 min, a relatively weak fluorescence signal was observed probably due to the insufficient de-glycosylation of the glycoclusters intracellularly. This fluorescence was then enhanced when the incubation time was increased to 120 min, suggesting an increase in the proportion of de-glycosylated AIEgens. After a further prolonged incubation time of 1440 min (24 h), a *ca.* 9.9-fold increase in fluorescence intensity was observed with respect to the group with the incubation time of 30 min. Finally, the fluorescence was slightly reduced when the incubation time was increased to 2880 min (48 h). Likewise, a similar trend in fluorescence change was observed for TD-Fuc_6_ in 293T cells over time. The fluorescence of the probe maximized in 1440 min with a *ca.* 9.5-fold increase in fluorescence intensity with respect to the initial 30 min group, and then slightly decreased with a further prolonged incubation time of 2880 min. These data suggest that our glycosidase-responsive AIE-based glycoclusters are amenable to (1) monitoring the enzymatic activity of different glycosidases intracellularly, and (2) imaging of cells for more than 48 hours because of a slow exocytosis rate of the de-glycosylated AIEgen aggregates. The first point is particularly useful for the interrogation of the de-glycosylation process of a glycosidase of interest that is overly expressed during a given cellular process.

## Conclusions

In summary, to overcome the synthetic limitations associated with previously reported fluorogenic glycosidase probes, we have developed AIE-based glycoclusters with excellent sensitivity and selectivity as a platform strategy for sensing the activity of diverse glycosidases in solution and in cells. A comparison of the sensing performances among four series of structurally different glycoclusters identified hexavalent TPE–DCM-based glycoclusters as the optimal structural motif for glycosidase sensing through AIE. The synthetic strategy avoids the direct glycosylation on phenol group of D–A-type fluorogens, leading to the acquisition of structurally more diverse glycosidase substrates based on the simple and efficient click coupling between an alkynylated TPE–DCM core and azido glycosides. Upon enzymatic hydrolysis of the glycosyl epitopes, the resultant amphiphilic AIEgens aggregated in water thereby producing a drastically enhanced, stable fluorescence signal.

The AIE-based glycoclusters achieved (1) the fluorogenic detection of different glycosidases including galactosidase, glucosidase and fucosidase with excellent selectivity over a number of other enzymes that exist in the human body, (2) fluorescence-based imaging of the endogenous glycosidase activity in different cell types through intracellular de-glycosylation producing aggregated AIEgens, (3) differentiation of the subcellular localization of a galactosidase between cancer and senescent cells, and (4) study of the kinetics of the de-glycosylation action of different glycosidases, and the subsequent long-term fluorescent imaging of cells thanks to the slow exocytosis of AIEgen aggregates. Our research offers new insight into the development of AIE-based fluorogenic glycoclusters for glycosidase sensing, but addresses the scarcity in universal functional imaging tools for glycosidases, in particular fucosidases that have been identified to be useful cancer biomarkers.^60^

## Data availability

Data for the synthesis and fluorescence properties of the designed molecules are available in the ESI.† Raw NMR data are available upon request.

## Author contributions

LG, MYZ, HHH synthesized the fluorescent probes and performed the fluorescence experiments *in vitro* and in cell assays, also described the experimental sections and drafted portions of the full text manuscript and ESI.† YZ, GRC, JL, XPH and SV directed the project, organized the data and discussion, drafted the text of the manuscript and ESI.†

## Conflicts of interest

The authors declare no competing financial interest.

## Supplementary Material

SC-013-D1SC05057E-s001
