# Supplementary material for: A general strategy to the intracellular sensing of glycosidases using AIE-based glycoclusters
Source: Chem Sci. 2021 Dec 7;13(1):247–56. doi: 10.1039/d1sc05057e (PMC8694377; doi:10.1039/d1sc05057e)
Supplement: SC-013-D1SC05057E-s001 [file SC-013-D1SC05057E-s001.pdf]

Electronic Supplementary Material (ESI) for Chemical Science.
This journal is © The Royal Society of Chemistry 2021

Supporting Information

A General Strategy for the Intracellular Sensing of Glycosidases using AIE-Based Glycoclusters

Lei Dong,[a,b,‡] Min-Yu Zhang,[a,‡] Hai-Hao Han,[a,‡] Yi Zang,[c] Guo-Rong Chen,[a] Jia Li,[c,*] Xiao-Peng He[a,*] and Sébastien Vidal[b,d,*]

[a] Key Laboratory for Advanced Materials and Joint International Research Laboratory of Precision Chemistry and Molecular Engineering, Feringa Nobel Prize Scientist Joint Research Center, Frontiers Center for Materiobiology and Dynamic Chemistry, School of Chemistry and Molecular Engineering, East China University of Science and Technology, 130 Meilong Rd., Shanghai 200237, P.R. China.

[b] Institut de Chimie et Biochimie Moléculaires et Supramoléculaires, Laboratoire de Chimie Organique 2-Glycochimie, UMR 5246, CNRS and Université Claude Bernard Lyon 1, Université de Lyon, 1 Rue Victor Grignard, F-69622 Villeurbanne, France.

[c] National Centre for Drug Screening, State Key Laboratory of Drug Research, Shanghai Institute of Materia Medica, Chinese Academy of Sciences, 189 Guo Shoujing Rd., Shanghai 201203, PR China.

[d] Université Paris-Saclay, CNRS, Institut de Chimie des Substances Naturelles, UPR 2301, 91198, Gif-sur-Yvette, France.

Content List

**Additional figures, schemes and tables**	S1-S11
**General remarks**	S12
**Synthesis of TPE-DCM based glycoclusters and NMR spectra**	S12-S48
**Photophysical and biological measurements**	S49-S50

Additional figures and schemes

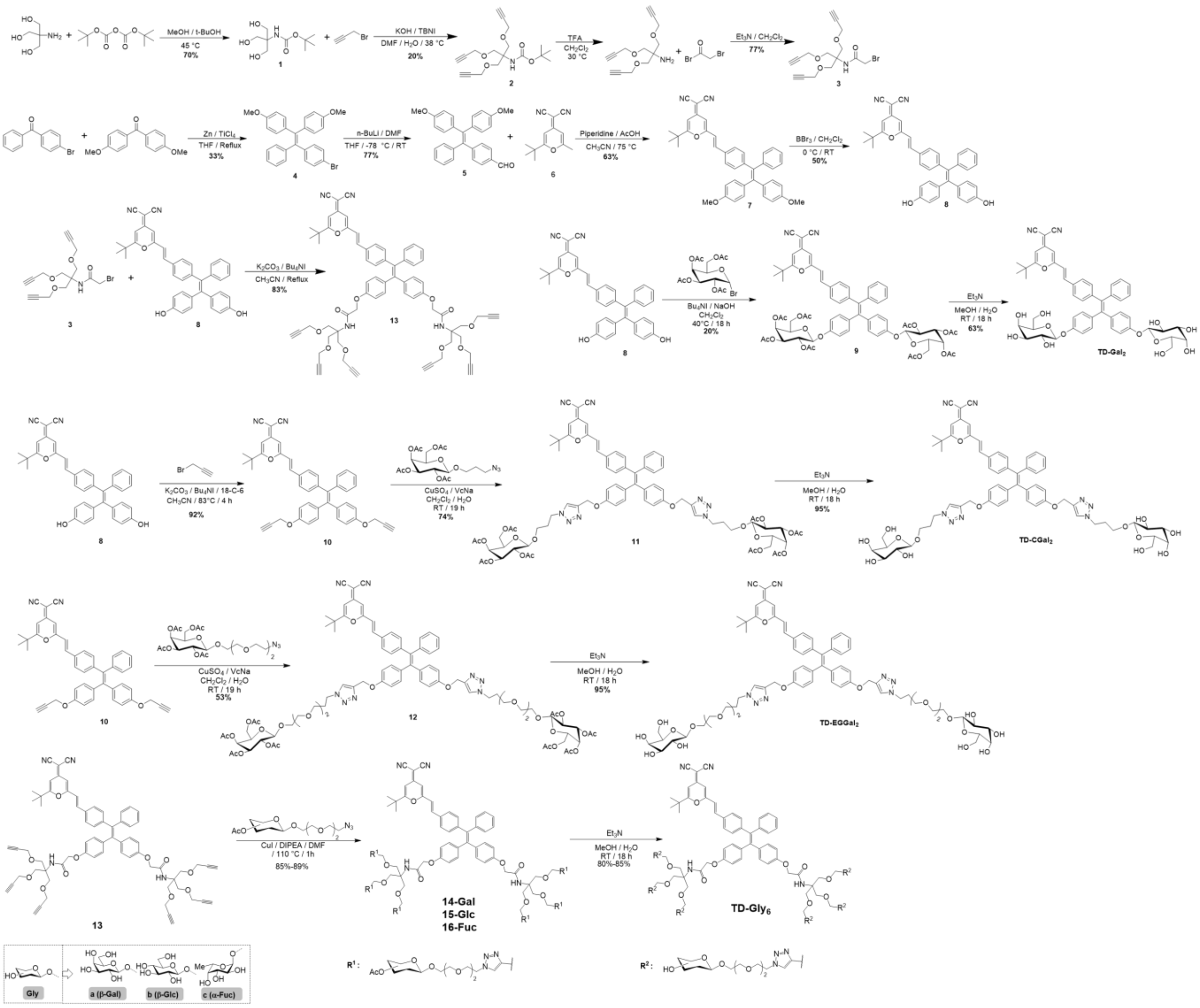

Scheme S1. Synthesis of the **TPE-DCM** based fluorescent glycoclusters.

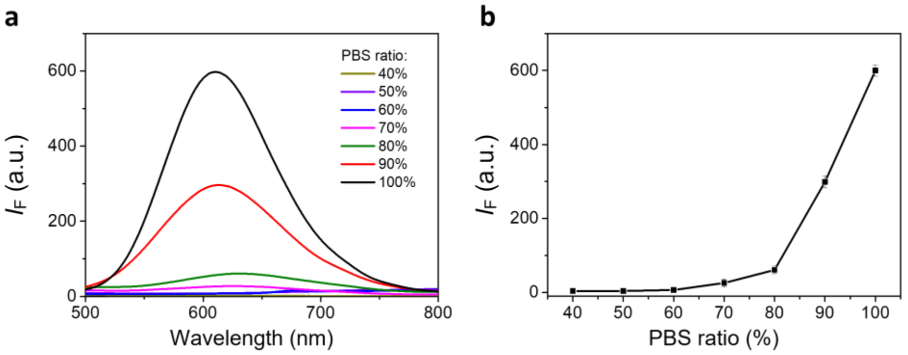

Figure S1. (a) Fluorescence emission spectra of compound **7** (10 μM) in THF with increasing PBS buffer (0.01 M, pH 7.4) ratio (from 40% to 100%), and (b) Plotting the fluorescence intensity of compound **7** (10 μM) in THF with increasing PBS ratio (from 40% to 100%) (λ_{ex} = 420 nm).

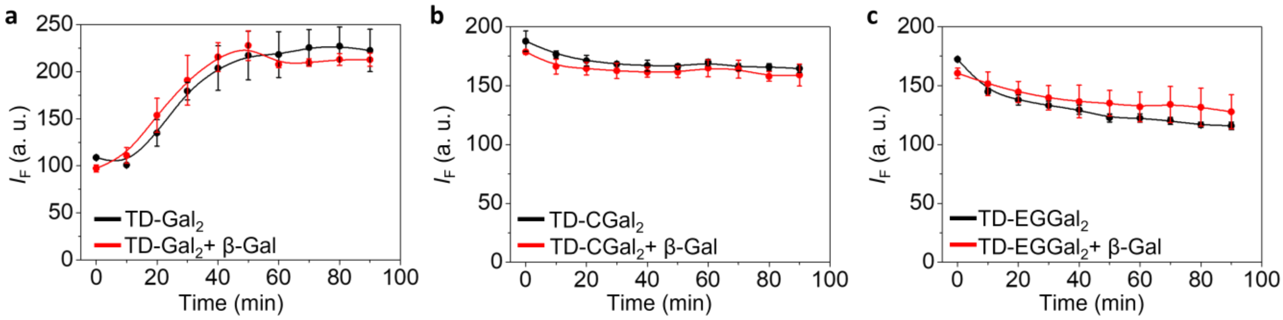

Figure S2. Fluorescence emission intensity changes of (a) **TD-Gal$_2$** (10 μM), (b) **TD-CGal$_2$** and (c) **TD-EGGal$_2$** in the absence (black) and presence (red) of β-Gal (5 U mL^{-1}) from *E. coli* in PBS (0.01 M, pH 7.4) with time (λ_{ex} = 420 nm).

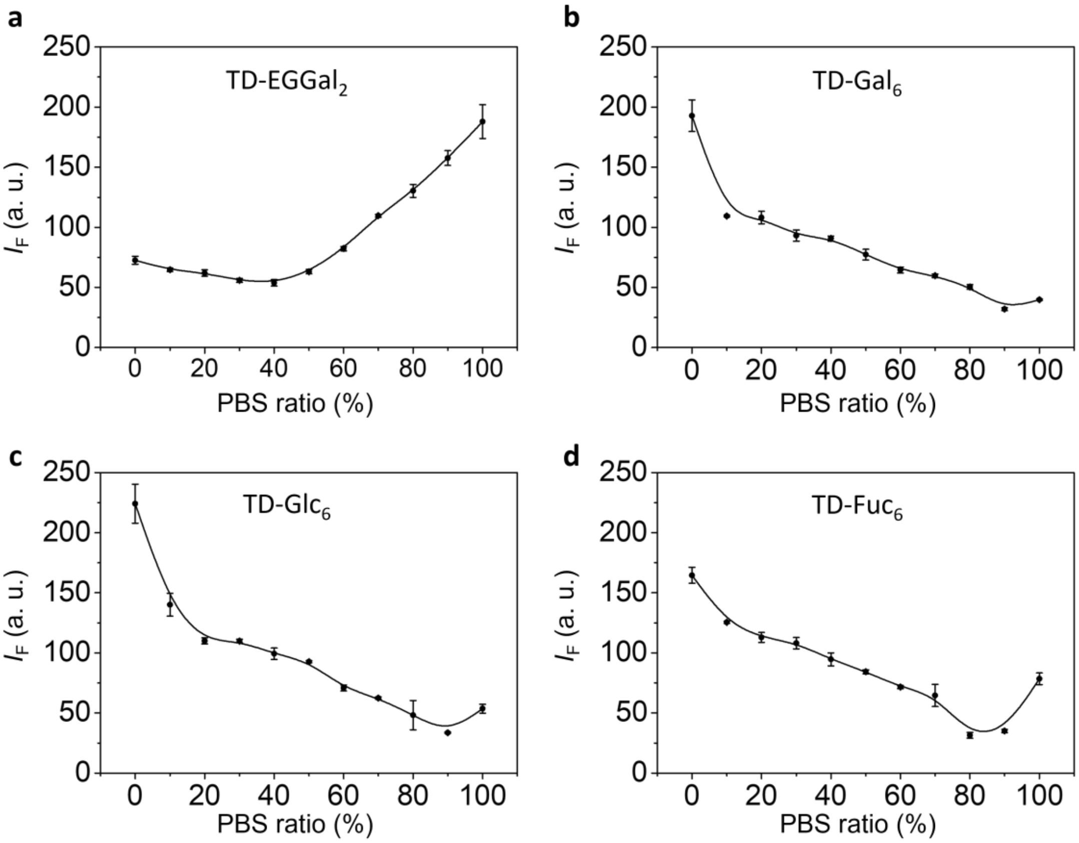

Figure S3. Plotting the fluorescence emission intensity of (a) **TD-EGGal$_2$**, (b) **TD-Gal$_6$**, (c) **TD-Glc$_6$** and (d) **TD-Fuc$_6$** in a solvent mixture of THF/PBS (0.01 M, pH 7.4) at different mixing ratios ([**TD-Gly$_6$**]= 10 μM, λ_{ex} = 420 nm).

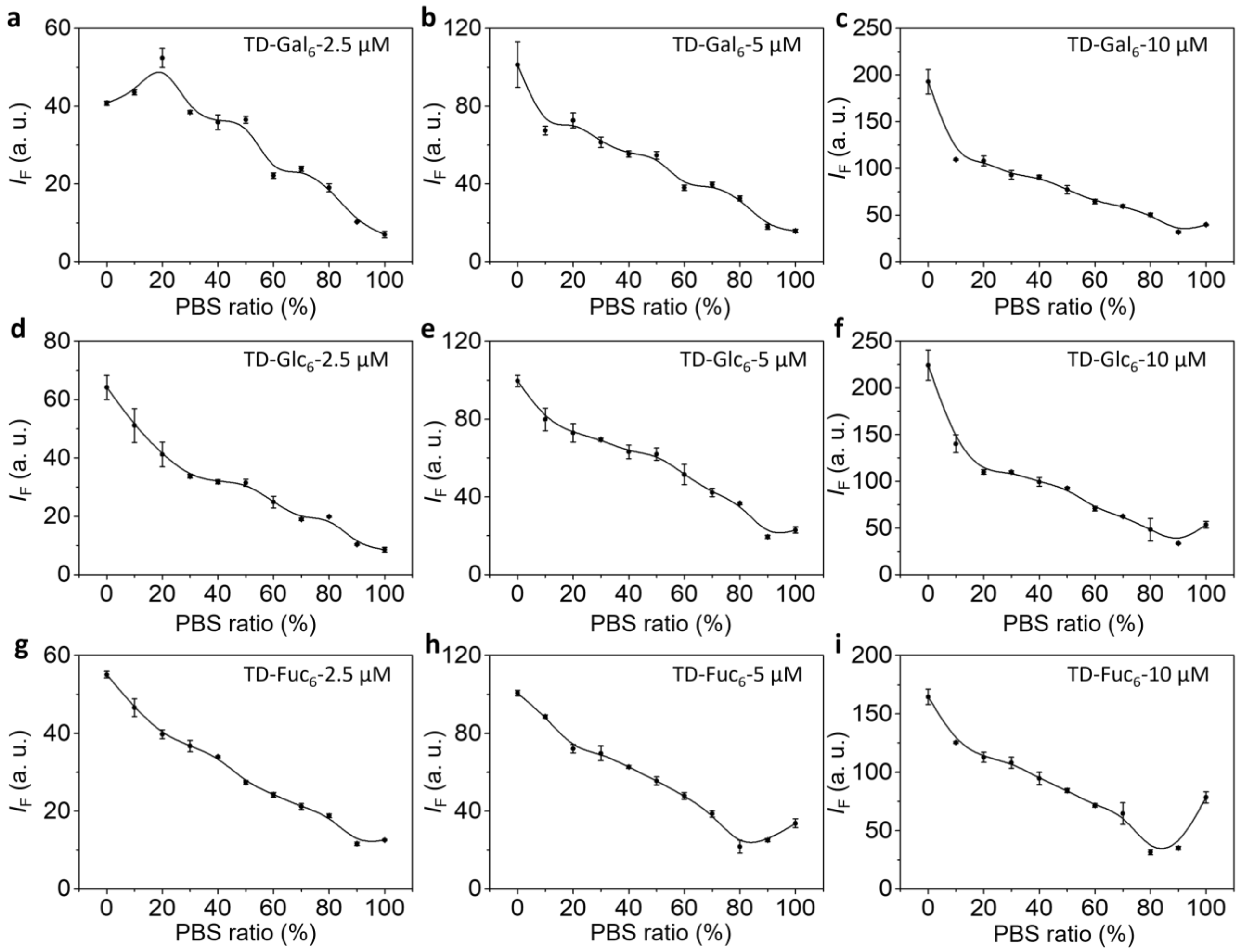

Figure S4. Plotting the fluorescence emission intensity of (a-c) **TD-Gal$_6$**, (d-f) **TD-Glc$_6$** and (g-i) **TD-Fuc$_6$** at different concentrations (2.5, 5 and 10 μM) in a solvent mixture of THF/PBS (0.01 M, pH 7.4) at different mixing ratios (λ_{ex} = 420 nm).

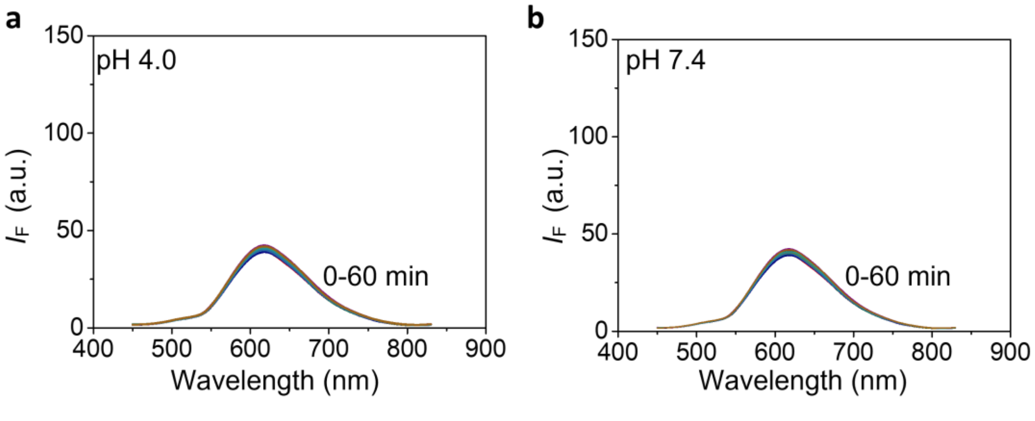

Figure S5. Fluorescence stability of **TD-Gal$_6$** (10 μM) in PBS (0.01 M, pH 4.0 or 7.4) during 60 min incubation without β-Gal (λ_{ex} = 420 nm).

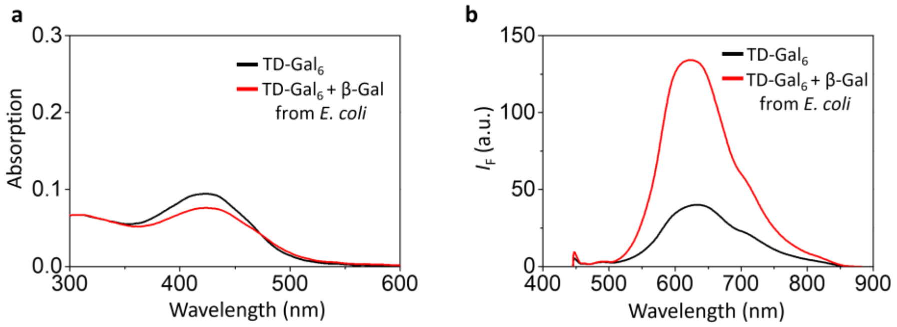

λmax(absorption)	λmax(emission)	Stokes shift
420 nm	625 nm	225 nm

	Quantum yield (Φ)	
**TD-Gal$_6$**	0.005	5-folds
**TD-Gal$_6$** + β-Gal	0.026	

Figure S6. (a) UV-vis absorption and (b) fluorescence emission spectra of **TD-Gal6** (10 μM) in presence of β-Gal (10 U mL^{-1}) from *E. coli* in PBS (0.01 M, pH 7.4) (λ_{ex} = 420 nm).

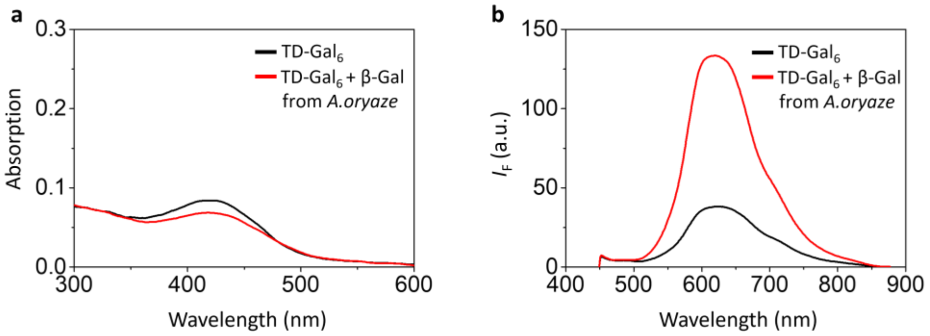

λmax(absorption)	λmax(emission)	Stokes shift
420 nm	625 nm	225 nm

	Quantum yield (Φ)	
**TD-Gal$_6$**	0.006	4.5-folds
**TD-Gal$_6$** + β-Gal	0.027	

Figure S7. (a) UV-vis absorption and (b) fluorescence emission spectra of **TD-Gal6** (10 μM) in presence of β-Gal (10 U mL^{-1}) from *A. oryaze* in PBS (0.01 M, pH 4.0) with (λ_{ex} = 420 nm).

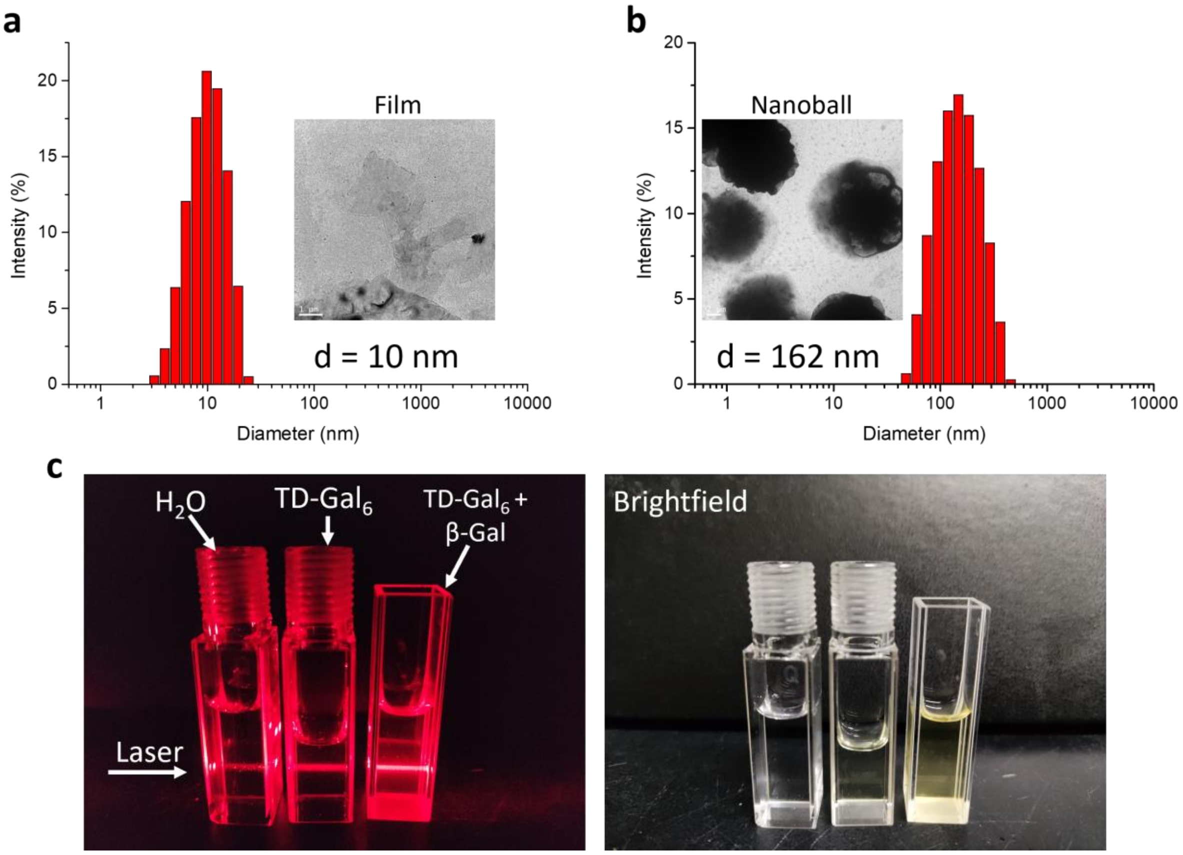

Figure S8. Dynamic light scattering and TEM picture (inset) of **TD-Gal6** (10 μM) before (a) and after (b) incubation with β-Gal (5 U mL^{-1}) from *E. coli* for 50 min at 37°C in PBS (0.01 M, pH 7.4). (c) Tyndall effect of the different solutions under 630 nm laser.

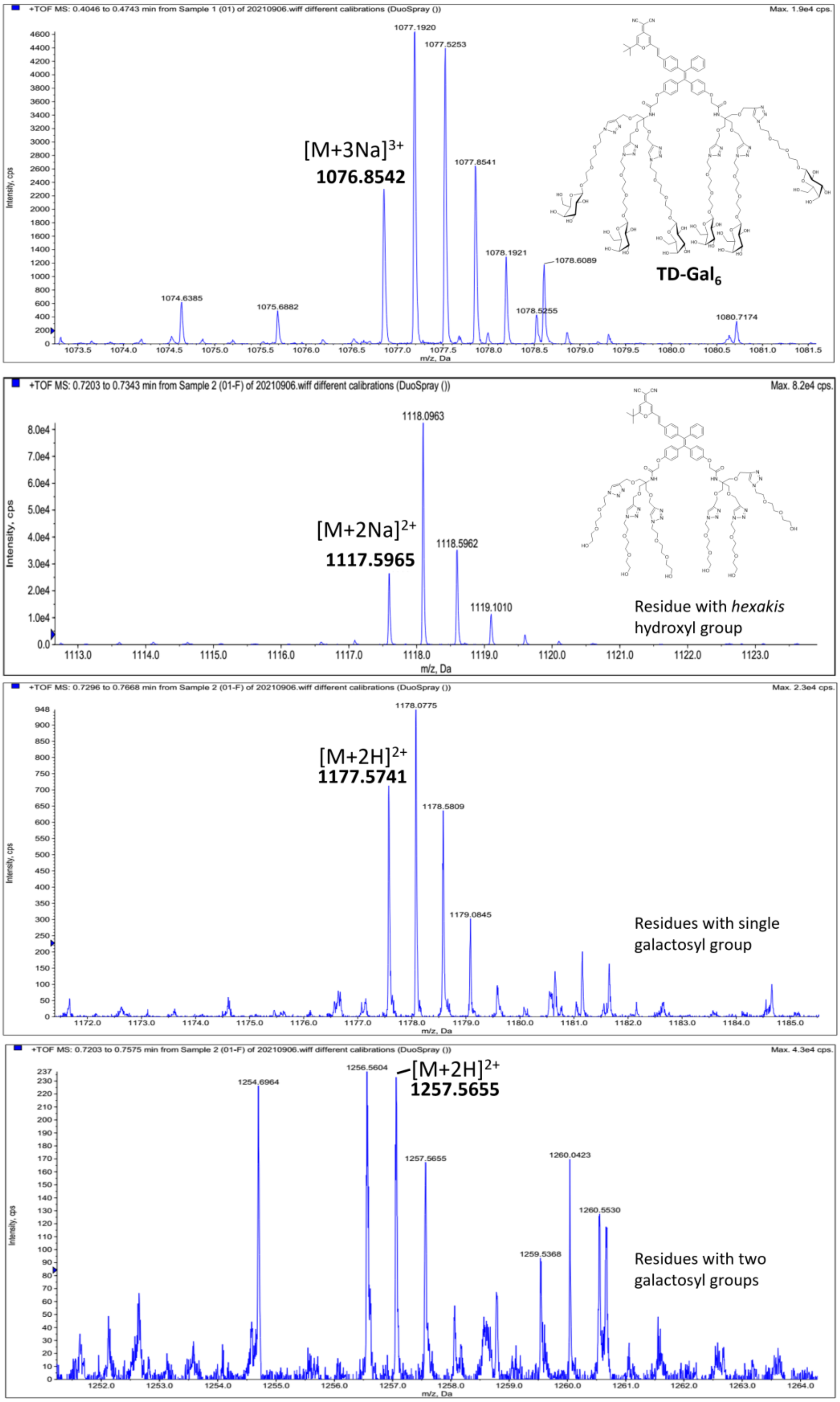

Figure S9. Mass spectrometry of **TD-Gal₆** and the different residual products of **TD-Gal₆** after incubating with β-Gal (5 U mL^{-1}) from *E. coli* for 50 min in PBS (0.01 M, pH 7.4).

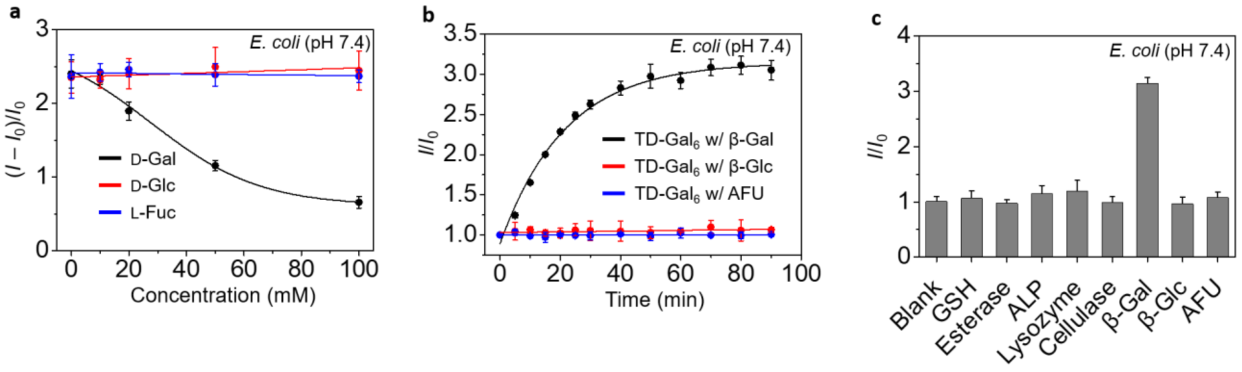

Figure S10. (a) Plotting the fluorescence emission intensity changes of **TD-Gal₆** with β-Gal (5 U mL^{-1}) as a function of increasing free D-Gal, D-Glc and L-Fuc. (b) Plotting the fluorescence emission intensity changes of **TD-Gal₆** in the presence of β-Gal (5 U mL^{-1}) with time. (c) Fluorescence emission intensity changes of **TD-Gal₆** incubating with different analytes. ([**TD-Gal₆**] = 10 μM, β-Gal from *E. coli*, PBS 0.01 M, pH 7.4, λ_{ex} = 420 nm). I_0 and I are the fluorescence emission intensity of the probe without and with an analyte, respectively.

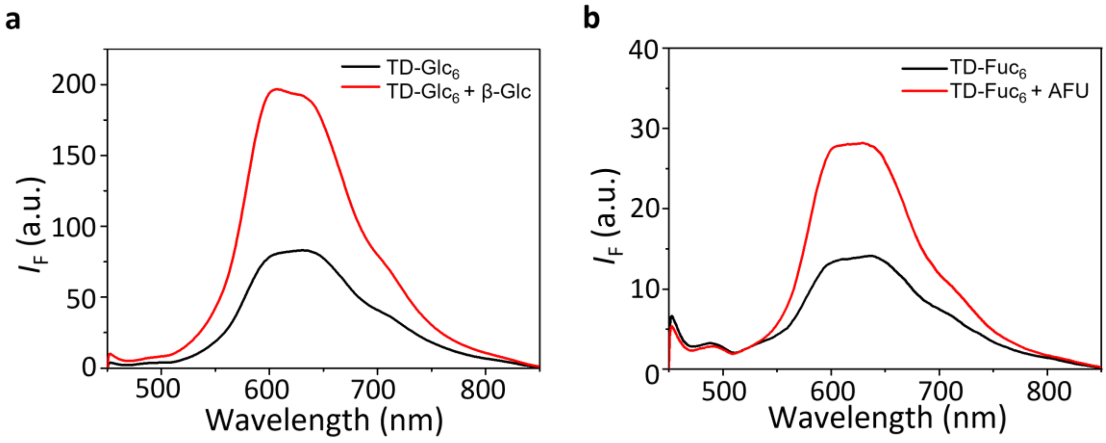

Figure S11. Fluorescence emission spectra of (a) **TD-Glc₆** (10 μM) without and with incubation of β-Glc (10 U mL^{-1}) at 60 °C for 2.5 h in PBS (0.01 M, pH 5.0). (b) **TD-Fuc₆** (2 μM) without and with incubation of AFU (0.12 U mL^{-1}) at 37 °C for 3.5 h in PBS (0.01 M, pH 7.4) (λ_{ex} = 420 nm).

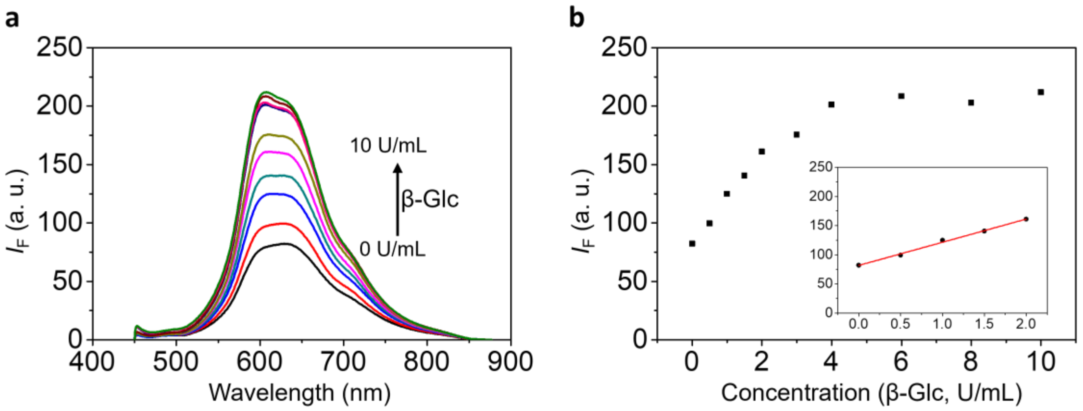

Figure S12. (a) Fluorescence emission spectra of **TD-Glc₆** (10 μM) with increasing β-Glc in PBS (0.01 M, pH 5.0) (λ_{ex} = 420 nm). (b) Plotting the fluorescence emission intensity of **TD-Glc₆** as a function of β-Glc concentration.

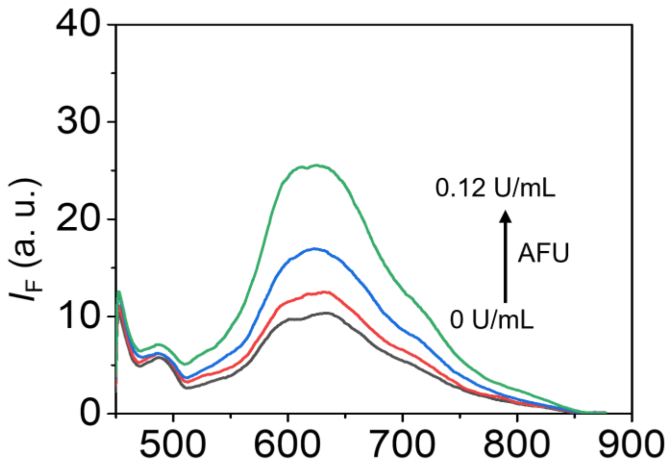

Figure S13. Fluorescence emission spectra of **TD-Fuc₆** (2 μM) with increasing AFU in PBS (0.01 M, pH 7.4) (λ_{ex} = 420 nm).

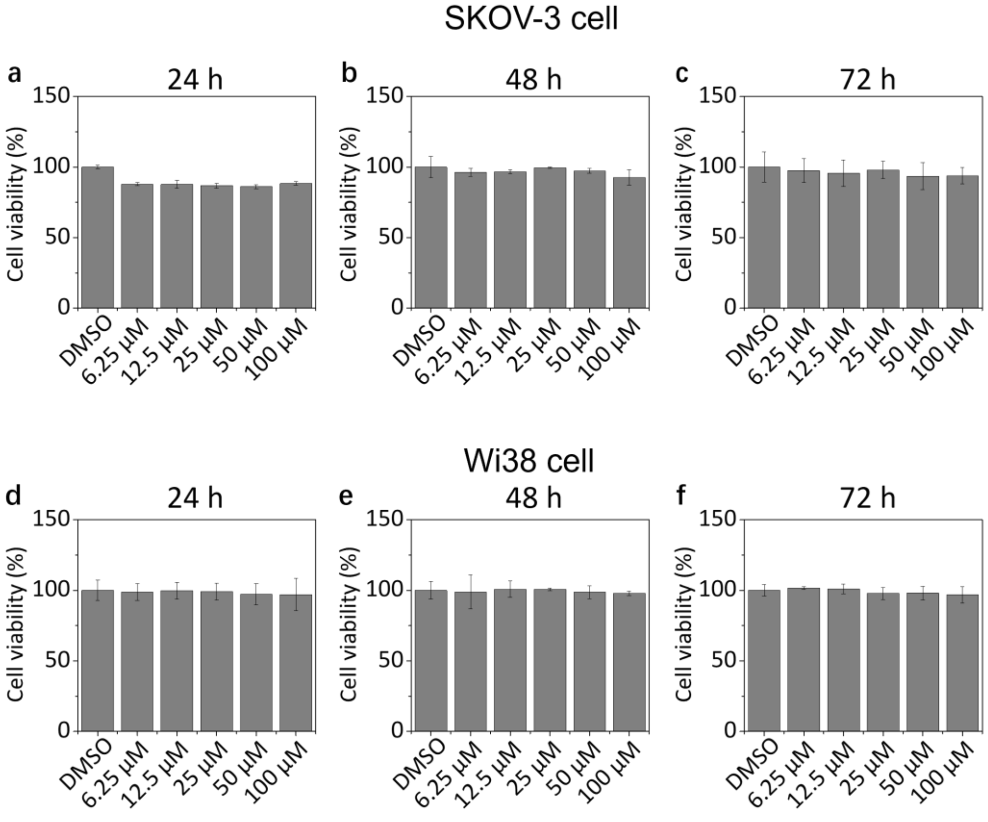

Figure S14. Cell viability of (a-c) SKOV-3 (human ovarian cancer) and (d-f) Wi38 (fibroblasts derived from lung tissue) cells after incubation with different concentrations of **TD-Gal$_6$** for (a, d) 24 h, (b, e) 48 h, and (c, f) 72 h. S. D. means standard deviation (n = 3).

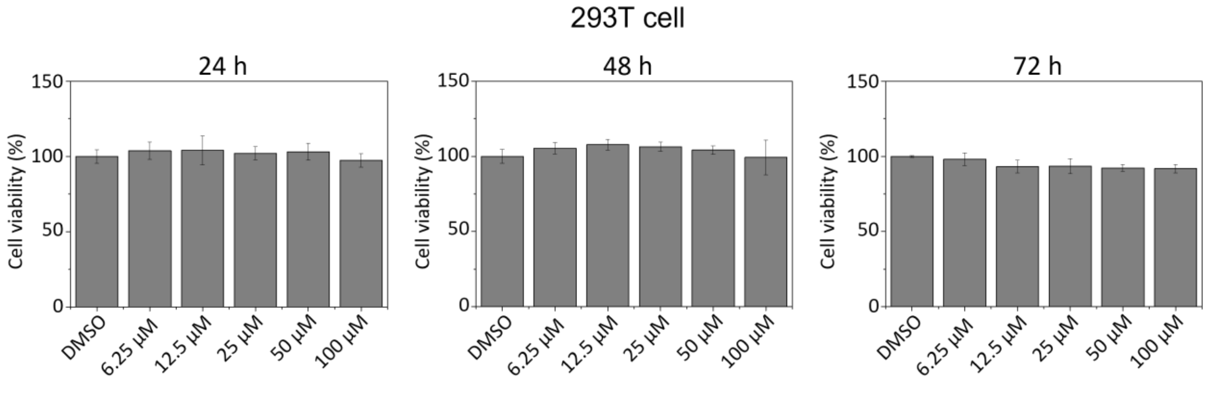

Figure S15. Cell viability of 293T cells (human embryonic kidneys) after incubation with different concentrations of **TD-Fuc$_6$** for 24 h, 48 h, and 72 h. S. D. means standard deviation (n = 3).

Table S1. Fluorogenic probes for β-Gal

CHEMICAL STRUCTURE	Λ_{EX} / Λ_{EM}	SOLUTION	LOD	REFERENCE
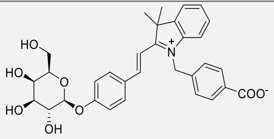	435 / 536	PBS	0.0083 U/mL	*Angew. Chem. Int. Ed.* **2021**, *60*, 10756
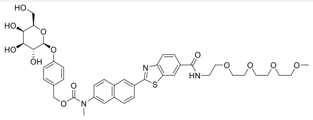	378 / 540	PBS	2.0 nM	*Anal. Chem.* **2014**, *86*, 10001
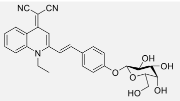	434 / 560<br>AIE	PBS / DMSO (5%)	0.001 U/mL	*Chem. Sci.*, **2019**, *10*, 398
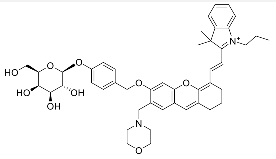	690 / 720	PBS / DMSO (20%)	0.022 U/mL	*Anal. Chem.* **2020**, *92*, 5772
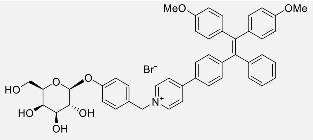	344 / 512<br>AIE	PBS	0.33 U/mL	*Chem. Commun.* **2017**, *53*, 4505
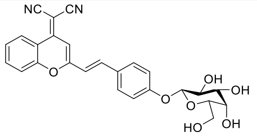	450 / 685	PBS / DMSO (30%)	1.7×10^{-4} U/mL	*J. Am. Chem. Soc.* **2016**, *138*, 5334
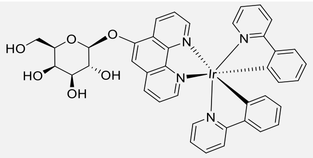	282 / 586	PBS	0.51 U/mL	*Anal. Chem.* **2017**, *89*, 11679
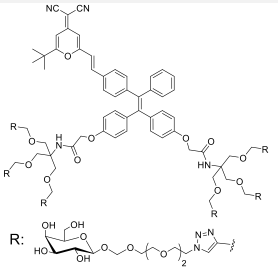	420 / 625<br>AIE	PBS	0.015 U/mL for *E. Coli*<br>0.014 U/mL for *A.oryaze*	**This work**

General remarks

All reagents for synthesis commercially available (highest purity available for reagent grade compounds) were used without further purification. The glycosidases *in vitro* experiments are commercia without purification. The β-galactosidases (EC 3.2.1.23) used are purified from *Escherichia coli* (*E. coli*) or *Aspergillus oryzae* (*A.oryzae*), β-glycosidase (EC 3.2.1.21) from almonds, and α-fucosidase (AFU, EC 3.2.1.51) from bovine kidney. Reactions under microwave activation were performed on a Biotage Initiator system. Thin-layer chromatography (TLC) was carried out on aluminum sheets coated with silica gel 60 F_{254} (Merck). TLC plates were inspected by UV light (λ = 254 nm, 365 nm) and developed by treatment with a mixture of 10% H_2SO_4 in EtOH/H_2O (95:5 v/v) followed by heating. Silica gel column chromatography was performed with silica gel Si 60 (40–63 µm). NMR spectra were recorded at 293 K, unless stated otherwise. Chemical shifts are referenced relative to deuterated solvent residual peaks. The following abbreviations are used to explain the observed multiplicities: s, singlet; d, doublet; t, triplet; q, quadruplet; m, multiplet; p, pseudo and b, broad. Complete signal assignments were based on 1D and 2D NMR correlations COSY and HSQC. High resolution (HR-ESI-QToF) mass spectra were recorded using a Bruker MicroToF-Q II XL spectrometer.

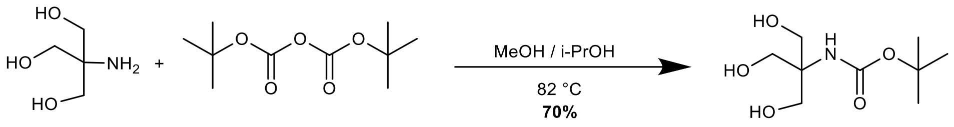

1

M Rao, K Kanagaraj, CY Fan, JC Ji, C Xiao, XQ Wei, *et al. Org. Lett.* **2018**, *20*, 1680-1683

N-(*t*-butyloxycarbonyl)tris(hydroxymethyl)aminomethane (1):

A suspension of tris(hydromethyl)aminomethane (4 g, 33 mmol, 1 eq.) and di-*t*-butyldicarbonate (9.4 g, 43 mmol, 1.3 eq.) in MeOH/*i*-PrOH (30 mL, 2:1, v/v) was stirred at 82°C during 18 h. The reaction was then evaporated when all starting material had been dissolved. The solid residue was diluted with EtOAc (50 mL) then filtered and washed with EtOAc (3×40 mL) and dried under vacuum to obtain compound **1** (5.05 g, 70%) as a white powder.

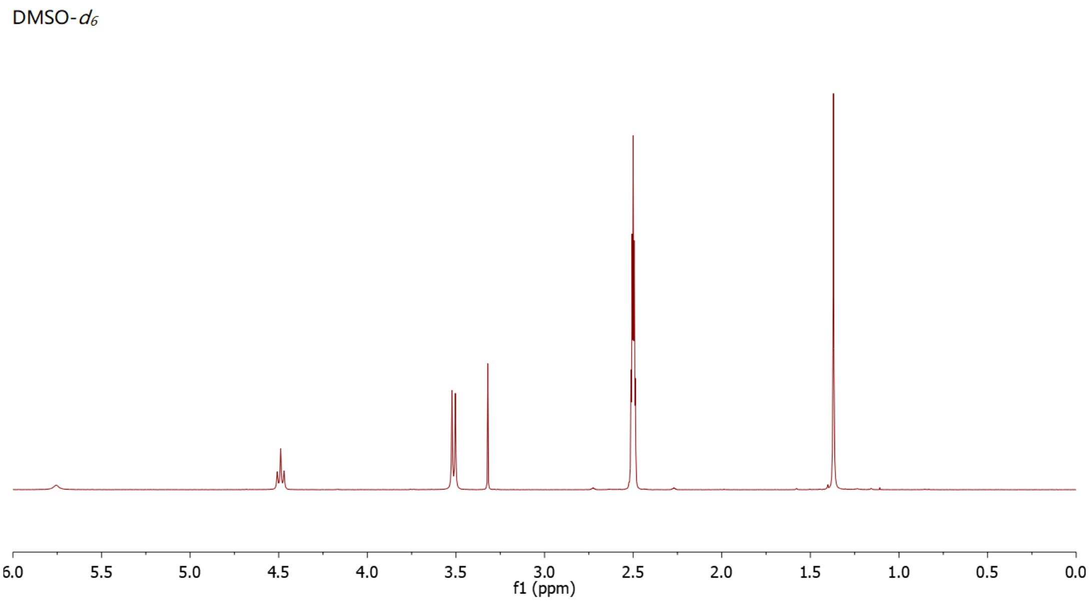

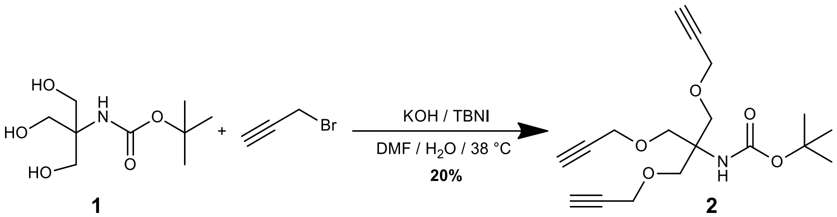

M Rao, K Kanagaraj, CY Fan, JC Ji, C Xiao, XQ Wei, *et al. Org. Lett.* **2018**, *20*, 1680-1683

_N_-(_t_-butyloxycarbonyl)tris(propargyloxymethyl)aminomethane (2):

To a solution of compound **1** (1 g, 4.5 mmol, 1 eq.), potassium hydroxide (KOH, 1.5 g, 27.3 mmol, 6 eq.) and tetrabutylammonium iodide (360 mg, 0.45 mmol, 0.1 eq.) in DMF/H_2O (18 mL, 5:1, v/v) was added propargyl bromide (2.15 g, 18.1 mmol, 4 eq.). The resulting solution was stirred at 38°C during 20 h. The solution was diluted with EtOAc (50 mL), washed with brine (2×40 mL) and HCl aqueous solution (20 mL, 2M). The organic layer was dried (Na_2SO_4), concentrated and purified by silica gel column chromatography (Cyclohexane:EtOAc = 4:1) to obtain compound **2** (285 mg, 20%) as a pale yellow oil.

R_f = 0.57 (Cyclohexane:EtOAc = 4:1)

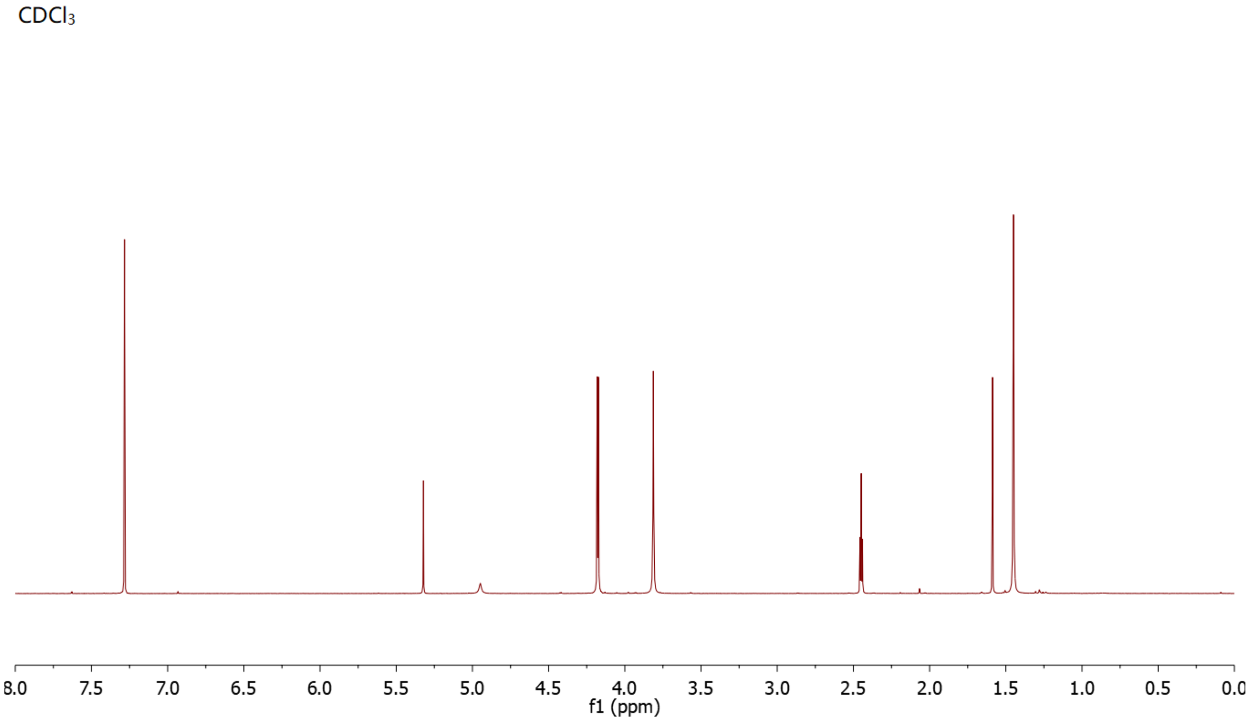

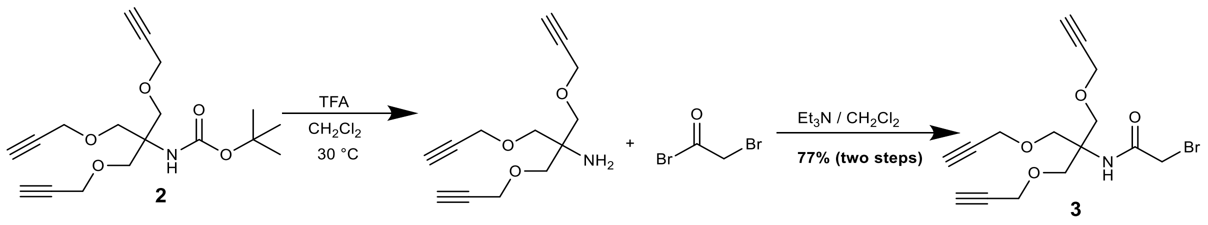

ZJ Chen, W Hu, M Wang, LS Wang, GF Su, JY Wang. *Carbohydrate Research*, **2016**, *429*, 81-86

N-(2-bromoacetyl)tris(propargyloxymethyl)aminomethane (3):

To a solution of compound **2** (250 mg, 0.75 mmol, 1 eq.) in CH_2Cl_2 (4 mL) was added trifluoroacetic acid (1 mL). The reaction was stirred at 30°C during 2 h. The mixture was then evaporated and toluene (2×10 mL) was used to co-evaporate TFA. The resulting crude product was dissolved with CH_2Cl_2 (10 mL) before adding triethylamine (0.22 mL, 1.5 mmol, 2eq.) and 2-bromoacetyl bromide (0.08 mL, 0.97 mmol, 1.3 eq.). The solution was stirred at room temperature during 18 h. The solution was then diluted with CH_2Cl_2 (50 mL), washed with brine (50 mL), HCl aqueous solution (30 mL, 2M), and brine (50 mL) successively. The organic layer was dried ($MgSO_4$), concentrated, and purified with silica gel column chromatograph (Cyclohexane:EtOAc = 3:1) to obtain compound **3** (204 mg, 77%) as a pale yellow oil.

R_f = 0.2 (Cyclohexane:EtOAc = 3:1)

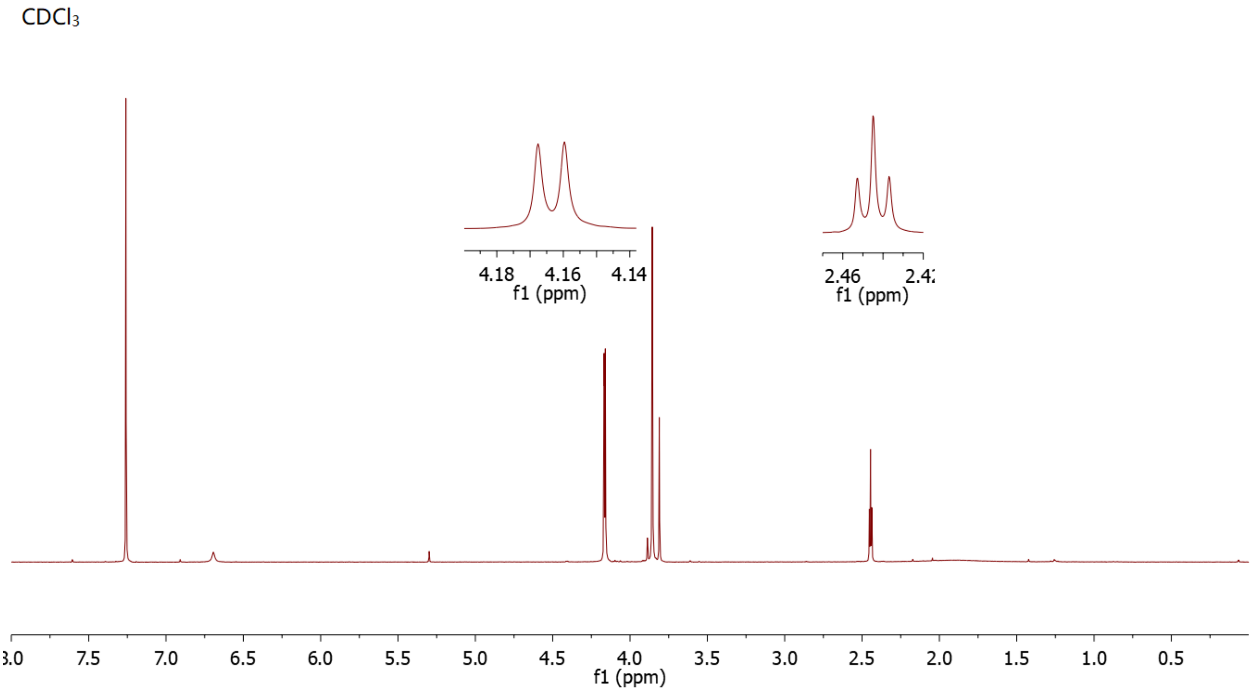

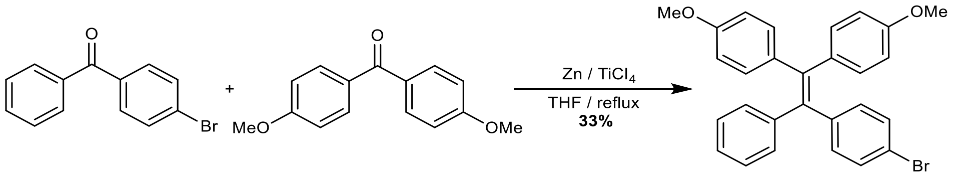

4

XL Cai, CJ Zhang, FTW Lim, SJ Chan, A Bandla, CK Chuan, B Liu, *et al. Small*, **2016**, *12*, 6576-6585

1-(4-bromophenyl)-2,2-(4-methoxyphenyl)-1-phenyl-ethene (4):

To a solution of 4-bromobenzophenone (2 g, 7.7 mmol, 1 eq.) and 4,4'-dimethoxybenzophenone (2.23 g, 9.2 mmol, 1.2 eq.) in dry THF (50 mL) was added Zn powder (4 g, 61.6 mmol, 8 eq.). Pure $TiCl_4$ (2 mL, 16.8 mmol, 4 eq.) was added to the reaction at 0°C during 30 min, and the mixture was heated to reflux during 20 h. The mixture was then quenched with H_2O (100 mL), evaporated, diluted with EtOAc (150 mL) and filtered over a Celite pad. The organic solution was washed with brine (3×50 mL), dried (Na_2SO_4), concentrated and purified with silica gel column chromatography (Cyclohexane:CH_2Cl_2 = 7:1) to obtain compound **4** (1.27 g, 33%) as a pale yellow amorphous solid.

R_f = 0.27 (Cyclohexane:CH_2Cl_2 = 3:1)

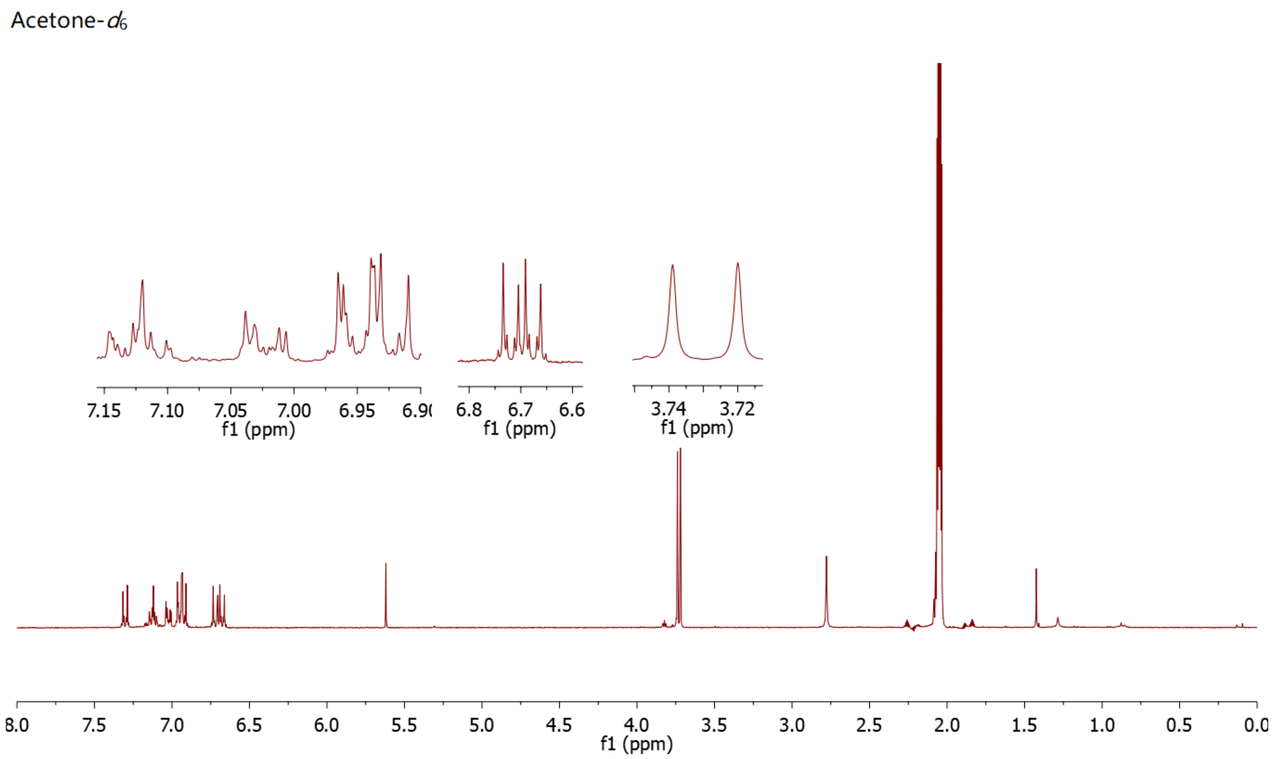

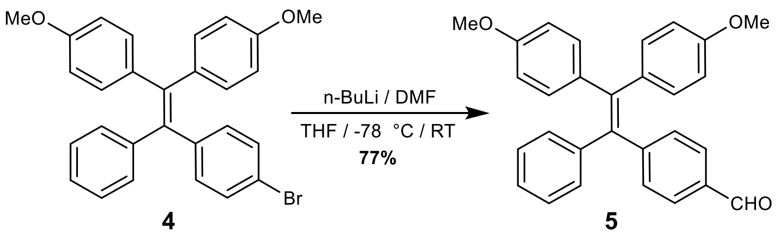

XL Cai, CJ Zhang, FTW Lim, SJ Chan, A Bandla, CK Chuan, B Liu, *et al. Small*, **2016**, *12*, 6576-6585

1-(4-formylphenyl)-2,2-(4-methoxyphenyl)-1-phenyl-ethene (5):

To the solution of compound **4** (1.265 g, 2.7 mmol, 1 eq.) in dry THF (10 mL) was added n-butyllithium (2.7 mL, 6.7 mmol, 2.5 eq., 2.5 M in hexane) at -78°C and continually stirred during 3 h. Then DMF (1.2 mL) was added and stirring was continued at RT during 18 h. The reaction was quenched by saturated NH_4Cl aqueous solution (15 mL), and diluted with EtOAc (100 mL). The organic layer was washed with brine (2×50 mL), dried ($MgSO_4$), concentrated and purified with silica gel column chromatography (Cyclohexane:CH_2Cl_2 = 1:1) to obtain compound **5** (873 mg, 77%) as a yellow amorphous solid.

R_f = 0.34 (Cyclohexane:CH_2Cl_2 = 1:1)

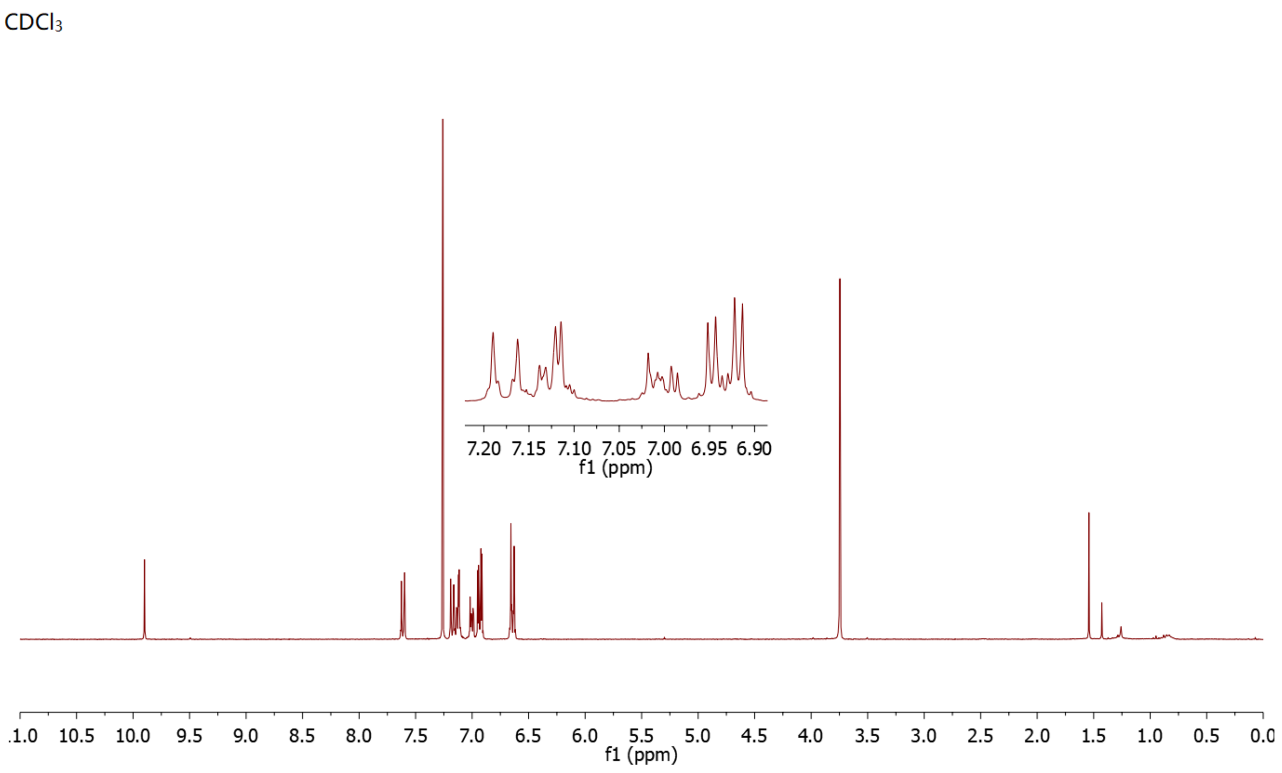

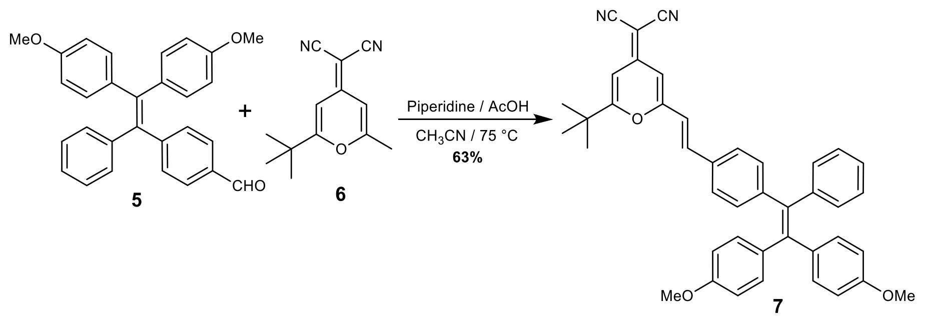

(*E*)-2-{2-{4-[2,2-di-(4-methoxyphenyl)-1-phenyl-1-vinyl]styryl}-6-(*tert*-butyl)-4*H*-pyran-4-ylidene}-propanedinitril (7):

To the solution of compound **5** (880 mg, 2.1 mmol, 1 eq.) and compound **6** (492 mg, 2.3 mmol, 1.1eq.) in CH_3CN (30 mL) was added piperidine (428mg, 5 mmol, 2.4 eq., 0.5 mL) and acetic acid (0.15 mL, 2.5 mmol, 1.2 eq.). The resulting solution was stirred at 83°C during 18 h. The mixture was evaporated and diluted with EtOAc (50 mL), washed with brine (2×50 mL). The organic layer was dried (Na_2SO_4), concentrated and purified with silica gel column chromatography (Cyclohexane:EtOAc = 8:1) to obtain compound **7** (819 mg, 63%) as a red amorphous solid.

R_f = 0.29 (Cyclohexane:CH_2Cl_2 = 5:1)

^{1}H NMR (300 MHz, $CDCl_3$): δ (ppm) 1.37 (s, 9H, $C(C\underline{H}_3)_3$), 3.75 (d, 6H, J = 3.3 Hz, 2xOCH_3), 6.55 (d, 1H, J = 2.1 Hz, H-3pyran), 6.62-6.68 (m, 6H, H-5pyran, 4xH-Ar, $\underline{H}$C=CH-Ph), 6.95 (dd, 4H, J = 7.8 Hz, 9 Hz, H-Ar), 7.00-7.14 (m, 7H, H-Ar), 7.26-7.37 (m, 3H, 2xH-Ar, HC=C$\underline{H}$-Ph).

^{13}C NMR (75 MHz, $CDCl_3$): δ (ppm) 28.1 ($C(\underline{C}H_3)_3$), 36.7 ($\underline{C}(CH_3)_3$), 55.11 (OCH_3), 55.14 (OCH_3), 59.5 ($\underline{C}(CN)_2$), 102.6 (C-3pyran), 107.0 (C-5pyran), 113.1 (CH-Ar), 113.2 (CH-Ar), 115.2 (2xCN), 117.7 (H$\underline{C}$=CH-Ph), 126.4 (CH-Ar), 127.3 (CH-Ar), 127.9 (CH-Ar), 131.4 (CH-Ar), 132.0 ($\underline{C}^{Ar}$-CH=CH-Pyran), 132.2 (CH-Ar), 132.61 (CH-Ar), 132.67 (CH-Ar), 135.9 (C^{Ar}-C=C), 136.0 (C^{Ar}-C=C), 137.7 (HC=C$\underline{H}$-Ph), 138.3 ($\underline{C}^{Ar}$-C=C), 141.6 ($\underline{C}^{Ar}$-C=C), 143.8 (C=C), 147.2 (C=C), 156.6 (C-6pyran), 158.3 ($\underline{C}^{Ar}$-OMe), 158.4 ($\underline{C}^{Ar}$-OMe), 159.0 (C-2pyran), 172.1 (C-4pyran).

HR-ESI-MS *m*/*z*: calcd. for $C_{42}H_{37}N_2O_3$ $[M+H]^+$ 617.2783, found 617.2799.

$CDCl_3$

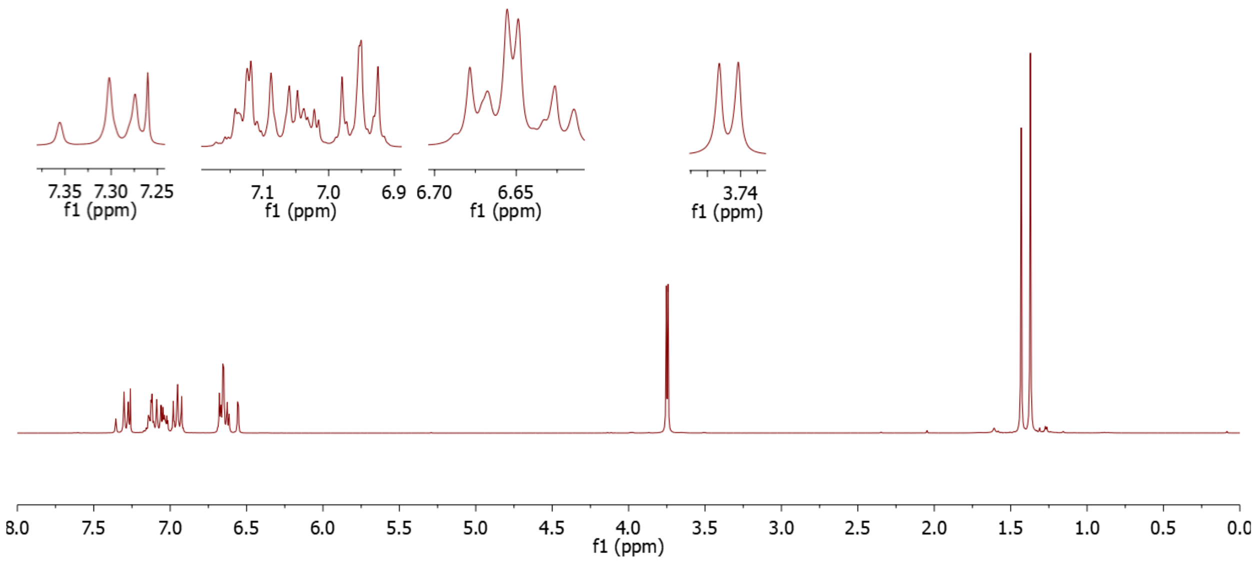

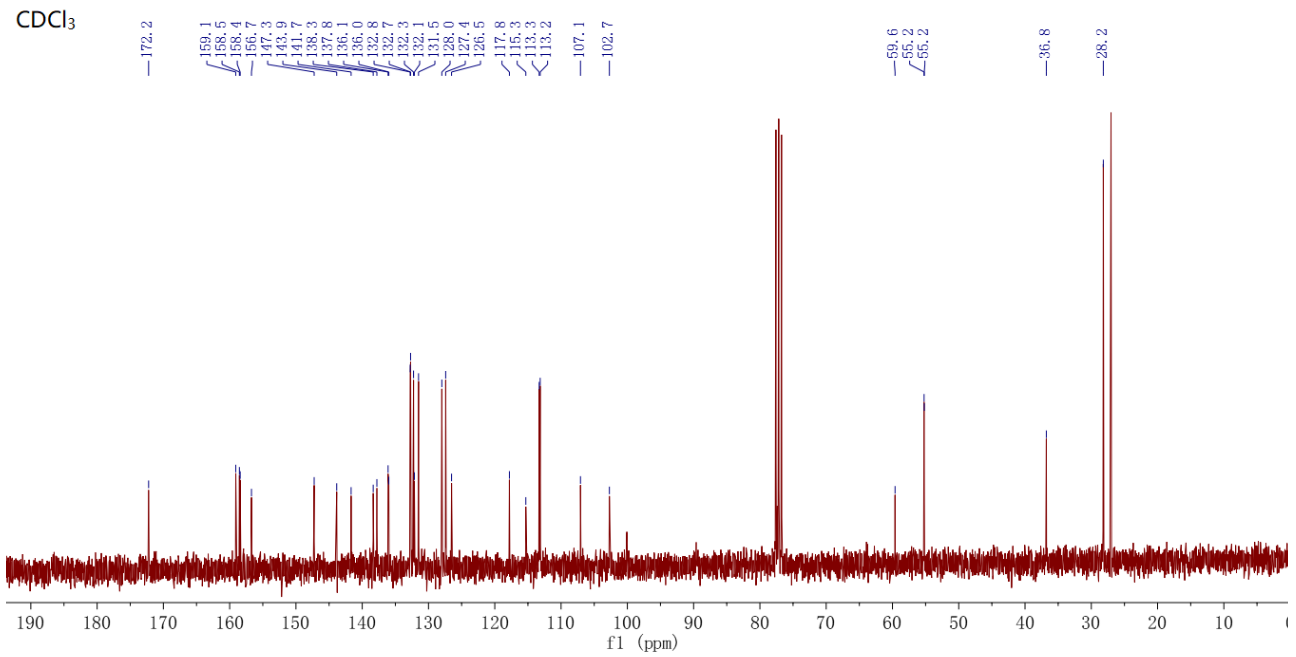

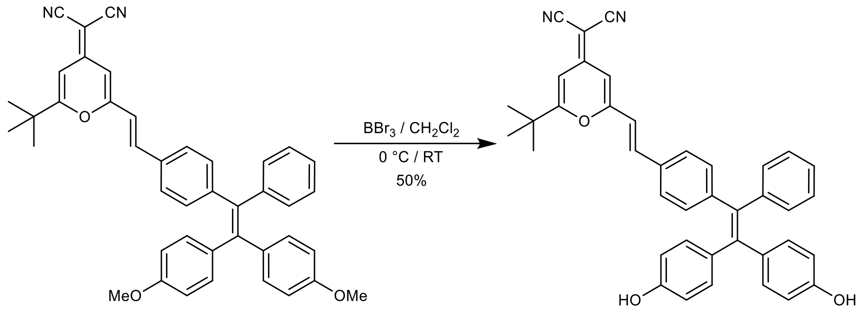

(*E*)-2-{2-{4-[2,2-di-(4-hydroxyphenyl)-1-phenyl-1-vinyl]styryl}-6-(*tert*-butyl)-4*H*-pyran-4-ylidene}-propanedinitrile (8):

To a solution of compound **7** (439 mg, 0.71 mmol, 1 eq.) in dry CH_2Cl_2 (10 mL) was added BBr_3 (1.8 mL, 1.78 mmol, 2.5 eq., 1 M in CH_2Cl_2) at 0°C. The resulting solution was stirred at 0°C, and then stirred at RT during 18 h. The solution was poured into ice-water (50 mL) and the solid compound was dissolved with EtOAc (100-150 mL). The aqueous layer was extracted with EtOAc (2×40 mL). The organic layers were combined, washed with brine (3×50 mL), dried ($MgSO_4$), concentrated, and purified with silica gel column chromatography (CH_2Cl_2:MeOH = 80:1) to obtain compound **8** (204 mg, 50%) as a red amorphous solid.

R_f = 0.26 (CH_2Cl_2:MeOH = 70:1)

^{1}H NMR (300 MHz, Acetone-d_6): δ (ppm) 1.42 (s, 9H, C(C<u>H</u>$_3$)$_3$), 6.56 (d, 1H, J = 2.1 Hz, H-3pyran), 6.61 (t, 4H, J = 9.0 Hz, H-Ar), 6.80 (d, 1H, J = 2.1 Hz, H-5pyran), 6.87 (t, 4H, J = 9.0 Hz, H-Ar), 7.03-7.15 (m, 7H, H-Ar), 7.21 (d, 1H, J = 16.2 Hz, <u>H</u>C=CH-Ph), 7.50 (d, 2H, J = 2.1 Hz, H-Ar), 7.59 (d, 1H, J = 19.2 Hz, HC=C<u>H</u>-Ph), 8.30 (d, 2H, J = 11.4 Hz, <u>H</u>O-Ph)

^{13}C NMR (75 MHz, Acetone-d_6): δ (ppm) 28.2 (C(<u>C</u>H$_3$)$_3$), 37.5 (<u>C</u>(CH$_3$)$_3$), 59.2 (<u>C</u>(CN)$_2$), 103.1 (C-3pyran), 107.8 (C-5pyran), 115.5 (CH-Ar), 115.7 (CH-Ar), 119.4 (H<u>C</u>=CH-Ph), 127.2 (2xCN), 128.5 (CH-Ar), 128.8

(CH-Ar), 129.6 (C^{Ar}-CH=CH-Pyran), 132.3 (CH-Ar), 132.9 (CH-Ar), 133.5 (CH-Ar), 133.6 (CH-Ar), 133.8 (CH-Ar), 135.9 (C^{Ar}-C=C), 136.0 (C^{Ar}-C=C), 138.5 (HC=CH-Ph), 138.8 (C^{Ar}-C=C), 143.2 (C^{Ar}-C=C), 145.2 (C=C), 148.1 (C=C), 157.3 (C^{Ar}-OH), 157.4 (C^{Ar}-OH), 157.7 (C-6pyran), 160.7 (C-2pyran), 173.7 (C-4pyran).

HR-ESI-MS *m*/*z*: calcd. for $C_{40}H_{33}N_2O_3$ $[M+H]^+$ 589.2462, found 589.2486.

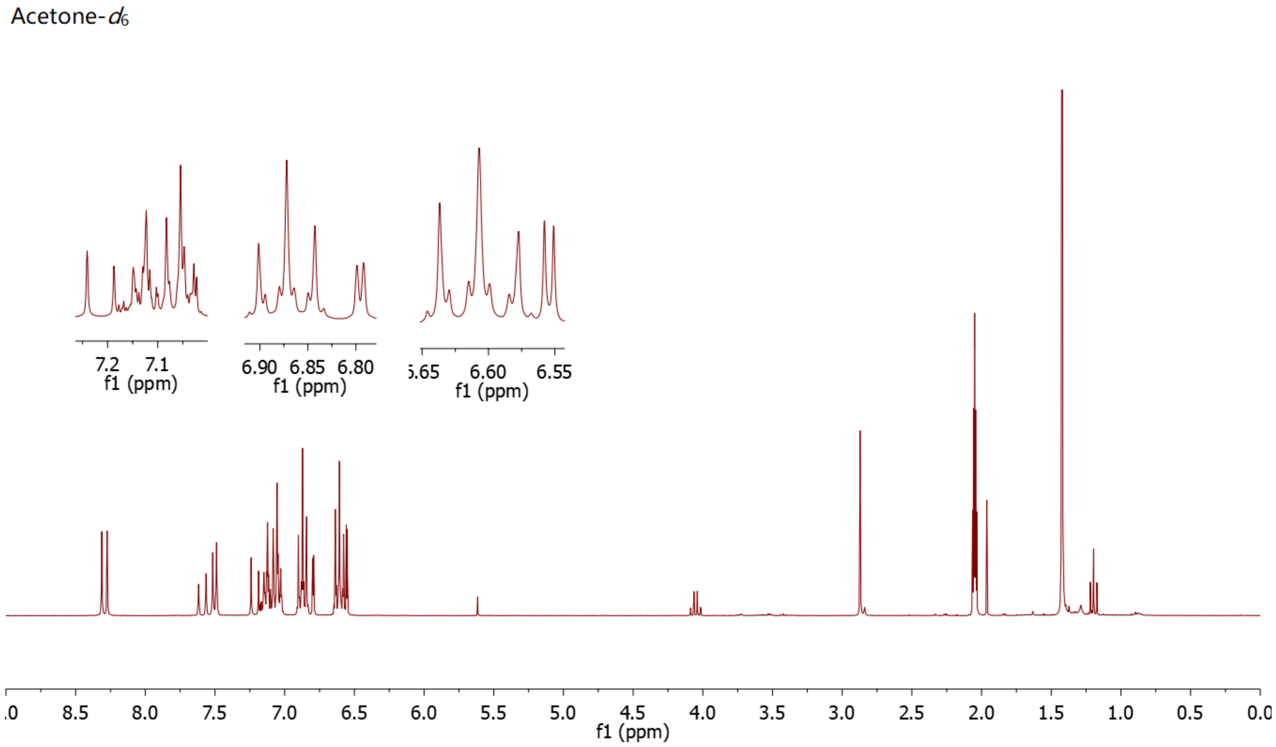

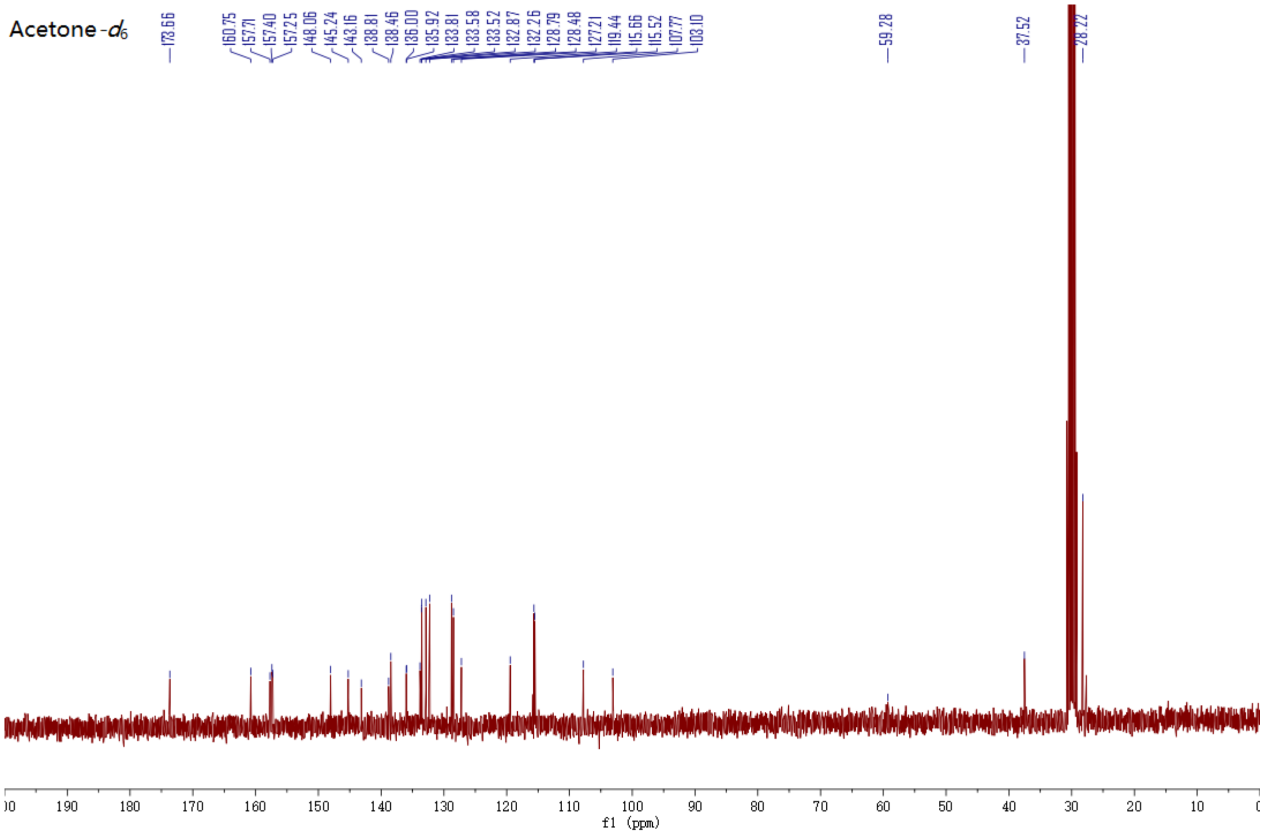

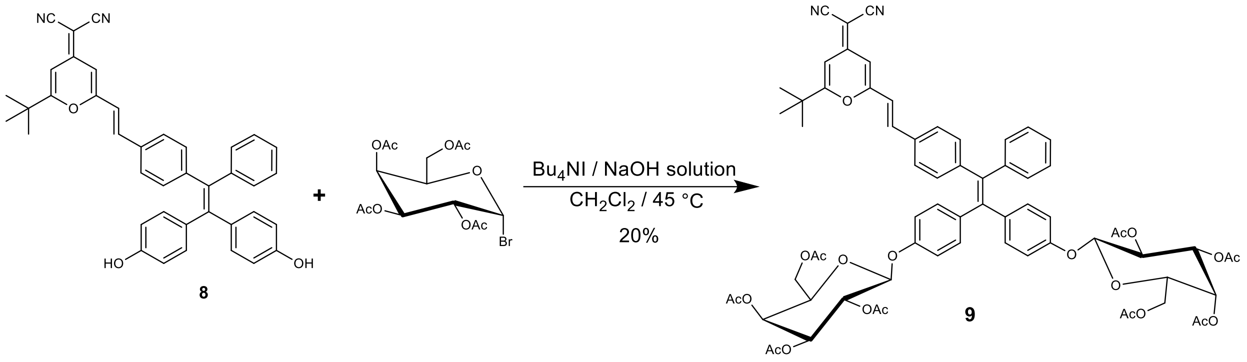

(*E*)-2-{2-{4-[2,2-di-(2,3,4,6-tetra-*O*-acetyl-β-D-galactopyranosyloxyphenyl)-1-phenyl-1-vinyl]styryl}-6-(*tert*-butyl)-4*H*-pyran-4-ylidene}-propanedinitrile (9):

Compound **8** (100 mg, 0.24 mmol, 1 eq.), 2,3,4,6-tetra-*O*-acetyl-1-bromo-α-D-galactose (400 mg, 0.97 mmol, 4 eq.), and tetrabutylammonium iodide (358 mg, 0.97 mmol, 4 eq.) were dissolved in CH_2Cl_2 (10 mL). 2M aqueous NaOH (6 mL) was added to the solution and vigorous stirring was continued at 45°C during 20 h. The mixture was diluted with CH_2Cl_2 (50 mL), and washed with 2 M aqueous HCl (2×30 mL) with a colour change from dark to orange. The aqueous layers were combined and extracted with CH_2Cl_2 (2×30 mL). The organic layers were combined, dried ($MgSO_4$), concentrated and purified with silica gel column chromatography (CH_2Cl_2:MeOH = 150:1) to obtain compound **9** (50 mg, 20%) as red amorphous solid.

R_f = 0.31 (CH_2Cl_2:MeOH = 100:1)

^{1}H NMR (300 MHz, $CDCl_3$): *δ* (ppm) 1.36 (s, 9H, $C(C\underline{H}_3)_3$), 2.00, 2.01, 2.05, 2.17 (4s, 4×6H, CH_3CO), 4.01 (t, 2H, *J* = 6.9 Hz, H-5), 4.07-4.19 (m, 2H, H-6'), 4.18-4.23 (m, 2H, H-6), 4.99 (d, 2H, *J* = 8.2 Hz, H-1), 5.08 (dd, 2H, *J* = 3.3 Hz, 10.2 Hz, H-3), 5.42-5.48 (m, 4H, H-2, H-4), 6.55 (d, 1H, *J* = 2.1 Hz, H-3pyran), 6.66 (d, 1H, *J* = 2.1 Hz, H-5pyran), 6.64 (d, 1H, *J* = 15.9 Hz, $\underline{H}$C=CH-Ph) , 6.75 (dd, 4H, *J* = 5.7 Hz, 8.7 Hz, H-Ar), 6.91-7.14 (m, 12H, H-Ar), 7.28-7.34 (m, 2H, H-Ar, HC=C$\underline{H}$-Ph).

^{13}C NMR (75 MHz, $CDCl_3$): *δ* (ppm) 20.6, 20.7, 20.75, 20.8 (4×$COC\underline{H}_3$), 28.2 ($C(\underline{C}H_3)_3$), 36.8 ($\underline{C}(CH_3)_3$), 59.6 ($\underline{C}(CN)_2$), 61.4 (C-6), 66.9 (C-4), 68.7 (C-2), 70.9 (C-5), 71.0 (C-3), 99.3 (C-1), 102.7 (C-3pyran), 107.2 (C-5pyran), 115.3 (H$\underline{C}$=CH-Ph), 116.1 (CH-Ar), 116.3 (CH-Ar), 118.1 (2xCN), 126.8 (CH-Ar), 127.4 (CH-Ar), 128.1 (CH-Ar), 131.3 (CH-Ar), 132.1 (CH-Ar), 132.5 ($\underline{C}^{Ar}$-CH=CH-Pyran), 132.6 (CH-Ar), 132.64 (CH-Ar), 132.67 (CH-Ar), 137.5 (HC=C$\underline{H}$-Ph), 138.3 (C^{Ar}-C=C), 138.5 (C^{Ar}-C=C), , 139.8 ($\underline{C}^{Ar}$-C=C), 140.5 ($\underline{C}^{Ar}$-C=C), 143.2 (C=C), 146.4 (C=C), 155.4 ($\underline{C}^{Ar}$-O), 155.6 ($\underline{C}^{Ar}$-O), 156.7 (C-6pyran), 158.9 (C-2pyran), 170.2, 170.3, 170.4, 170.43 (4×$\underline{C}OCH_3$), 172.3 (C-4pyran).

HR-ESI-MS *m*/*z*: calcd. for $C_{68}H_{68}N_2Na_2O_{21}$ $[M+2Na]^{2+}$ 647.2022, found 647.2049.

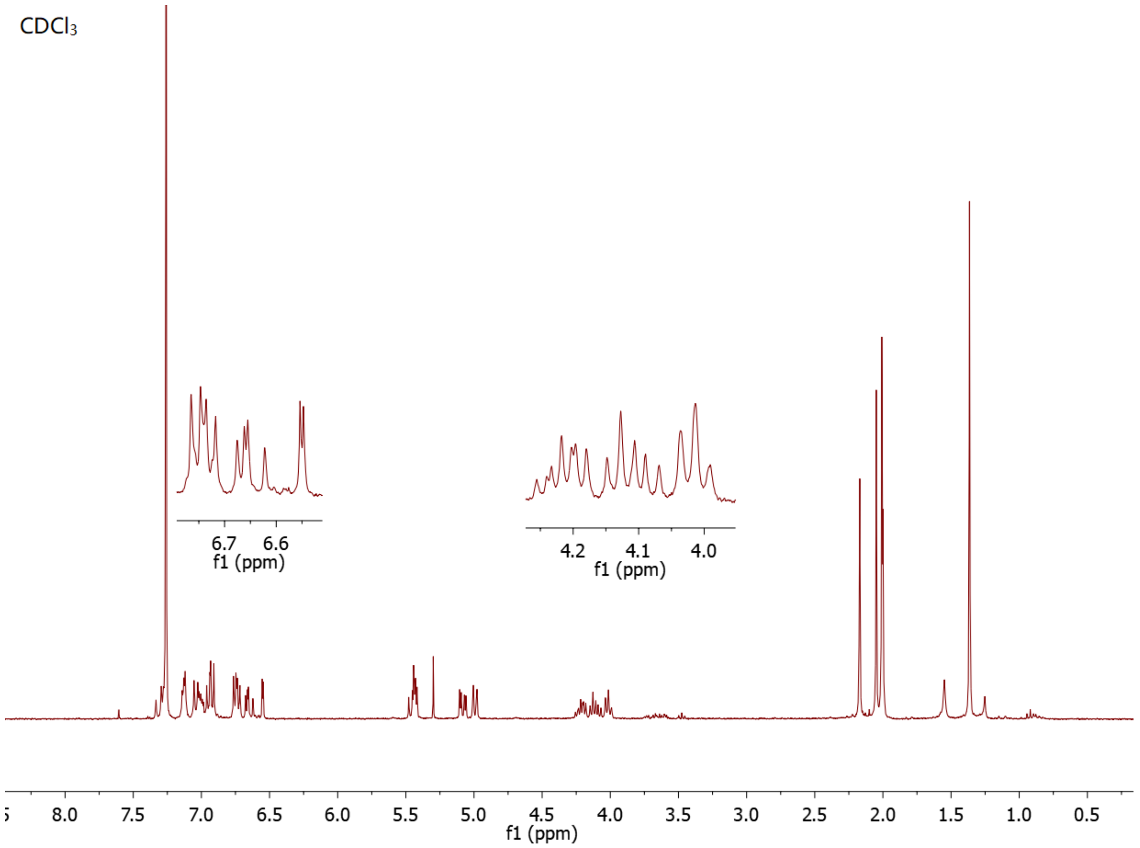

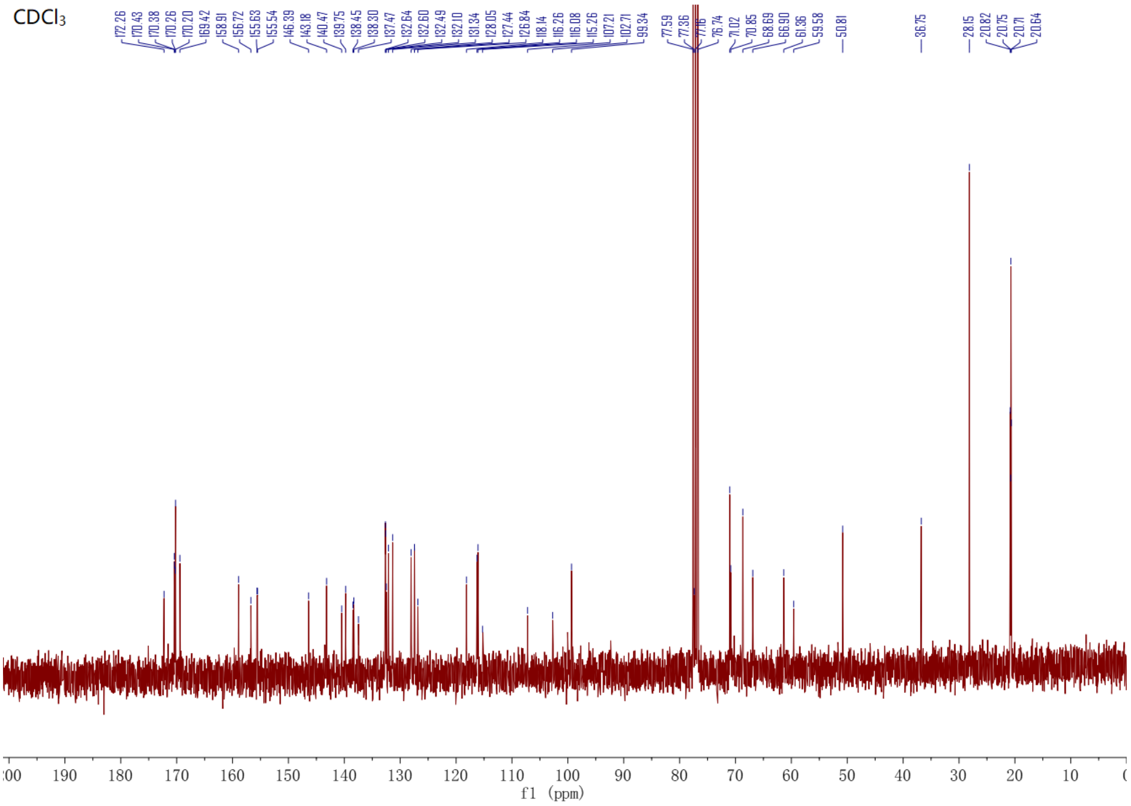

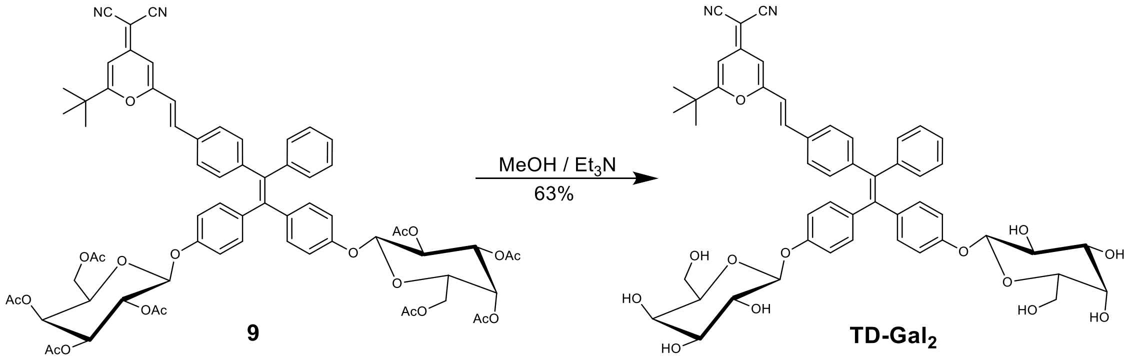

(*E*)-2-{2-[4-(2,2-di-β-D-galactopyranosyloxyphenyl-1-phenyl-1-vinyl)styryl]-6-(*tert*-butyl)-4*H*-pyran-4-ylidene}-propanedinitrile (TD-Gal$_2$) :

The solution of compound **9** (50 mg, 0.04 mmol, 1eq.) in MeOH/Et_3N (11 mL, 10:1, v/v) was stirred at 45°C during 20 h. The solution was concentrated then dissolved with MeOH (1 mL) and purified with C^{18} reverse phase column (H_2O:CH_3CN = 1:0 to 0:1) to afford compound **TD-Gal$_2$** (23 mg, 63%) as a red amorphous solid.

^{1}H NMR (300 MHz, CD_3OD / DMSO-d_6)): δ (ppm) 1.42 (s, 9H, C(C<u>H</u>$_3$)$_3$), 3.55 (dd, 2H, J = 2.4 Hz, 8,7 Hz, H-3), 3.66-3.76 (m, 8H, H-4, H-5, H-6, H-6'), 3,87-3.88 (m, 2H, H-2), 4.83 (dd, 2H, J = 2.4 Hz, 7.8 Hz, H-1), 6.58 (d, 1H, J = 2.1 Hz, H-3pyran), 6.85-7.19 (m, 17H, H-5pyran, 16×H-Ar), 7.45-7.51 (m, 3H, HC=C<u>H</u>-Ph, 2×H-Ar).

^{13}C NMR (75 MHz, CD_3OD / DMSO-d_6): δ (ppm) 28.4 (C($\underline{C}H_3$)$_3$), 37.8 ($\underline{C}$(CH_3)$_3$), 59.0 ($\underline{C}$(CN)$_2$), 62.3 (C-6), 70.1 (C-4), 72.2 (C-2), 74.8 (C-5), 76.9 (C-3), 101.3 (C-1), 102.6 (C-3pyran), 108.1 (C-5pyran), 109.2 (H$\underline{C}$=CH-Ph), 116.9 (CH-Ar), 117.0 (CH-Ar), 115.8 (2×CN), 127.75 (CH-Ar), 127.77 (CH-Ar), 128.8 (CH-Ar), 128.9 (CH-Ar), 129.2 (CH-Ar), 132.4 ($\underline{C}^{Ar}$-CH=CH-Pyran), 133.1 (CH-Ar), 133.5 (CH-Ar), 133.6 (CH-Ar), 138.7 (HC=C$\underline{H}$-Ph), 140.5 (C^{Ar}-C=C), 141.3(C^{Ar}-C=C), 14.2.5 ($\underline{C}^{Ar}$-C=C), 145.2 ($\underline{C}^{Ar}$-C=C), 147.8 (C=C), 148.9 (C=C), 158.1 ($\underline{C}^{Ar}$-O), 158.4 ($\underline{C}^{Ar}$-O), 160.8 (C-6pyran), 161.2 (C-2pyran), 174.2 (C-4pyran).

HR-ESI-MS *m*/*z*: calcd. for $C_{52}H_{53}N_2O_{13}$ $[M+H]^+$ 913.3542, found 913.3552.

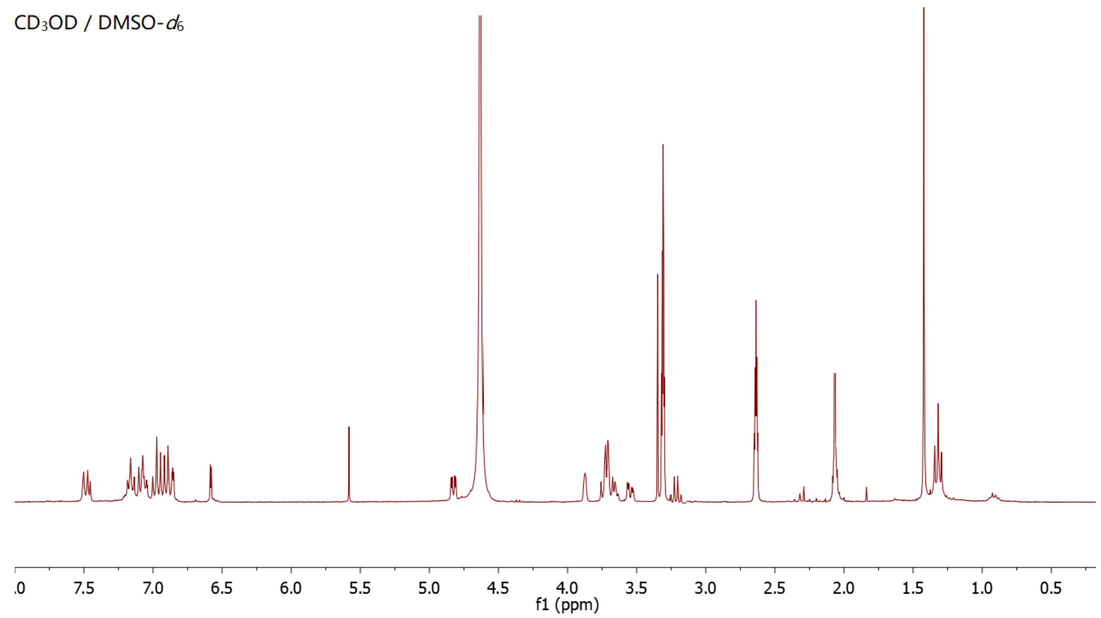

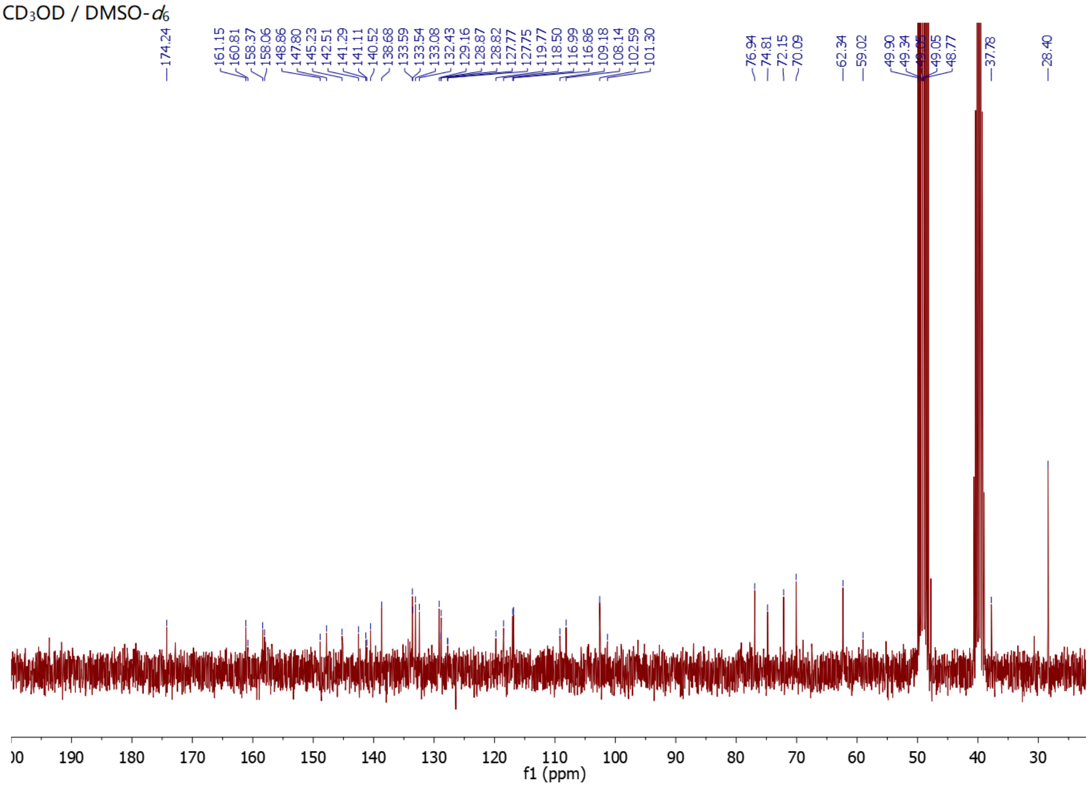

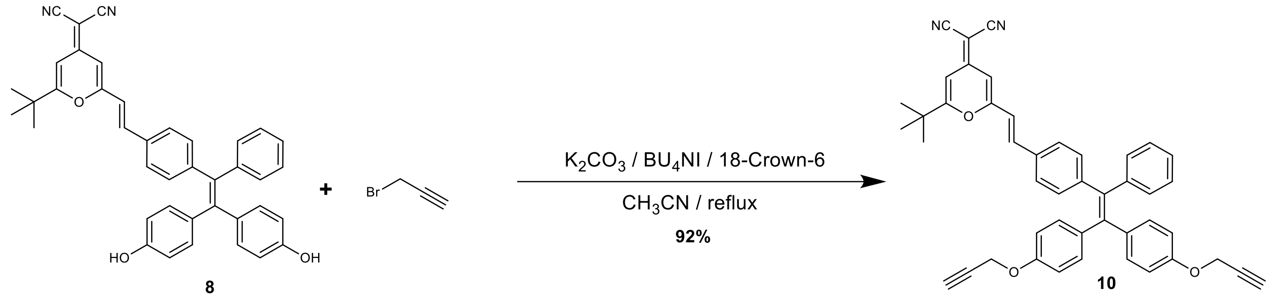

(*E*)-2-{2-[4-(2,2-di-(4-*O*-propargyloxyphenyl)-1-phenyl-1-vinyl)styryl]-6-(*tert*-butyl)-4*H*-pyran-4-ylidene}-propanedinitrile (10):

Propargyl bromide (0.7 mL, 6.6 mmol, 3 eq.) was added to a mixture of compound **8** (1.31 g, 2.2 mmol, 1eq.), K_2CO_3 (1.2 g, 8.8 mmol, 4 eq.), *n*-tetrabutylammonium iodide (2.4 g, 6.6 mmol, 3 eq.) and 18-crown-6 (290 mg, 1.1 mmol, 0.5 eq.) in acetonitrile (35 mL). The resulting mixture was stirred reflux under argon during 3 h. Then the mixture was quenched with water (50 mL), and extracted with EtOAc (2×50 mL). The organic layer was washed with brine (40 mL), HCl aqueous solution (40 mL, 1 M) and brine (40 mL), dried ($MgSO_4$), concentrated and purified with silica gel column chromatography (Cyclohexane:CH_2Cl_2 = 1:3 to pure CH_2Cl_2) to afford compound **10** (1.35g, 92%) as a red amorphous solid.

R_f = 0.31 (Cyclohexane:CH_2Cl_2 = 1:2)

^{1}H NMR (300 MHz, $CDCl_3$): δ (ppm) 1.36 (s, 9H, C(C$\underline{H}_3$)$_3$), 2.51 (dd, 2H, *J* = 2.4 Hz, 3.9 Hz, C≡C$\underline{H}$), 4.62 (t, 4H, *J* = 2.1 Hz, C$\underline{H}_2$-C≡CH), 6.55 (d, 1H, *J* = 2.1 Hz, H-3pyran), 6.64 (d, 1H, *J* = 15.9 Hz, $\underline{H}$C=CH-Ph), 6.65 (d, 1H, *J* = 2.1 Hz, H-5pyran), 6.72 (dd, 4H, *J* = 5.7 Hz, 9 Hz, H-Ar), 6.93-7.14 (m, 12H, H-Ar), 7.29-7.33 (m, 2H, H-Ar, HC=C$\underline{H}$-Ph).

^{13}C NMR (75 MHz, $CDCl_3$): δ (ppm) 28.2 (C($\underline{C}$H$_3$)$_3$), 36.8 ($\underline{C}$(CH$_3$)$_3$), 55.86 ($\underline{C}$H$_2$-C≡CH), 55.90 ($\underline{C}$H$_2$-C≡CH), 59.7 ($\underline{C}$(CN)$_2$), 75.6 (CH≡$\underline{C}$), 75.7 (CH≡$\underline{C}$), 78.56 ($\underline{C}$H≡C), 78.59 ($\underline{C}$H≡C), 102.7 (C-3pyran), 107.1 (C-5pyran), 114.1 (CH-Ar), 114.2 (CH-Ar), 115.3 (2xCN), 117.9(H$\underline{C}$=CH-Ph), 126.7 (CH-Ar), 127.4 (CH-Ar), 128.0 (CH-Ar), 131.5 (CH-Ar), 132.2 (CH-Ar), 132.3 ($\underline{C}^{Ar}$-CH=CH-Pyran), 132.69 (CH-Ar), 132.76 (CH-Ar), 136.8 (C^{Ar}-C=C), 136.9 (C^{Ar}-C=C), 137.7 (HC=C$\underline{H}$-Ph), 138.9 ($\underline{C}^{Ar}$-C=C), 141.3 ($\underline{C}^{Ar}$-C=C), 143.6 (C=C), 147.0 (C=C), 156.5 (C-6pyran), 156.6 ($\underline{C}^{Ar}$-O-propargyl), 156.7 ($\underline{C}^{Ar}$-O-propargyl), 159.0 (C-2pyran), 172.3 (C-4pyran).

HR-ESI-MS *m*/*z*: calcd. for $C_{46}H_{37}N_2O_3$ $[M+H]^+$ 665.2799, found 665.2722.

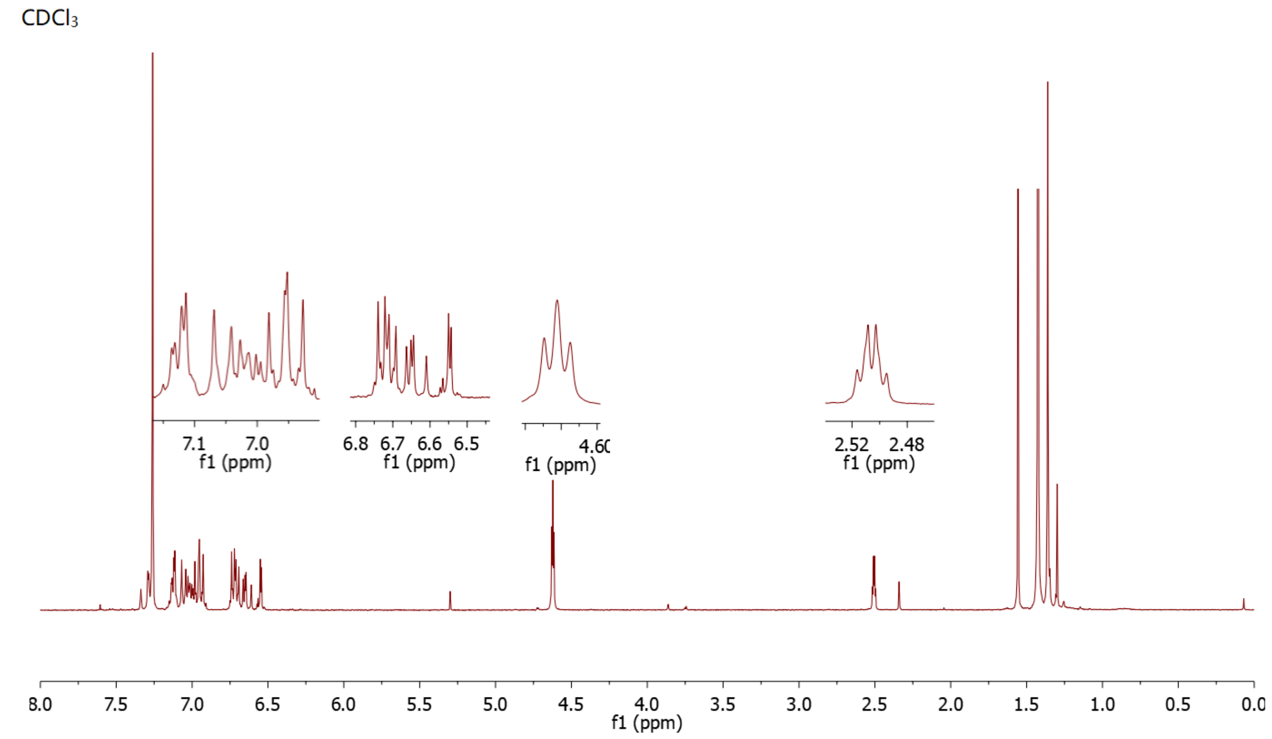
CDCl3
f1 (ppm)

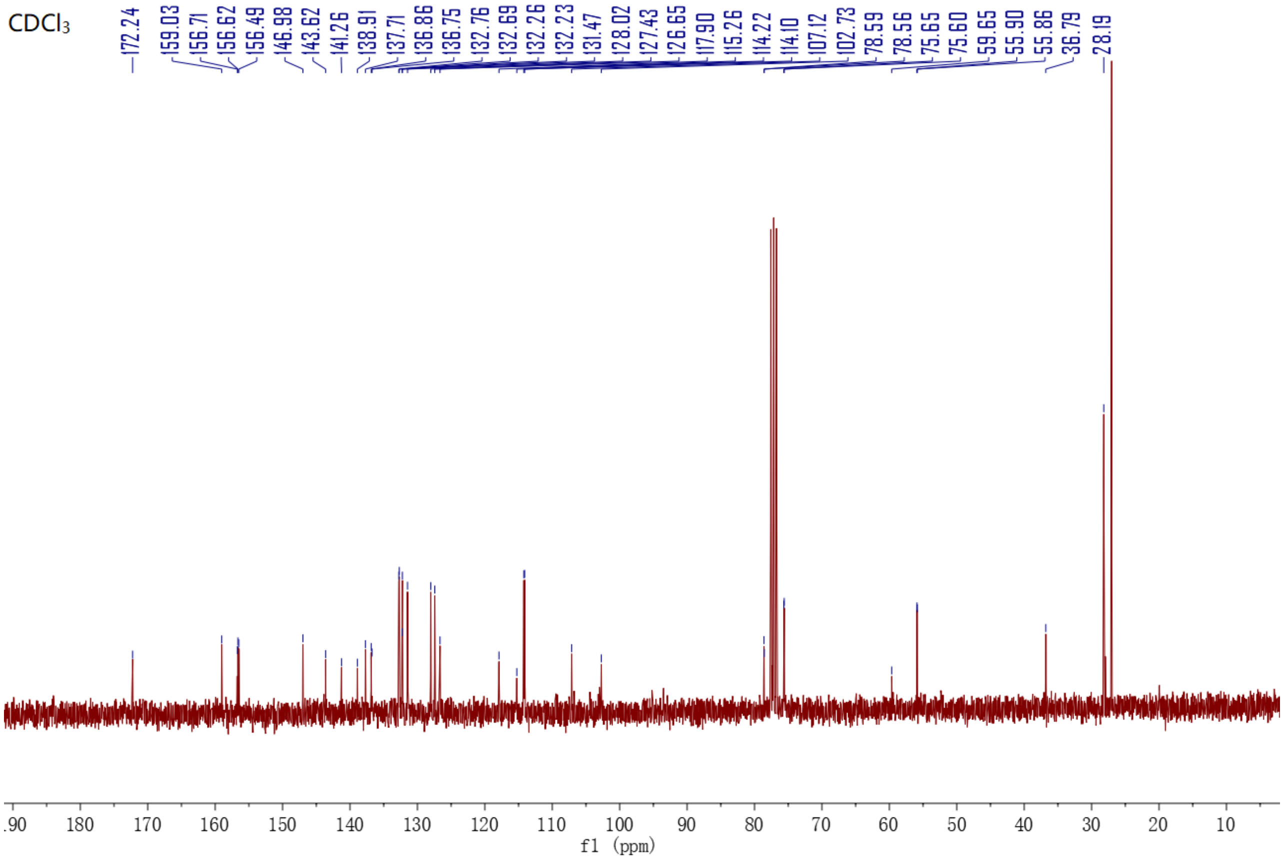
CDCl3
172.24
159.03
156.71
156.62
156.49
146.98
143.62
141.26
138.91
137.71
136.86
136.75
132.76
132.69
132.26
132.23
131.47
128.02
127.43
126.65
117.90
115.26
114.22
114.10
107.12
102.73
78.59
78.56
75.65
75.60
59.65
55.90
55.86
36.79
28.19
f1 (ppm)

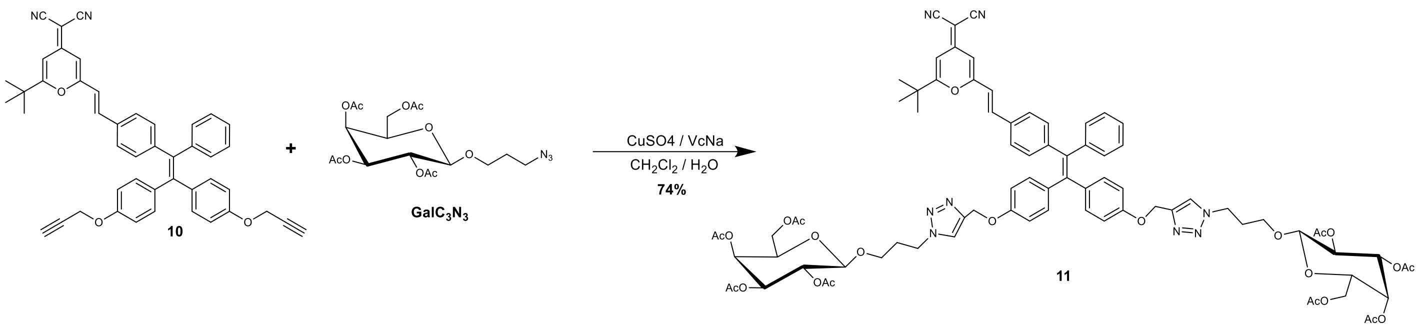

(*E*)-2-{2-[4-(2,2-di-(4-{1-[1-(2,3,4,6-tetra-*O*-acetyl-β-D-galactopyranosyloxy)-3-propyl]-1,2,3-triazol-4-yl-methoxy}phenyl)-1-phenyl-1-vinyl)styryl]-6-(*tert*-butyl)-4*H*-pyran-4-ylidene}-propanedinitrile (11):

To a solution of compound **10** (150 mg, 0.22 mmol, 1 eq.), compound **GalC$_3$N$_3$** (214 mg, 0.49 mmol, 2.2 eq.), and copper sulfate (80 mg, 0.44 mmol, 2 eq.) in CH_2Cl_2/H_2O (20 mL, 3:1, v/v) was added L-ascorbic acid sodium salt (178 mg, 0.9 mmol, 4 eq.). The resulting mixture was stirred vigorously at RT during 20 h. The reaction was diluted with CH_2Cl_2 (40 mL), H_2O (20 mL) and then separated. The aqueous layer was extracted with CH_2Cl_2 (2×30 mL). The combined organic layers were washed with saturated aqueous EDTA solution (2×40 mL), dried ($MgSO_4$), concentrated and purified with silica gel column chromatography (CH_2Cl_2:EtOAc= 2:1) to afford compound **11** (247 mg, 74%) as a red amorphous solid.

R_f = 0.2 (CH_2Cl_2:EtOAc= 2:1)

^{1}H NMR (300 MHz, $CDCl_3$): δ (ppm) 1.36 (s, 9H, C(C<u>H</u>$_3$)$_3$), 1.99, 2.02, 2.09, 2.16 (4s, 4×6H, CH_3CO), 2.13-2.24 (m, 4H, OCH$_2$C<u>H</u>$_2$CH$_2$-triaz), 3.46-3.56 (m, 2H, H-6’), 3.88-3.93 (m, 4H, H-5, H-6), 4.13-4.17 (m, 4H, OC<u>H</u>$_2$CH$_2$CH$_2$-triaz), 4.47 (d, 2H, J = 7.8 Hz, H-1), 4.41-4.51 (m, 4H, OCH$_2$CH$_2$C<u>H</u>$_2$-triaz), 5.02 (dd, 2H, J = 3.3 Hz, J = 10.5 Hz, H-3), 5.12 (d, 4H, J = 2.1 Hz, OCH$_2$-triaz), 5.18-5.29 (m, 2H, H-2), 5.40 (d, 2H, J = 3.3 Hz), 6.55 (d, 1H, J = 1.8 Hz, H-3pyran), 6.659 (d, 1H, J = 2.1 Hz, H-5pyran), 6.66 (d, 1H, J = 8.4 Hz, <u>H</u>C=CH-Ph), , 6.74 (dd, 4H, J = 6.6 Hz, 8.7 Hz, H-Ar), 6.91-7.14 (m, 12H, H-Ar), 7.30-7.35 (m, 2H, H-Ar, HC=C<u>H</u>-Ph), 7.62 (d, 2H, J = 7.8 Hz, H-triaz).

^{13}C NMR (75 MHz, $CDCl_3$): δ (ppm) 20.7, 20.78, 20.79, 21.0 (4×CO<u>C</u>H$_3$), 28.2 (C(<u>C</u>H$_3$)$_3$), 30.3 (OCH$_2$<u>C</u>H$_2$CH$_2$-triaz), 36.8 (<u>C</u>(CH$_3$)$_3$), 46.9 (OCH$_2$CH$_2$<u>C</u>H$_2$-triaz), 59.6 (<u>C</u>(CN)$_2$), 60.5 (O<u>C</u>H$_2$CH$_2$CH$_2$-triaz), 61.4 (<u>C</u>H$_2$-triaz), 65.9 (C-6), 67.1 (C-4), 68.9 (C-2), 78.88 (C-5), 78.89 (C-3), 101.3 (C-1), 102.7 (C-3pyran), 107.1 (C-5pyran), 113.96 (CH-Ar), 114.09 (CH-Ar), 115.3 (2xCN), 117.9(H<u>C</u>=CH-Ph), 123.3 (CH^{triaz}), 127.4 (CH-Ar), 128.0 (CH-Ar), 131.5 (CH-Ar), 132.2 (CH-Ar), 132.3 (<u>C</u>^{Ar}-CH=CH-Pyran), 132.75 (CH-Ar), 132.77 (CH-Ar), 132.8 (CH-Ar), 136.50 (C^{Ar}-C=C), 136.57 (C^{Ar}-C=C), 137.7 (HC=C<u>H</u>-Ph), 138.9 (<u>C</u>^{Ar}-C=C), 141.4 (<u>C</u>^{Ar}-C=C), 143.6 (C=C), 144.4 (C^{triaz}), 147.0 (C=C), 156.7 (C-6pyran), 157.2 (<u>C</u>^{Ar}-O), 157.4 (<u>C</u>^{Ar}-O), 159.1 (C-2pyran), 169.7, 170.2, 170.3, 170.5 (4×<u>C</u>OCH$_3$) 172.3 (C-4pyran).

HR-ESI-MS m/z: calcd. for $C_{88}H_{88}N_8O_{23}$ $[M+2H]^+$ 764.2976, found 764.2947.

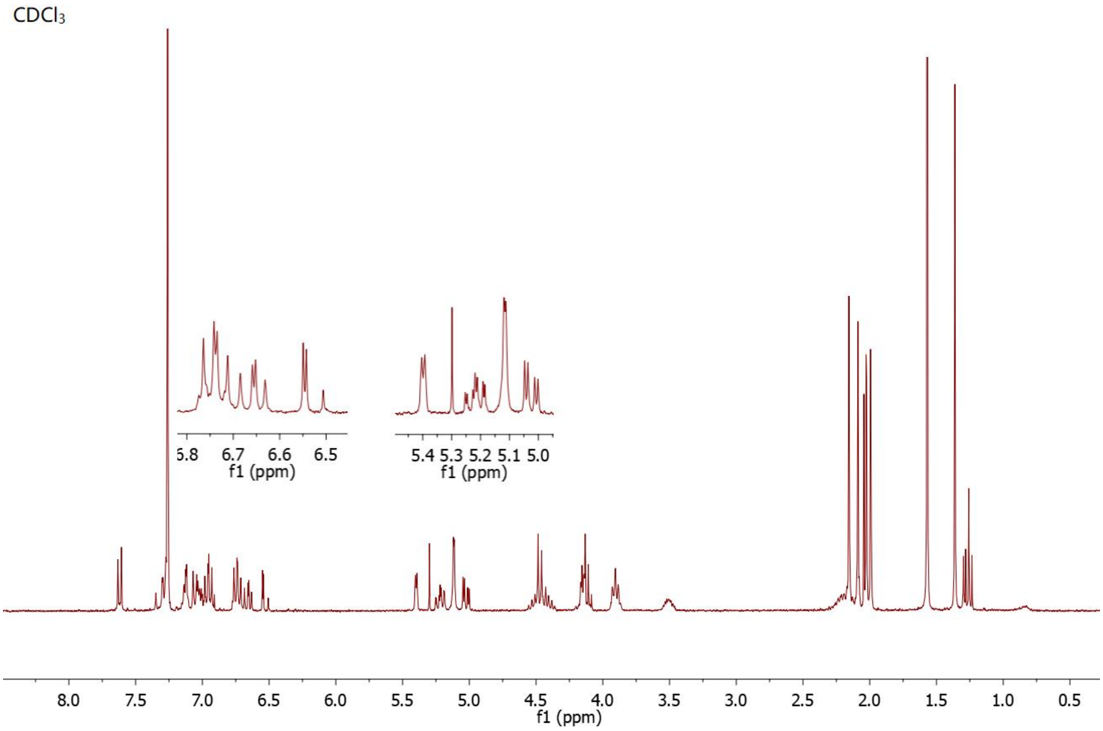
CDCl3
6.8 6.7 6.6 6.5
f1 (ppm)
5.4 5.3 5.2 5.1 5.0
f1 (ppm)
8.0 7.5 7.0 6.5 6.0 5.5 5.0 4.5 4.0 3.5 3.0 2.5 2.0 1.5 1.0 0.5
f1 (ppm)

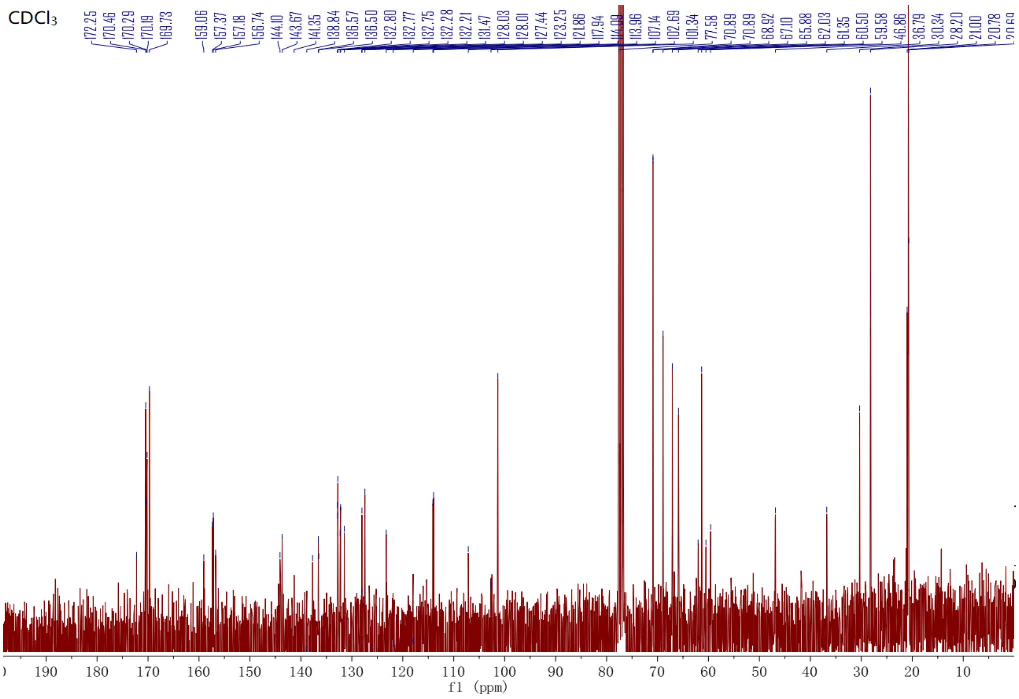
CDCl3
172.25 170.46 170.29 170.19 169.73 159.06 157.37 157.18 156.74 144.10 143.67 141.35 138.84 136.57 136.50 132.80 132.77 132.75 132.28 132.21 131.47 128.03 128.01 127.44 123.25 121.86 117.94 113.96 107.14 102.69 101.34 77.58 70.89 70.89 68.92 67.10 65.88 62.03 61.35 60.50 59.58 46.86 36.79 30.34 28.20 21.00 20.78
190 180 170 160 150 140 130 120 110 100 90 80 70 60 50 40 30 20 10
f1 (ppm)

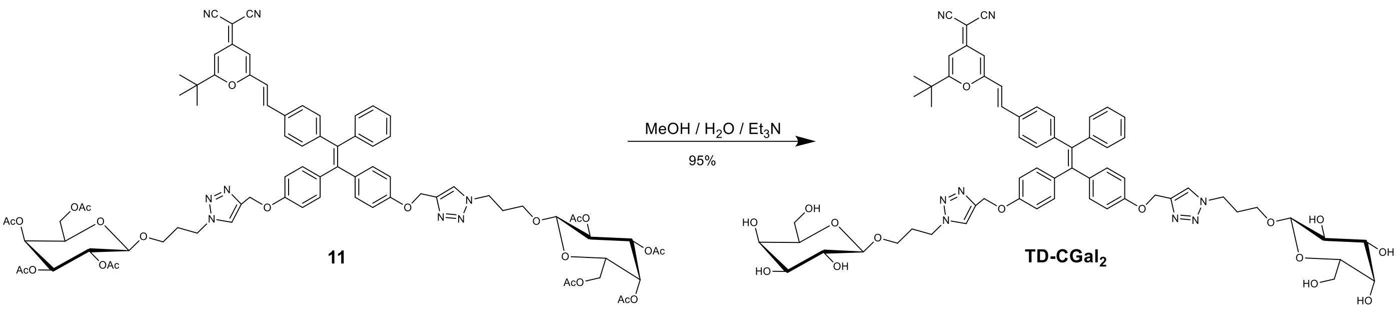

(*E*)-2-{2-(4-[2,2-di-(4-[1-(1-β-D-galactopyranosyloxy-3-propyl)-1,2,3-triazol-4-yl-methoxy]phenyl)-1-phenyl-1-vinyl]styryl)-6-(*tert*-butyl)-4*H*-pyran-4-ylidene}-propanedinitrile (TD-CGal$_2$):

The solution of compound **11** (115 mg, 0.075 mmol) in $MeOH/H_2O/Et_3N$ (17 mL, 12:3:2, v/v/v) was stirred at RT during 20 h. The solution was concentrated then diluted with MeOH (1 mL) and purified with C^{18} reverse phase column (H_2O:CH_3CN = 1:0 to 0:1) to afford compound **TD-CGal$_2$** (92 mg, 95%) as a red amorphous solid.

^{1}H NMR (300 MHz, CD_3OD): δ (ppm) 1.40 (s, 9H, $C(C\underline{H}_3)_3$), 2.17-2.21 (m, 4H, $OCH_2C\underline{H}_2CH_2$-triaz), 3.34-3.58 (m, 8H, H-2, H-3, H-5, H-6’), 3.72-3.76 (m, 4H, $OC\underline{H}_2CH_2CH_2$-triaz), 3.82-3.91 (m, 4H, H-4, H-6), 4.20 (dd, 2H, J = 2.1 Hz, 7.2 Hz, H-1), 4.56-4.61 (m, 4H, $OCH_2CH_2C\underline{H}_2$-triaz), 5.09 (s, 4H, OCH_2-triaz), 6.58 (d, 1H, J = 2.1 Hz, H-3pyran), 6.74-6.80 (m, 5H, H-5pyran, 4×H-Ar), 6.91-7.12 (m, 12H, $\underline{H}$C=CH-Ph, 11×H-Ar), 7.40-7.47 (m, HC=C$\underline{H}$-Ph, 2×H-Ar), 8.12 (d, 2H, J = 2.1 Hz, H-triaz).

^{13}C NMR (75 MHz, CD_3OD): δ (ppm) 28.3 ($C(\underline{C}H_3)_3$), 31.5 ($OCH_2\underline{C}H_2CH_2$-triaz), 37.7 ($\underline{C}(CH_3)_3$), 48.3 ($OCH_2CH_2\underline{C}H_2$-triaz), 59.1 ($\underline{C}(CN)_2$), 60.8 ($O\underline{C}H_2CH_2CH_2$-triaz), 62.5 ($\underline{C}H_2$-triaz), 66.7 (C-6), 70.3 (C-4), 72.5 (C-2), 74.9 (C-5), 76.7 (C-3), 102.9 (C-3pyran), 105.0 (C-1), 108.1 (C-5pyran), 115.1 (CH-Ar), 115.3 (CH-Ar), 118.7(H$\underline{C}$=CH-Ph), 120.2 (2×CN), 126.1 (CH^{triaz}), 128.67 (CH-Ar), 127.70 (CH-Ar), 129.0 (CH-Ar), 132.5 ($\underline{C}^{Ar}$-CH=CH-Pyran), 133.1 (CH-Ar), 132.7 (CH-Ar), 132.8 (CH-Ar) , 134.1 (CH-Ar), 137.76 (C^{Ar}-C=C), 137.8 (C^{Ar}-C=C), 138.8 (HC=C$\underline{H}$-Ph), 140.3 ($\underline{C}^{Ar}$-C=C), 141.4 (C^{triaz}),144.5 ($\underline{C}^{Ar}$-C=C), 143.6 (C=C), 145.2 (C^{triaz}), 152.0 (C=C), 155.0 (C-6pyran), 158.6 ($\underline{C}^{Ar}$-O), 158.7 ($\underline{C}^{Ar}$-O), 159.3 (C-2pyran), 172.2 (C-4pyran).

HR-ESI-MS *m*/*z*: calcd. for $C_{64}H_{72}N_8O_{15}$ $[M+2H]^{2+}$ 596.2553, found 596.2537.

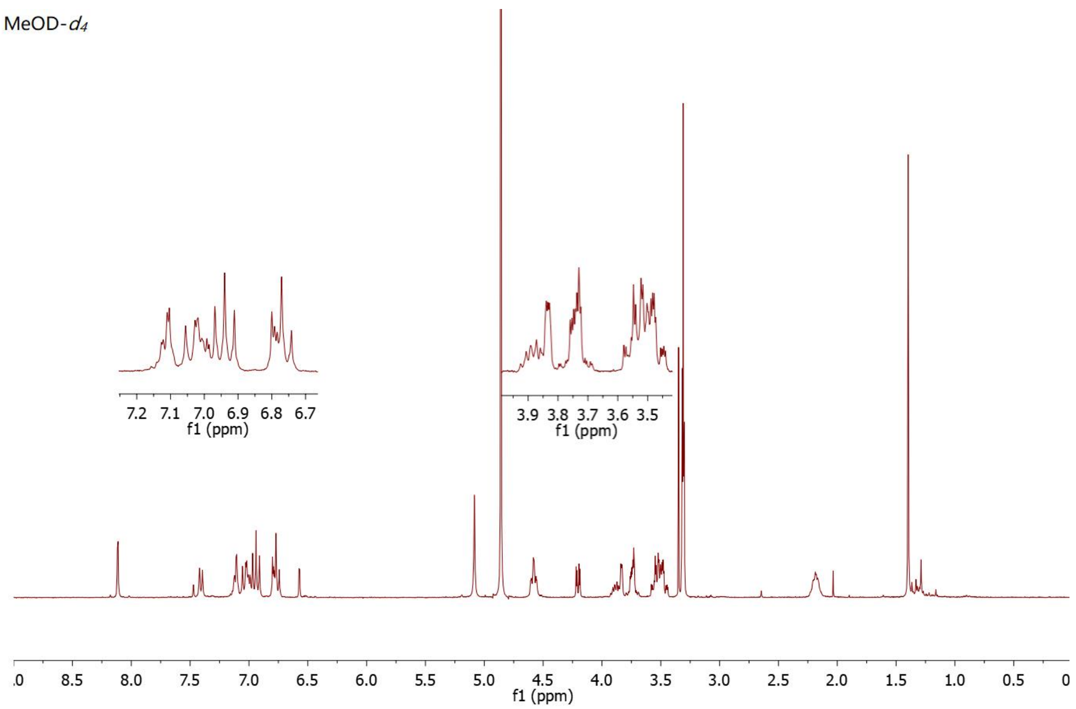
MeOD-d_4
7.2 7.1 7.0 6.9 6.8 6.7
f1 (ppm)
3.9 3.8 3.7 3.6 3.5
f1 (ppm)
.0 8.5 8.0 7.5 7.0 6.5 6.0 5.5 5.0 4.5 4.0 3.5 3.0 2.5 2.0 1.5 1.0 0.5 0
f1 (ppm)

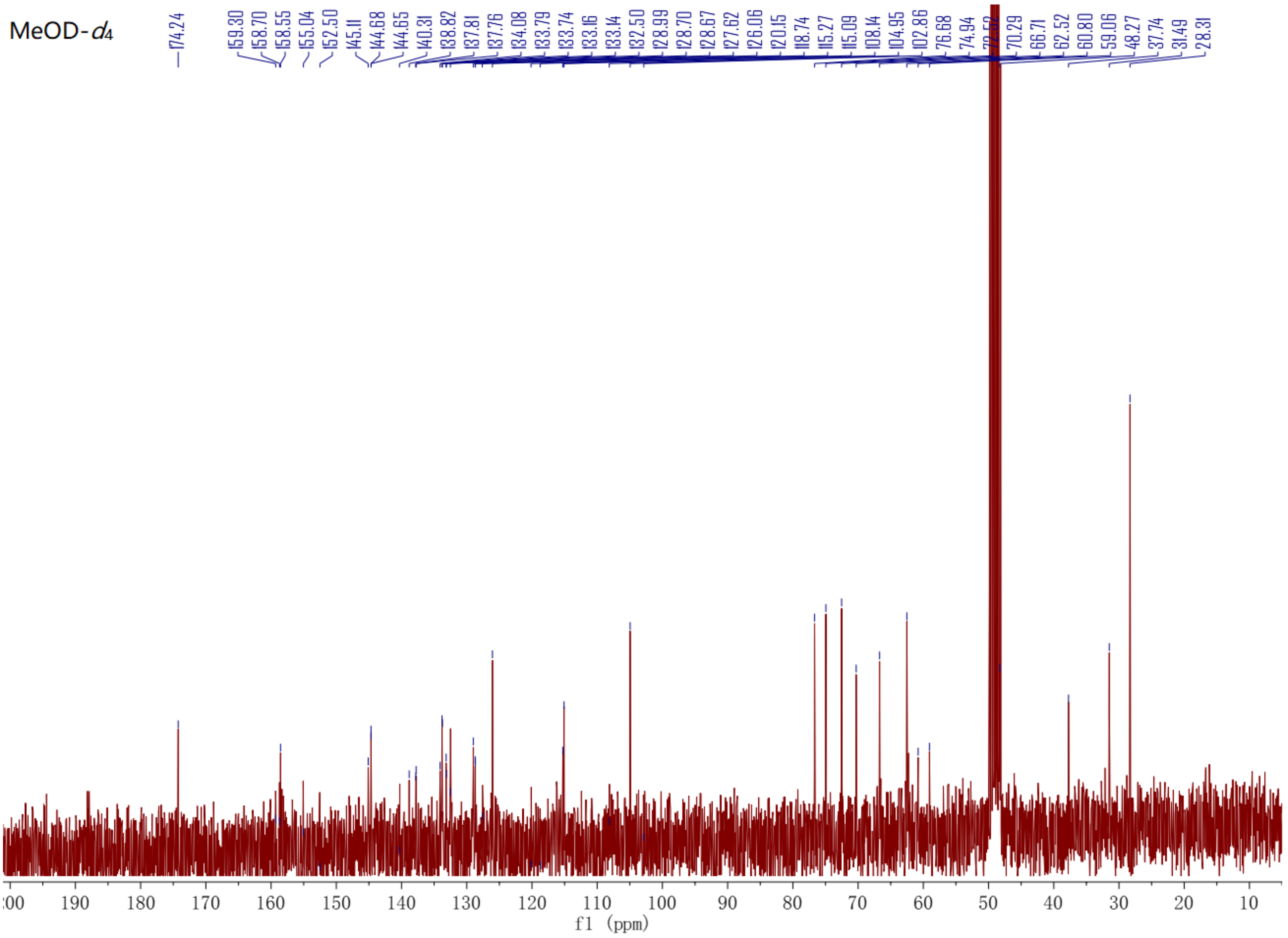
MeOD-d_4
174.24
159.30 158.70 158.55 155.04 152.50 145.11 144.68 144.65 140.31 138.82 137.81 137.76 134.08 133.79 133.74 133.16 133.14 132.50 128.99 128.70 128.67 127.62 126.06 120.15 118.74 115.27 115.09 108.14 104.95 102.86 76.68 74.94
70.29 66.71 62.52 60.80 59.06 48.27 37.74 31.49 28.31
00 190 180 170 160 150 140 130 120 110 100 90 80 70 60 50 40 30 20 10
f1 (ppm)

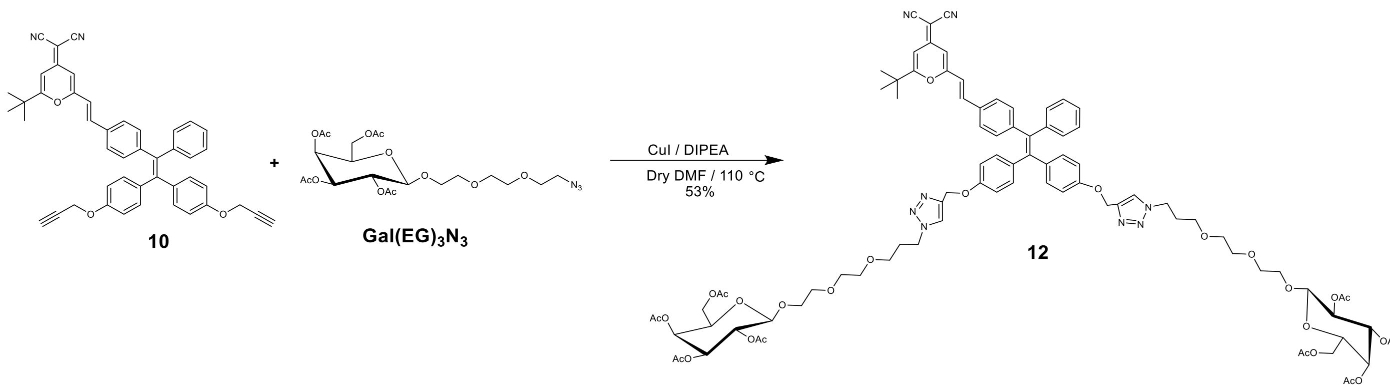

(*E*)-2-{2-[4-(2,2-di-(4-{1-[1-(2,3,4,6-tetra-*O*-acetyl-β-D-glucopyranosyloxy)-3,6-dioxaoct-8-yl]-1,2,3-triazol-4-yl-methoxy}phenyl)-1-phenyl-1-vinyl)styryl]-6-(*tert*-butyl)-4*H*-pyran-4-ylidene}-propanedinitrile (12):

To a solution of compound **10** (140 mg, 0.21 mmol, 1 eq.), compound **Gal(EG)$_3$N$_3$** (242 mg, 0.48 mmol, 2.3 eq.) and copper iodide (CuI, 10 mg, 0.05 mmol, 0.2 eq.) in dry DMF (12 mL) was added N,N-diisopropylethyl-amine (130 mg , 1.05 mmol, 5 eq., 0.2 mL). The resulting mixture was stirred under microwaves activation at 110°C during 30 min. Then the mixture was diluted with EtOAc (50 mL), washed with saturated EDTA aqueous solution (2×50 mL) and brine (50 mL). The organic layer was combined, dried ($MgSO_4$), concentrated and purified with silica gel column chromatography (CH_2Cl:MeOH = 50:1) to obtain compound **12** (185 mg, 53%) as red amorphous solid.

R_f = 0.20 (CH_2Cl_2:MeOH = 40:1)

^{1}H NMR (300 MHz, $CDCl_3$): *δ* (ppm) 1.35 (s, 9H, C(C<u>H</u>$_3$)$_3$), 1.96, 2.01, 2.02, 2.12 (4s, 4×6H, CH_3CO), 3.56-3.63 (m, 12H, OCH_2CH_2O), 3.66-3.72 (m, 2H, Gal-OC<u>H</u>$_2$), 3.86-3.96 (m, 8H, OC<u>H</u>$_2$CH$_2$N, H-5, Gal-OC<u>H</u>$_2$), 4.09-4.15 (m, 4H, H-6, H-6'), 4.51-4.58 (m, 6H, H-1, OCH$_2$C<u>H</u>$_2$N), 5.00 (dd, 2H, *J* = 3.0 Hz, 9.0 Hz, H-3), 5.11 (s, 4H, OC<u>H</u>$_2$-triaz), 5.15-5.22 (m, 2H, H-2), 5.37 (d, 2H, *J* = 3.3 Hz, H-4), 6.53 (d, 1H, *J* = 1.8 Hz, H-3pyran), 6.65 (d, 1H, *J* = 1.8 Hz, H-5pyran), 6.66 (d, 1H, *J* = 15.9 Hz, <u>H</u>C=CH-Ph), 6.73 (dd, 4H, *J* = 6.9 Hz, 9 Hz, H-Ar), 6.91-7.13 (m, 12H, H-Ar), 7.26-7.34 (m, 2H, H-Ar, HC=C<u>H</u>-Ph), 7.78 (d, 2H, *J* = 5.4 Hz, H-triaz).

^{13}C NMR (75 MHz, $CDCl_3$): *δ* (ppm) 20.7, 20.76, 20.79, 20.8 (4×CO<u>C</u>H$_3$), 28.2 (C(<u>C</u>H$_3$)$_3$), 36.8 (<u>C</u>(CH$_3$)$_3$), 50.4 (OCH$_2$<u>C</u>H$_2$N), 59.5 (<u>C</u>(CN)$_2$), 61.3 (O<u>C</u>H$_2$-triaz), 61.9 (C-6), 67.1 (C-4), 68.9 (C-2), 69.3 (Gal-O<u>C</u>H$_2$), 69.5 (O<u>C</u>H$_2$CH$_2$N), 70.3 (C-5), 70.65 (OCH_2), 70.70 (OCH_2), 70.8 (OCH_2), 70.9 (C-3), 101.4 (C-1), 102.7 (C-3pyran), 107.1 (C-5pyran), 113.96 (CH-Ar), 114.09 (CH-Ar), 115.3 (2xCN), 117.9 (H<u>C</u>=CH-Ph), 124.1 (CH^{triaz}), 127.4 (CH-Ar), 128.0 (CH-Ar), 131.5 (CH-Ar), 132.2 (CH-Ar), 132.24(<u>C</u>^{Ar}-CH=CH-Pyran), 132.71 (CH-Ar), 132.74 (CH-Ar), 132.8 (CH-Ar), 136.4 (C^{Ar}-C=C), 136.5 (C^{Ar}-C=C), 137.7 (HC=C<u>H</u>-Ph), 138.8 (<u>C</u>^{Ar}-C=C), 141.3 (<u>C</u>^{Ar}-C=C), 143.7 (C=C), 147.0 (C=C), 156.7 (C-6pyran), 157.2 (<u>C</u>^{Ar}-O), 157.4 (<u>C</u>^{Ar}-O), 159.1 (C-2pyran), 169.5, 170.2, 170.3, 170.5 (4×<u>C</u>OCH$_3$), 172.3 (C-4pyran).

HR-ESI-MS *m*/*z*: calcd. for $C_{86}H_{100}N_8O_{27}$ $[M+2H]^{2+}$ 838.3343, found 838.3352.

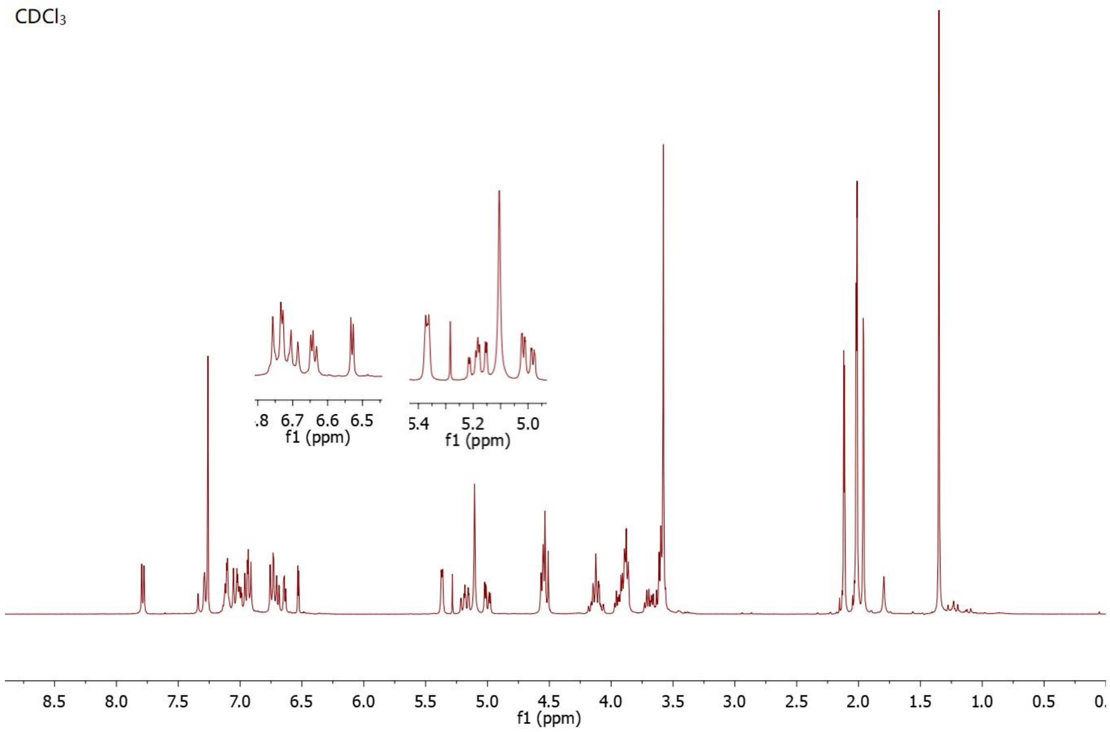
CDCl3
.8 6.7 6.6 6.5
f1 (ppm)
5.4 5.2 5.0
f1 (ppm)
8.5 8.0 7.5 7.0 6.5 6.0 5.5 5.0 4.5 4.0 3.5 3.0 2.5 2.0 1.5 1.0 0.5 0.
f1 (ppm)

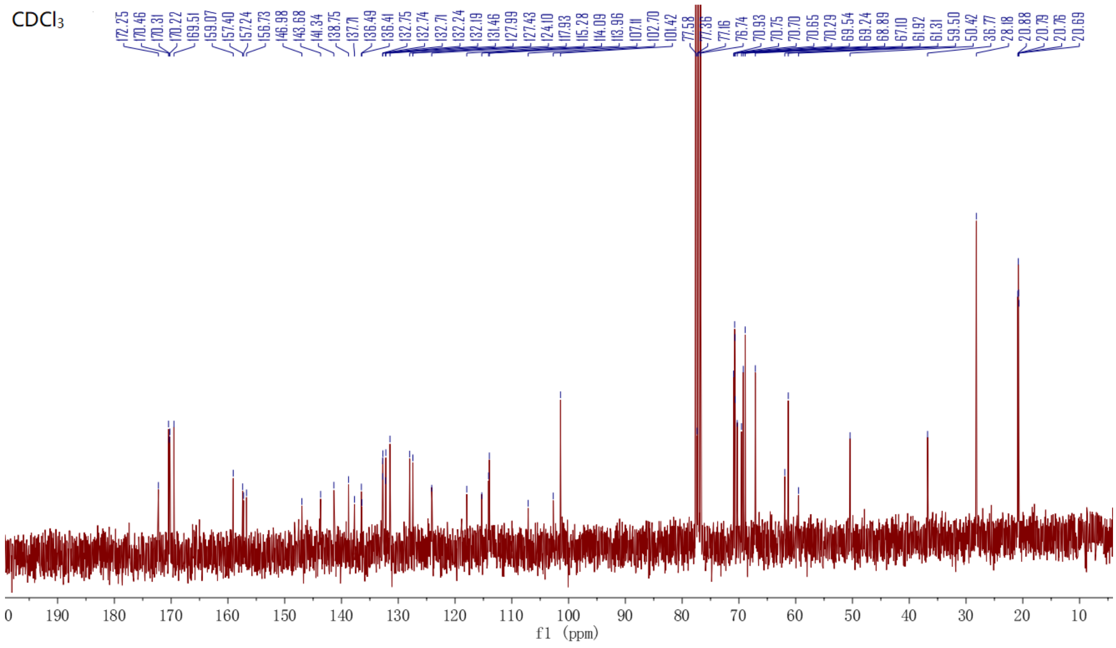
CDCl3
172.25 170.46 170.31 170.22 169.51 159.07 157.40 157.24 156.73 146.98 143.68 141.34 138.75 137.71 136.49 136.41 132.75 132.74 132.71 132.24 132.19 131.46 127.99 127.43 124.10 117.93 115.28 114.09 113.96 107.11 102.70 101.42 77.58 77.36 77.16 76.74 70.93 70.75 70.70 70.65 70.29 69.54 69.24 68.89 67.10 61.92 61.31 59.50 50.42 36.77 28.18 20.88 20.79 20.76 20.69
0 190 180 170 160 150 140 130 120 110 100 90 80 70 60 50 40 30 20 10
f1 (ppm)

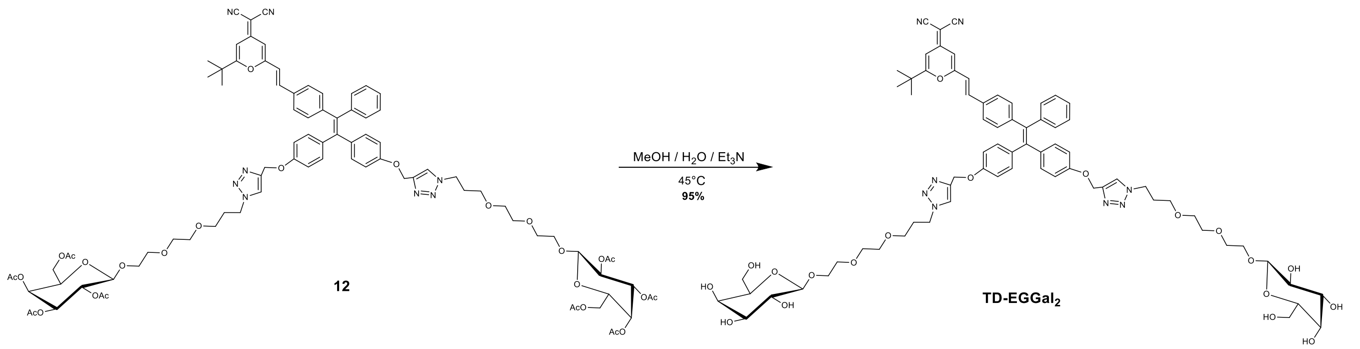

(*E*)-2-{2-[4-(2,2-di-(4-{1-[1-(β-D-glucopyranosyloxy)-3,6-dioxaoct-8-yl]-1,2,3-triazol-4-yl-methoxy}phenyl)-1-phenyl-1-vinyl)styryl]-6-(*tert*-butyl)-4*H*-pyran-4-ylidene}-propanedinitrile (TD-EGGal$_2$):

The solution of compound **12** (120 mg, 0.072 mmol) in mixed solution of MeOH (8 mL), H_2O (2 mL), and Et_3N (2 mL) was stirred at 45°C during 20 h. The solution was evaporated, then dissolved with MeOH (1 mL) and purified with C^{18} reverse phase column (H_2O:MeOH = 1:0 to 0:1) to afford compound **TD-EGGal$_2$** (92 mg, 95%) as a red amorphous solid.

^{1}H NMR (300 MHz, CD_3OD): δ (ppm) 1.37 (s, 9H, C(C$\underline{H}_3$)$_3$), 3.64-3.73 (m, 22H, OCH$_2$, H-2, H-3, H-4, H-5, H-6'), 3.82-3.99 (m, 10H, OC$\underline{H}_2$CH$_2$N, Gal-OCH$_2$, H-6), 4.23 (dd, 2H, J = 2.4 Hz, 7.2 Hz, H-1), 4.58 (m, 2H, OCH$_2$C$\underline{H}_2$N), 5.07 (s, 4H, OCH$_2$-triaz), 6.55 (d, 1H, J = 2.1 Hz, H-3pyran), 6.74-6.78 (m, 4H, H-5pyran, $\underline{H}$C=CH-Ph, 2×H-Ar), 6.90-7.10 (m, 13H, H-Ar), 7,35-7.38 (m, 3H, 2×H-Ar, HC=$\underline{C}$H-Ph), 8.09 (s, 2H, H-triaz).

^{13}C NMR (75 MHz, CD_3OD): 26.9 (C($\underline{C}$H$_3$)$_3$), 36.3 ($\underline{C}$(CH$_3$)$_3$), 50.1 (OCH$_2\underline{C}$H$_2$N), 57.7 ($\underline{C}$(CN)$_2$), 60.6 (O$\underline{C}$H$_2$-triaz), 68.2 (C-6), 68.87(Gal-OCH$_2$), 68.93 (O$\underline{C}$H$_2$CH$_2$N), 69.7 (OCH$_2$), 69.85 (OCH$_2$), 69.89 (OCH$_2$), 70.0 (C-4), 71.1 (C-2), 72.1 (C-5), 73.5 (C-3), 102.0 (C-3pyran), 103.6 (C-1), 106.6 (C-5pyran), 113.75 (CH-Ar), 113.94 (CH-Ar), 115.0 (2×CN), 118.0 (H$\underline{C}$=CH-Ph), 125.0 (CH^{triaz}), 127.3 (CH-Ar), 127.6 (CH-Ar), 131.1 (CH-Ar), 131.7 (CH-Ar), 131.8($\underline{C}^{Ar}$-CH=CH-Pyran), 132.37 (CH-Ar), 132.41 (CH-Ar), 132.7 (CH-Ar), 136.38 (C^{Ar}-C=C), 136.4 (C^{Ar}-C=C), 137.5 (HC=C$\underline{H}$-Ph), 138.9 ($\underline{C}^{Ar}$-C=C), 141.3 ($\underline{C}^{Ar}$-C=C), 143.7 (C=C), 144.1 (C^{triaz}), 146.6 (C=C), 156.0 (C-6pyran), 157.2 ($\underline{C}^{Ar}$-O), 157.3 ($\underline{C}^{Ar}$-O), 159.7 (C-2pyran), 172.8 (C-4pyran).

HR-ESI-MS *m*/*z*: calcd. for $C_{70}H_{84}N_8O_{19}$ [M+H]$^+$ 670.2921, found 670.2914.

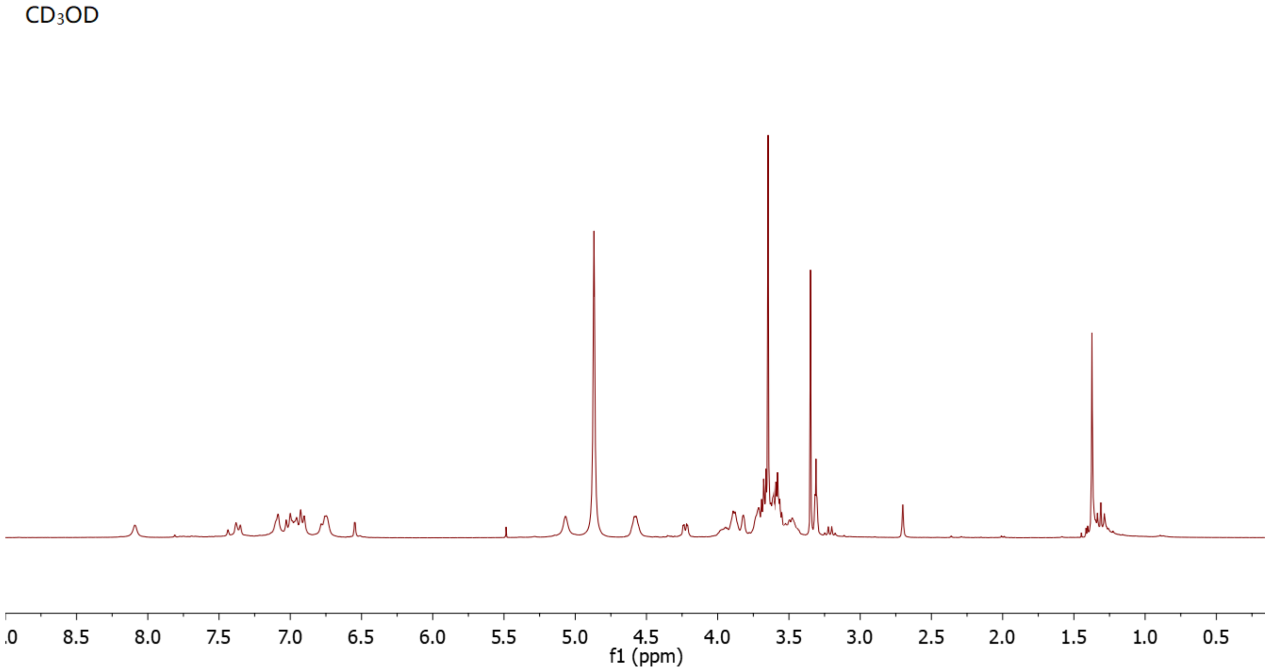
CD3OD
f1 (ppm)

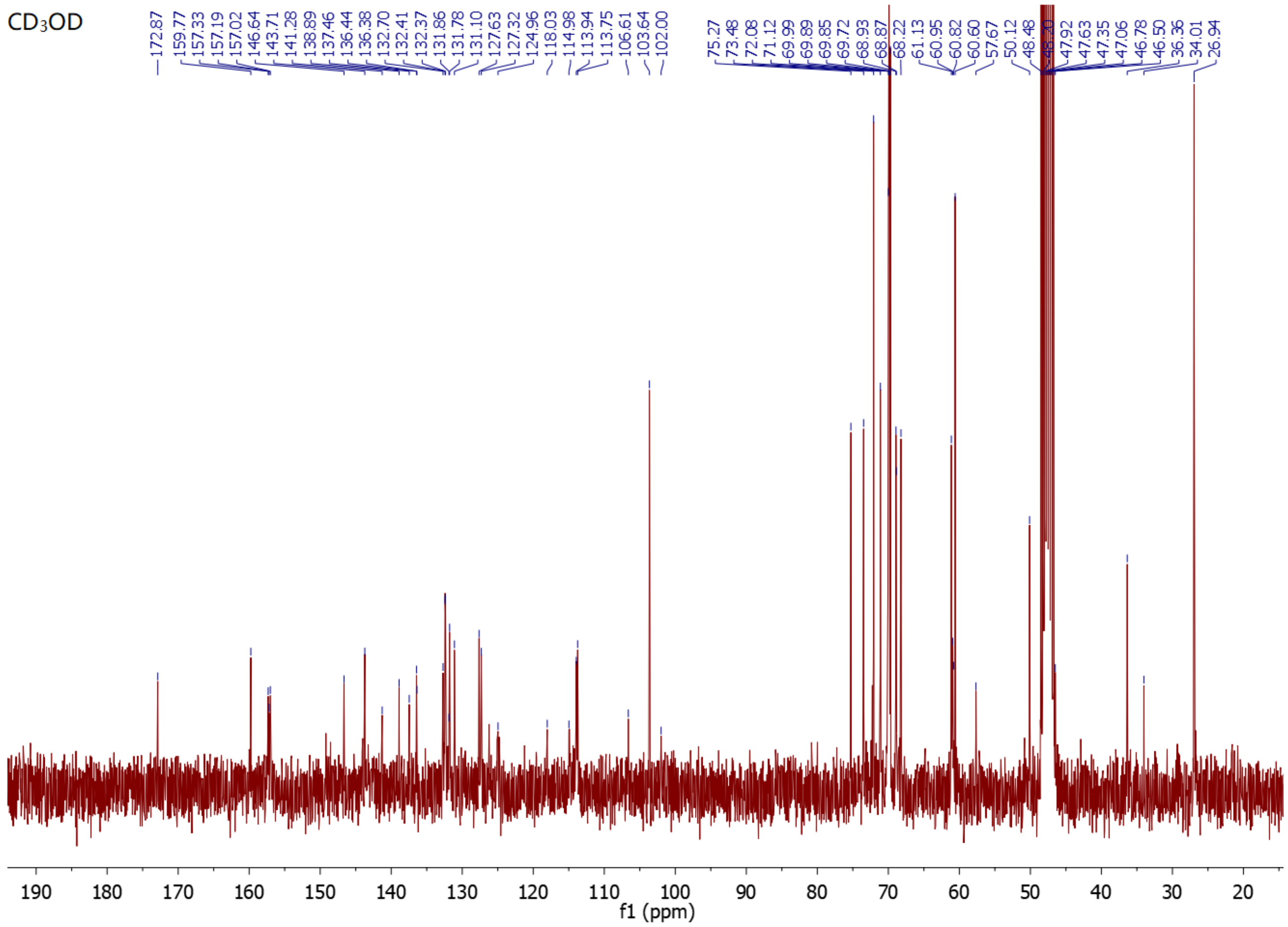
CD3OD
172.87
159.77
157.33
157.19
157.02
146.64
143.71
141.28
138.89
137.46
136.44
136.38
132.70
132.41
132.37
131.86
131.78
131.10
127.63
127.32
124.96
118.03
114.98
113.94
113.75
106.61
103.64
102.00
75.27
73.48
72.08
71.12
69.99
69.89
69.85
69.72
68.93
68.87
68.22
61.13
60.95
60.82
60.60
57.67
50.12
48.48
47.92
47.63
47.35
47.06
46.78
46.50
36.36
34.01
26.94
f1 (ppm)

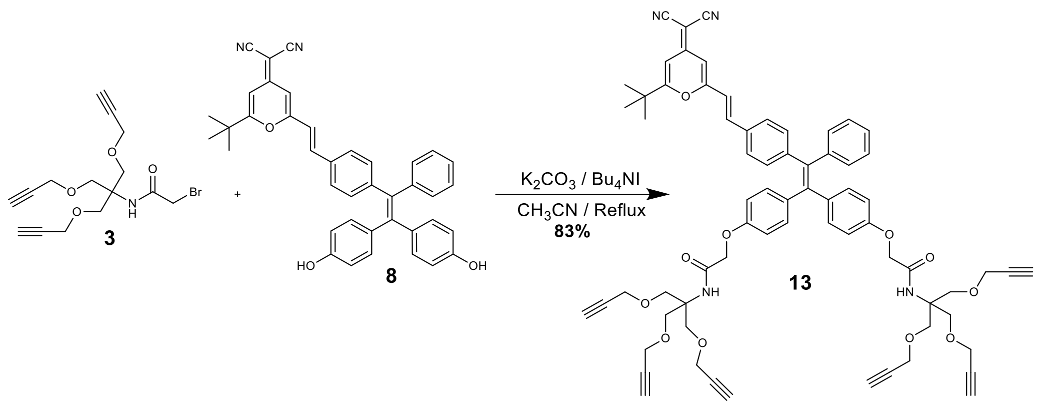

(*E*)-2-{2-[4-(2,2-di-{4-[*N*-tris(propargyloxymethyl)methamido-eth-2-yl]oxy}phenyl-1-phenyl-1-vinyl)styryl]-6-(*tert*-butyl)-4*H*-pyran-4-ylidene}-propanedinitrile (13):

To a solution of compound **3** (152 mg, 0.26 mmol, 1 eq.) and compound **8** (200 mg, 0.56 mmol, 2.2 eq.), potassium carbonate (141 mg, 1.02 mmol, 4 eq.) and tetrabutylammonium iodide (189 mg, 0.52 mmol, 2 eq.) in acetonitrile (15 mL) was added 18-crown-6 (35 mg, 0.13 mmol, 0.5 eq.). The resulting solution was stirred at 60°C during 18 h. The mixture was concentrated, diluted with EtOAc (50 mL), washed with brine (50 mL), HCl aqueous solution (30 mL, 2M) and brine (30 mL). All the aqueous fractions were combined and extracted with EtOAc (40 mL). The organic layers were combined, dried ($MgSO_4$), concentrated, and purified with silica gel column chromatography (Cyclohexane:EtOAc = 2.5:1) to obtain compound **13** (241 mg, 83%) as a red amorphous solid.

R_f = 0.17 (Cyclohexane:EtOAc = 2.5:1)

^{1}H NMR (300 MHz, $CDCl_3$): 1.37 (s, 9H, C(C$\underline{H}_3$)$_3$), 2.42-2.44 (m, 6H, HC≡C), 3.87 (s, 12H, (O-C$\underline{H}_2$)$_3$-C), 4.15 (d, 12H, *J* = 2.7 Hz, C$\underline{H}_2$-C≡CH), 4.34 (s, 4H, OC$\underline{H}_2$CO), 6.56 (d, 1H, *J* = 2.1 Hz, H-3pyran), 6.49-6.71 (m, 6H, H-5pyran, $\underline{H}$C=CH-Ph, 2×H-Ar), 6.88 (d, 2H, *J* = 3 Hz, NH), 6.39-6.70 (m, 8H, H-Ar), 7.14-7.16 (m, 3H, H-Ar), 7.29-7.32 (m, 3H, 2×H-Ar, HC=C$\underline{H}$-Ph).

^{13}C NMR (75 MHz, $CDCl_3$): 28.2 (C($\underline{C}$H$_3$)$_3$), 36.8 ($\underline{C}$(CH$_3$)$_3$) 58.7 ($\underline{C}$H$_2$-C≡CH), 59.3 ((O-CH$_2$)$_3$-$\underline{C}$), 59.7 ($\underline{C}$(CN)$_2$), 67.4 (O$\underline{C}$H$_2$CO), 68.4 ((O-$\underline{C}$H$_2$)$_3$-C), 74.85 and 74.9 (H$\underline{C}$≡C), 79.51 and 79.54 (HC≡$\underline{C}$), 102.7 (C-3pyran), 107.2 (C-5pyran), 114.2 (CH-Ar), 114.3 (CH-Ar), 115.2 and 115.23 (2×CN), 118.1 (H$\underline{C}$=CH-Ph), 126.8 (CH-Ar), 127.4 (CH-Ar), 128.1 (CH-Ar) , 131.4 (CH-Ar), 132.1 (CH-Ar), 132.4 ($\underline{C}^{Ar}$-CH=CH-Pyran), 132.7 (CH-Ar), 132.8 (CH-Ar), 137.1 (C^{Ar}-C=C), 137.2(C^{Ar}-C=C), 137.5 (HC=C$\underline{H}$-Ph), 139.3 ($\underline{C}^{Ar}$-C=C), 140.8 ($\underline{C}^{Ar}$-C=C), 143.4 (C=C), 146.7 (C=C), 156.0 (C-6pyran), 156.1 ($\underline{C}^{Ar}$-O-propargyl), 156.6 ($\underline{C}^{Ar}$-O-propargyl), 158.9(C-2pyran), 167.8 and 167.87 (CH$_2\underline{C}$ONH)172.2 (C-4pyran).

HR-ESI-MS *m*/*z*: calcd. for $C_{70}H_{67}N_4O_{11}$ $[M+H]^+$ 1139.4801, found 1139.4807.

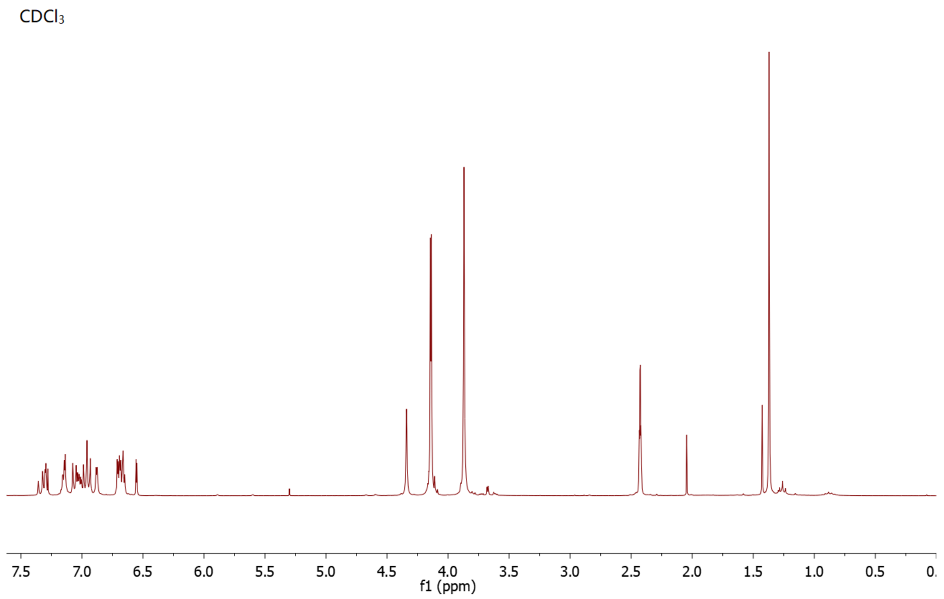
CDCl3
7.5 7.0 6.5 6.0 5.5 5.0 4.5 4.0 3.5 3.0 2.5 2.0 1.5 1.0 0.5 0.
f1 (ppm)

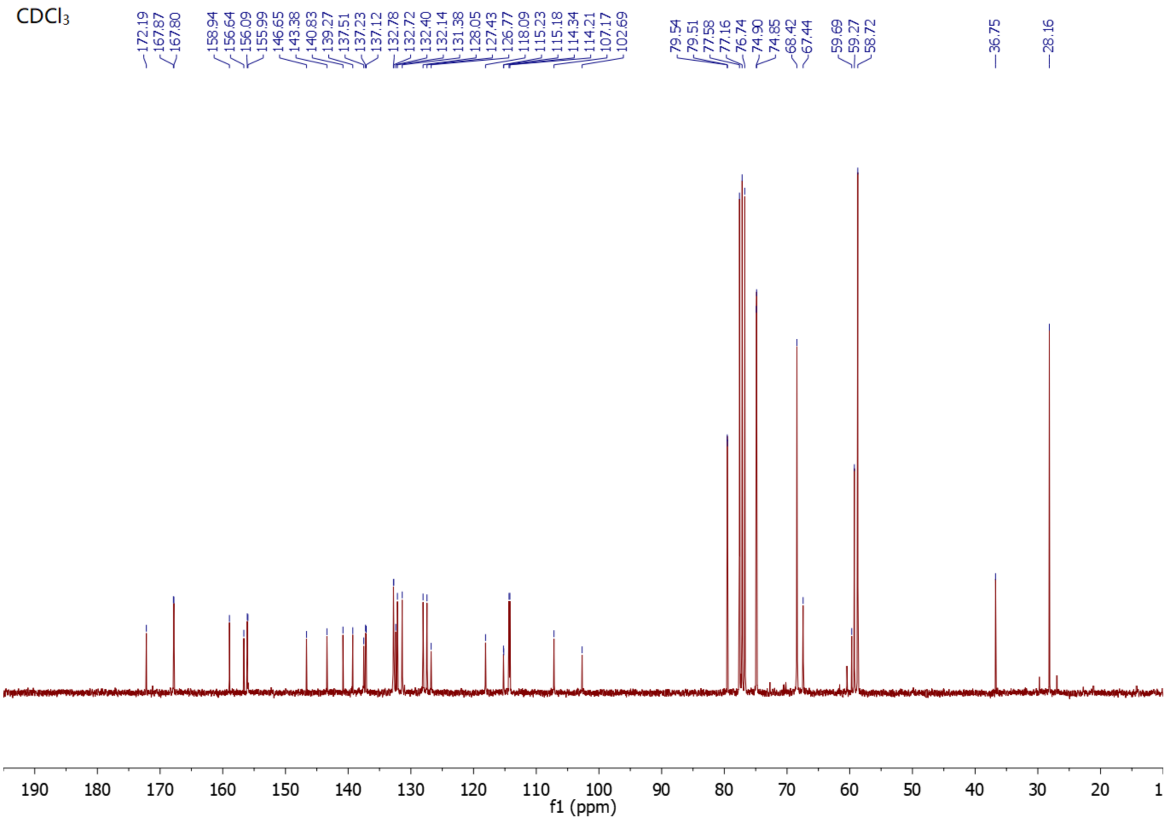
CDCl3
172.19 167.87 167.80 158.94 156.64 156.09 155.99 146.65 143.38 140.83 139.27 137.51 137.23 137.12 132.78 132.72 132.40 132.14 131.38 128.05 127.43 126.77 118.09 115.23 115.18 114.34 114.21 107.17 102.69
79.54 79.51 77.58 77.16 76.74 74.90 74.85 68.42 67.44 59.69 59.27 58.72 36.75 28.16
190 180 170 160 150 140 130 120 110 100 90 80 70 60 50 40 30 20 1
f1 (ppm)

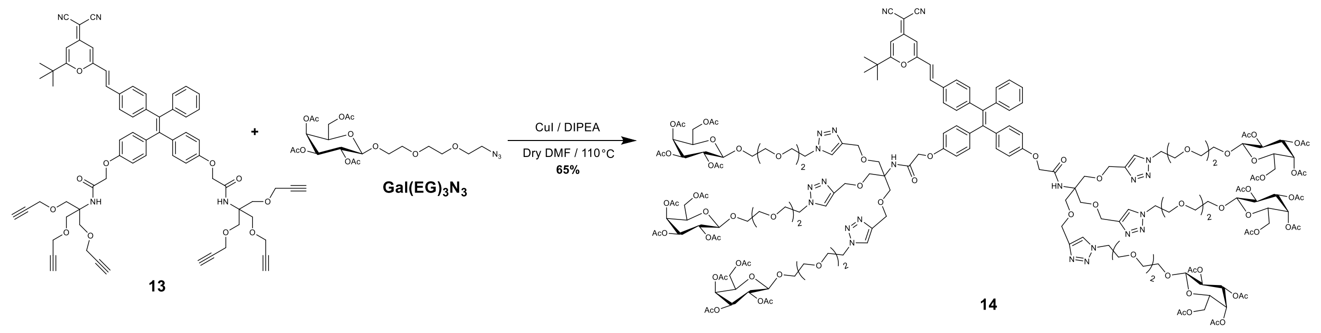

(*E*)-2-{2-[4-(2,2-di-{4-[*N*-tris({1-[1-(2,3,4,6-tetra-*O*-acetyl-β-D-galacopyranosyloxy)-3,6-dioxaoct-8-yl]-1,2,3-triazol-4-yl-methoxy}methyl)methamido-eth-2-yl]oxy}phenyl-1-phenyl-1-vinyl)styryl]-6-(*tert*-butyl)-4*H*-pyran-4-ylidene}-propanedinitrile (14):

To a solution of compound **13** (100 mg, 0.088 mmol, 1 eq.), compound Gal(EG)$_3$N$_3$ (354 mg, 0.7 mmol, 8 eq.) and copper iodide (10 mg, 0.052 mmol, 0.6 eq.) in dry DMF (5 mL) was added *N*,*N*-diisopropylethylamine (0.13 mL, 0.7 mmol, 8 eq.). The resulting mixture was stirred under microwaves activation at 110°C during 60 min. Then the mixture was diluted with EtOAc (100 mL), washed with saturated aqueous EDTA solution (3×50 mL). The organic layer was dried (MgSO$_4$), concentrated and purified with silica gel column chromatography (CH$_2$Cl$_2$:MeOH = 25:1) to obtain compound **14** (238 mg, 65%) as red amorphous solid.

R$_f$ = 0.26 (CH$_2$Cl$_2$:MeOH = 20:1)

^{1}H NMR (300 MHz, CDCl$_3$): 1.35 (s, 9H, C(C$\underline{H}_3$)$_3$), 1.96, 2.02, 2.021, 2.12 (4s, 4×18H, CH$_3$CO), 3.57-3.61 (m, 36H, OCH$_2$CH$_2$O), 3.67-3.74 (m, 8H, Gal-OC$\underline{H}_2$), 3.80 (s, 12H, (O-C$\underline{H}_2$)$_3$-C), 3.85-3.97 (m, 24H, OC$\underline{H}_2$CH$_2$N, H-5, Gal-OC$\underline{H}_2$), 4.08-4.19 (m, 12H, H-6, H-6'), 4.28 (s, 4H, OC$\underline{H}_2$CO), 4.50-4.61 (m, 30H, H-1, OCH$_2$C$\underline{H}_2$N, OCH$_2$-triaz), 5.02 (dd, 6H, *J* = 3.3 Hz, 10.5 Hz, H-3), 5.14-5.21 (m, 6H, H-2), 5.37 (dd, 6H, *J* = 3.3 Hz, 10.5 Hz, H-4), 6.53 (d, 2H, *J* = 2.1 Hz, H-3pyran), 6.60-6.75 (m, 6H, H-5pyran, $\underline{H}$C=CH-Ph, 2×H-Ar), 6.90-7.04 (m, 10H, 2×NH, 8×H-Ar), 7.14-7.16 (m, 3H, H-Ar), 7.29-7.32 (m, 3H, 2×H-Ar, HC=C$\underline{H}$-Ph), 7.70 (s, 6H, H-triaz).

^{13}C NMR (75 MHz, CDCl$_3$): 20.5, 20.56, 20.58, 20.7 (4×CO$\underline{C}$H$_3$), 28.0 (C($\underline{C}$H$_3$)$_3$), 36.6 ($\underline{C}$(CH$_3$)$_3$), 50.0 (OCH$_2\underline{C}$H$_2$N), 53.5 ((O-CH$_2$)$_3$-$\underline{C}$), 59.6($\underline{C}$(CN)$_2$), 61.1 (C-6), 64.6 (O$\underline{C}$H$_2$-triaz), 67.0 (C-4), 67.1 (O$\underline{C}$H$_2$CO), 68.6 (C-2), 68.7 ((O-$\underline{C}$H$_2$)$_3$-C), 69.0 (Gal-O$\underline{C}$H$_2$), 69.3 (O$\underline{C}$H$_2$CH$_2$N), 70.1 (C-5), 70.37 (OCH$_2$), 70.47 (OCH$_2$), 70.5 (OCH$_2$), 70.7 (C-3), 101.2 (C-1), 102.5 (C-3pyran), 107.0 (C-5pyran), 113.9 (CH-Ar), 114.1 (CH-Ar), 115.1 (2×CN), 118.0 (H$\underline{C}$=CH-Ph), 123.7 (CH^{triaz}), 126.6 (CH-Ar), 127.3 (CH-Ar), 127.9 (CH-Ar), 131.2 (CH-Ar), 131.94 (CH-Ar), 131.98 (CH-Ar), 132.3($\underline{C}^{Ar}$-CH=CH-Pyran), 132.6 (CH-Ar), 136.90 (C^{Ar}-C=C), 136.92 (C^{Ar}-C=C), 137.4 (HC=C$\underline{H}$-Ph), 139.1 ($\underline{C}^{Ar}$-C=C), 140.7 ($\underline{C}^{Ar}$-C=C), 143.3 (C=C), 144.3 and 144.4 (C^{triaz}), 146.4 (C=C), 155.7 (C-6pyran), 155.9 ($\underline{C}^{Ar}$-O), 156.6 ($\underline{C}^{Ar}$-O), 158.9 (C-2pyran), 167.4 (NH$\underline{C}$OCH$_2$) 169.3, 170.0, 170.1, 170.2 (4×$\underline{C}$OCH$_3$), 172.1 (C-4pyran).

HR-ESI-MS *m*/*z*: calcd. for C$_{190}$H$_{252}$N$_{22}$Na$_4$O$_{83}$ [M+4Na]$^{4+}$ 1065.3936, found 1065.3937.

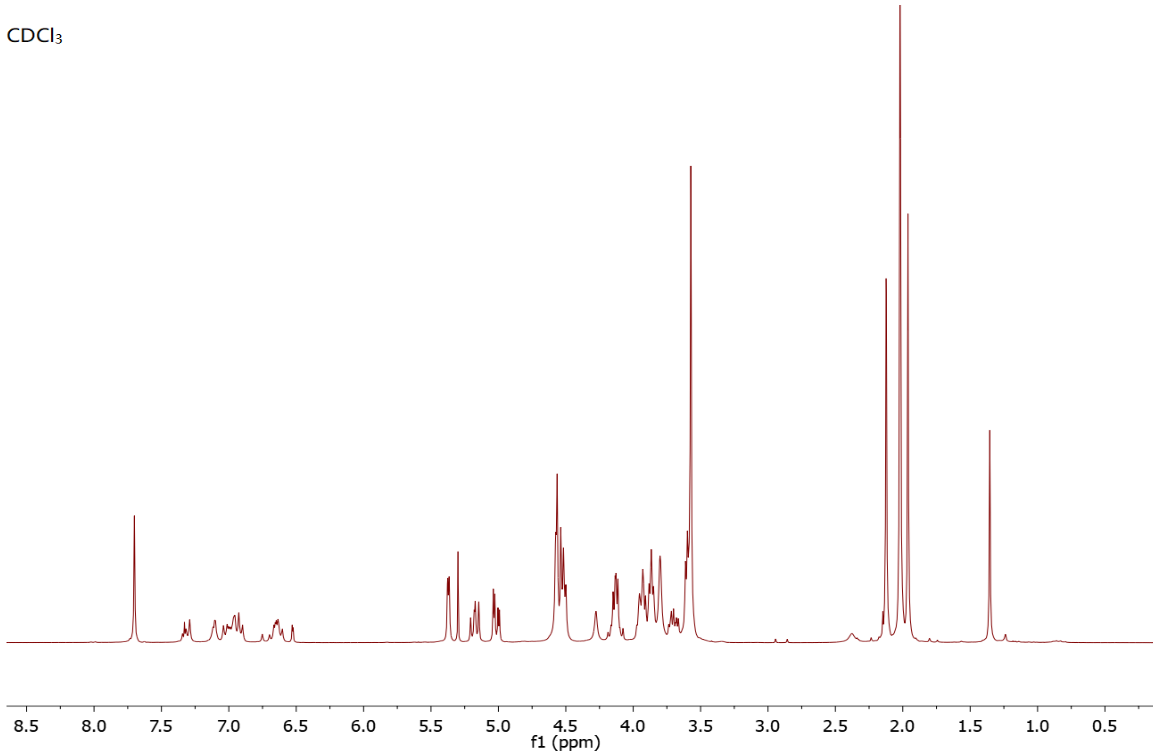
CDCl3
8.5 8.0 7.5 7.0 6.5 6.0 5.5 5.0 4.5 4.0 3.5 3.0 2.5 2.0 1.5 1.0 0.5
f1 (ppm)

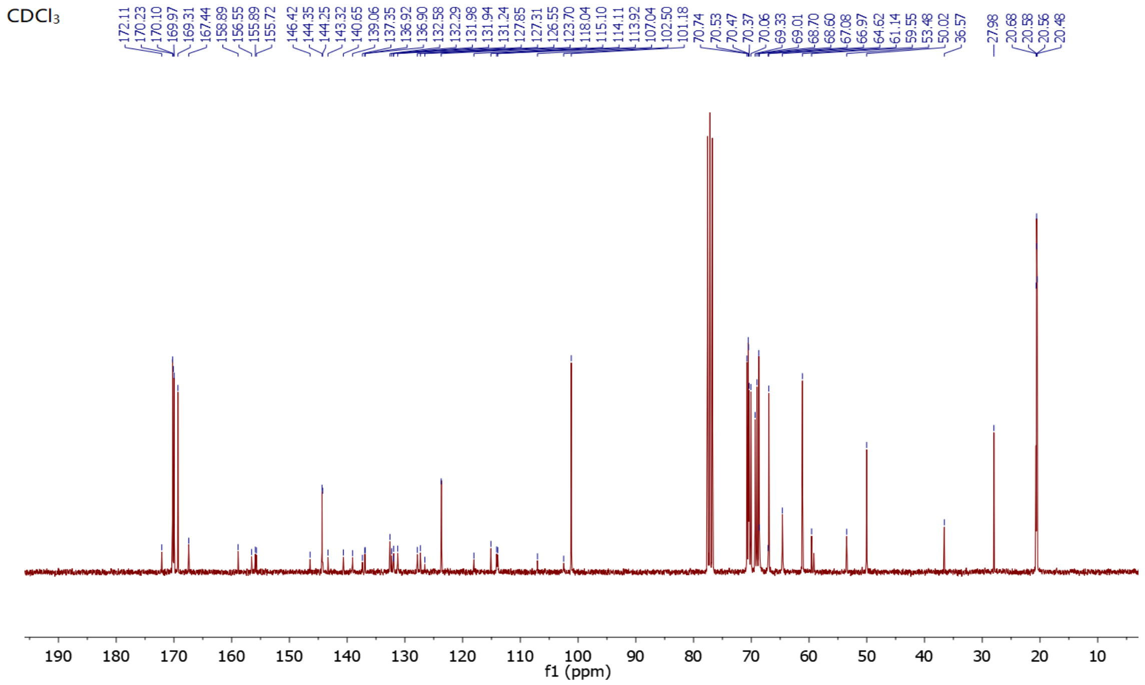
CDCl3
172.11 170.23 170.10 169.97 169.31 167.44 158.89 156.55 155.89 155.72 146.42 144.35 144.25 143.32 140.65 139.06 137.35 136.92 136.90 132.58 132.29 131.98 131.94 131.24 127.85 127.31 126.55 123.70 118.04 115.10 114.11 113.92 107.04 102.50 101.18 70.74 70.53 70.47 70.37 70.06 69.33 69.01 68.70 68.60 67.08 66.97 64.62 61.14 59.55 53.48 50.02 36.57 27.98 20.68 20.58 20.56 20.48
190 180 170 160 150 140 130 120 110 100 90 80 70 60 50 40 30 20 10
f1 (ppm)

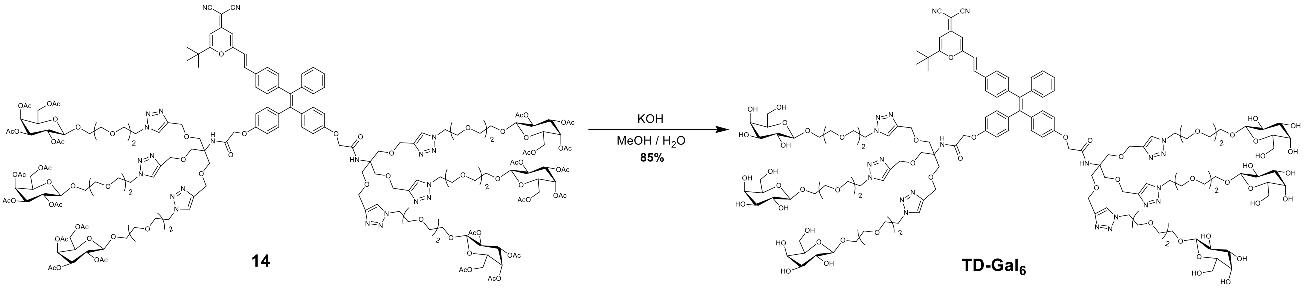

(*E*)-2-{2-[4-(2,2-di-{4-[*N*-tris({1-[1-(β-D-galacopyranosyloxy)-3,6-dioxaoct-8-yl]-1,2,3-triazol-4-yl-meth-oxy}methyl)methamido-eth-2-yl]oxy}phenyl-1-phenyl-1-vinyl)styryl]-6-(*tert*-butyl)-4*H*-pyran-4-yli-dene}-propanedinitrile (**TD-Gal$_6$**):

To a solution of compound **14** (238 mg, 0.057 mmol, 1 eq.) in MeOH/H_2O (12 mL, 5:1, v/v) was added KOH powder (84 mg, 1.48 mmol, 26 eq.). The resulting mixture was stirred under argon at RT during 90 min. The solution was neutralized (pH = 7) with 0.6 M HCl aqueous solution and concentrated. The crude product was dissolved with H_2O (4 mL) and purified with C^{18} reverse phase column (H_2O:MeOH = 1:0 to 0:1) to afford compound **TD-Gal$_6$** (153 mg, 85%) as a red amorphous solid.

^{1}H NMR (300 MHz, D_2O/CD_3OD): 1.48 (s, 9H, C(CH_3)$_3$), 3.66-3.77 (m, 54H, OCH_2, H-2, H-3, H-5), 3.82-3.92 (m, 30H, Gal-OCH_2, (O-CH_2)$_3$-C, H-6'), 4.00-4.14 (OCH_2CH_2N, H-4, H-6), 4.43 (dd, 6H, *J* = 2.7 Hz, 5.4 Hz, H-1), 4.51 (s, 4H, OCH_2CO), 4.67-4.72 (m, 24H, OCH_2CH_2N, OCH_2-triaz), 6.67 (d, 1H, *J* = 1.5 Hz, H-3pyran), 6.78-6.86 (m, 6H, H-5pyran, HC=CH-Ph, 2×H-Ar), 7.00-7.13 (m, 10H, 2×NH, 8×H-Ar), 7.23-7.24 (m, 3H, H-Ar), 7.47-7.53 (m, 3H, 2×H-Ar, HC=CH-Ph),

8.13, 8.16 (bs, 6H, H-triaz).

^{13}C NMR (75 MHz, D_2O/CD_3OD): 28.3 (C(CH_3)$_3$), 37.6 (C(CH_3)$_3$), 51.3 (OCH_2CH_2N), 58.0 (C(CN)$_2$), 60.9 ((O-CH_2)$_3$-C), 62.2 (Gal-OCH_2), 65.1 (OCH_2-triaz), 68.2 (OCH_2CO), 69.55 (OCH_2CH_2N), 69.56 (C-6), 70.0 ((O-CH_2)$_3$-C), 70.07 (C-4), 71.0 (OCH_2), 71.1 (OCH_2), 71.2 (OCH_2), 72.3 (C-2), 74.5 (C-5), 76.4 (C-3), 103.4 (C-3pyran), 104.6 (C-1), 108.0 (C-5pyran), 115.2 (CH-Ar), 115.4 (CH-Ar), 116. 5 (2×CN), 119.4 (HC=CH-Ph), 126.2 (CH^{triaz}), 127.8 (CH-Ar), 128.6 (CH-Ar), 129.0 (CH-Ar), 131.2 (CH-Ar), 132.9 (CH-Ar), 133.52 (CH-Ar), 133.57 (CH-Ar), 133.9 (C^{Ar}-CH=CH-Pyran), 138.28 (C^{Ar}-C=C), 138.3 (C^{Ar}-C=C), 138.3 (HC=CH-Ph), 140.7 (C^{Ar}-C=C), 142.0 (C^{Ar}-C=C), 144.5 (C^{triaz}), 147.6 (C=C), 157.4 (C^{Ar}-O), 157.5(C^{Ar}-O), 158.8 (C-6pyran), 161.4 (C-2pyran), 170.5 ($NHCOCH_2$), 174.7 (C-4pyran).

HR-ESI-MS *m*/*z*: calcd. for $C_{142}H_{207}N_{22}O_{59}$ $[M+3H]^{3+}$ 1054.7952, found 1054.7901.

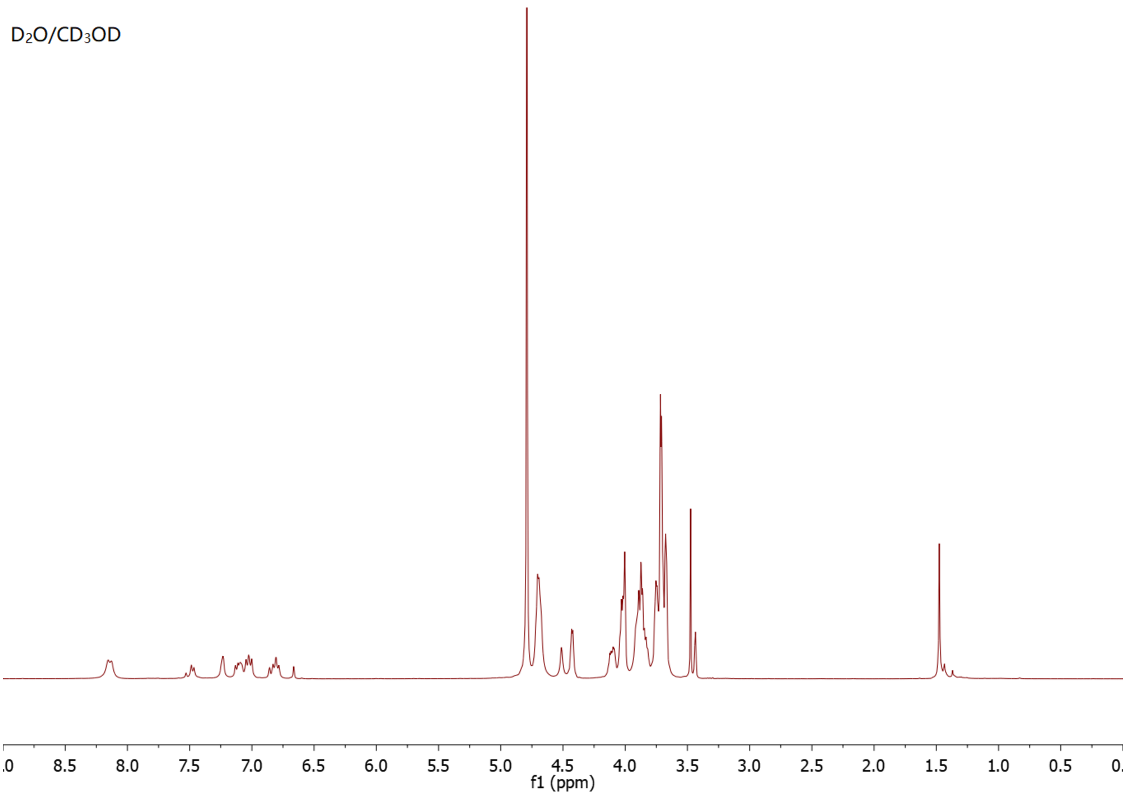
D2O/CD3OD
9.0 8.5 8.0 7.5 7.0 6.5 6.0 5.5 5.0 4.5 4.0 3.5 3.0 2.5 2.0 1.5 1.0 0.5 0.
f1 (ppm)

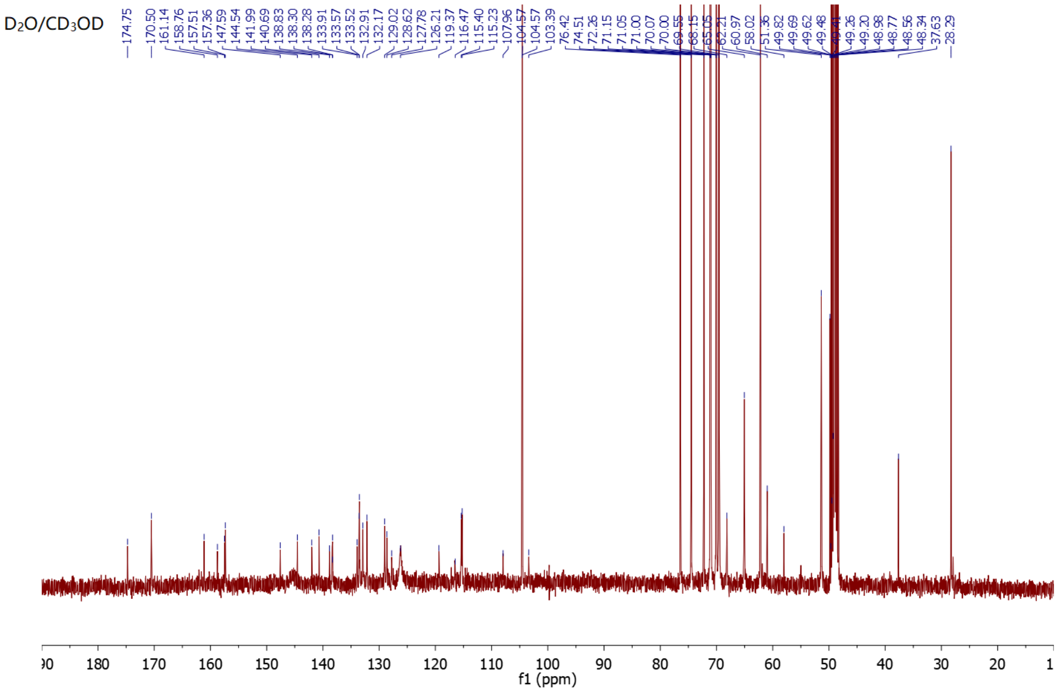
D2O/CD3OD
174.75
170.50
161.14
158.76
157.51
157.36
147.59
144.54
141.99
140.69
138.83
138.30
138.28
133.91
133.57
133.52
132.91
132.17
129.02
128.62
127.78
126.21
119.37
116.47
115.40
115.23
107.96
104.57
104.57
103.39
76.42
74.51
72.26
71.15
71.05
71.00
70.07
70.00
69.55
68.15
65.05
62.21
60.97
58.02
51.36
49.82
49.69
49.62
49.48
49.26
49.20
48.98
48.77
48.56
48.34
37.63
28.29
190 180 170 160 150 140 130 120 110 100 90 80 70 60 50 40 30 20 10
f1 (ppm)

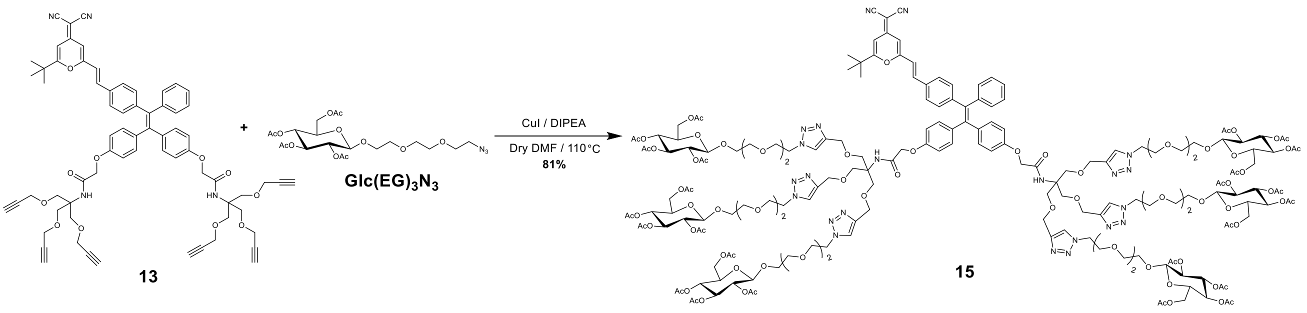

(*E*)-2-{2-[4-(2,2-di-4-[*N*-tris({1-[1-(2,3,4,6-tetra-*O*-acetyl-β-D-glucopyranosyloxy)-3,6-dioxaoct-8-yl]-1,2,3-triazol-4-yl-methoxy}methyl)methamido-eth-2-yl]oxy}phenyl-1-phenyl-1-vinyl)styryl]-6-(*tert*-butyl)-4*H*-pyran-4-ylidene}-propanedinitrile (15):

To a solution of compound **13** (90 mg, 0.079 mmol, 1 eq.), compound Glc(EG)$_3$N$_3$ (320 mg, 0.63 mmol, 8 eq.) and copper iodide (9 mg, 0.047 mmol, 0.6 eq.) in dry DMF (4 mL) was added *N*,*N*-diisopropylethylamine (0.1 mL, 0.63 mmol, 8 eq.). The resulting mixture was stirred under microwaves activation at 110°C during 90 min. Then the mixture was diluted with EtOAc (50 mL), washed with saturated aqueous EDTA solution (3x50 mL). The combined aqueous fractions was extracted with EtOAc (30 mL). The organic layers were combined, dried ($MgSO_4$), concentrated and purified with silica gel column chromatography (CH_2Cl_2:MeOH = 25:1) to obtain compound **15** (266 mg, 81%) as red amorphous solid.

R_f = 0.20 (CH_2Cl_2:MeOH = 25:1)

^{1}H NMR (300 MHz, $CDCl_3$): 1.30 (s, 9H, $C(C\underline{H}_3)_3$), 1.92, 1.94, 1.941, 2.00 (4s, 4×18H, CH_3CO), 3.51-3.57 (m, 36H, OCH_2CH_2O), 3.63-3.71 (m, 12H, H-5, Glc-$OC\underline{H}_2$), 3.74 (s, 12H, $(O\text{-}C\underline{H}_2)_3$-C), 3.79-3.90 (m, 18H, $OC\underline{H}_2CH_2N$, Glc-$OC\underline{H}_2$), 4.06 (dd, 6H, *J* = 2.4 Hz, 12.3 Hz, H-6'), 4.16-4.22 (m, 10H, H-6, $OC\underline{H}_2CO$), 4.54 (t, 12H, *J* = 5.4 Hz, $OCH_2C\underline{H}_2N$), 4.50-4.54 (m, 18H, H-1, $OC\underline{H}_2$-triaz), 4.88-4.94 (m, 6H, H-2), 4.97-5.04 (m, 6H, H-4), 5.14 (t, 6H, *J* = 9.3 Hz, H-3), 6.47 (d, 2H, *J* = 1.8 Hz, H-3pyran), 6.54-6.69 (m, 6H, H-5pyran, $\underline{H}$C=CH-Ph, 2×H-Ar), 6.84-6.98 (m, 10H, 2×NH, 8×H-Ar), 7.04-7.06 (m, 3H, H-Ar), 7.23-7.28 (m, 3H, 2×H-Ar, HC=C$\underline{H}$-Ph), 7.63 (s, 6H, H-triaz).

^{13}C NMR (75 MHz, $CDCl_3$): 20.55, 20.56, 20.6, 20.7 (4×$CO\underline{C}H_3$), 28.0 ($C(\underline{C}H_3)_3$), 36.6 ($\underline{C}(CH_3)_3$), 50.1 ($OCH_2\underline{C}H_2N$), 53.5 ($(O\text{-}CH_2)_3$-$\underline{C}$), 59.6($\underline{C}(CN)_2$), 61.9 (C-6), 64.7 ($O\underline{C}H_2$-triaz), 67.1 ($O\underline{C}H_2CO$), 68.3 (C-4), 68.6 ($(O\text{-}\underline{C}H_2)_3$-C), 69.1(Glc-$OCH_2$), 69.4 ($O\underline{C}H_2CH_2N$), 70.1 ($OCH_2$), 70.4 ($OCH_2$), 70.5 ($OCH_2$), 71.2 (C-2), 71.7 (C-5), 72.7 (C-3), 100.7 (C-1), 102.5 (C-3pyran), 107.1 (C-5pyran), 114.0 (CH-Ar), 114.2 (CH-Ar), 115.1 (2×CN), 118.0 (H$\underline{C}$=CH-Ph), 123.68 and 123.73 (CH^{triaz}), 126.6 (CH-Ar), 127.4 (CH-Ar), 127.9 (CH-Ar), 131.25 (CH-Ar), 131.29 (CH-Ar), 132.0 (CH-Ar), 132.3($\underline{C}^{Ar}$-CH=CH-Pyran), 132.6 (CH-Ar), 136.94 (C^{Ar}-C=C), 136.96 (C^{Ar}-C=C), 137.4 (HC=C$\underline{H}$-Ph), 139.1 ($\underline{C}^{Ar}$-C=C), 140.7 ($\underline{C}^{Ar}$-C=C), 143.4 (C=C), 144.3 and 144.4 (C^{triaz}), 146.5 (C=C), 155.8 (C-6pyran), 155.9 ($\underline{C}^{Ar}$-O), 156.6 ($\underline{C}^{Ar}$-O), 158.9 (C-2pyran), 167.4 ($NH\underline{C}OCH_2$) 169.2, 169.3, 170.1, 170.5 (4×$\underline{C}OCH_3$), 172.2 (C-4pyran).

HR-ESI-MS *m*/*z*: calcd. for $C_{190}H_{256}N_{22}O_{83}$ $[M+4H]^{4+}$ 1043.4116, found 1043.4168.

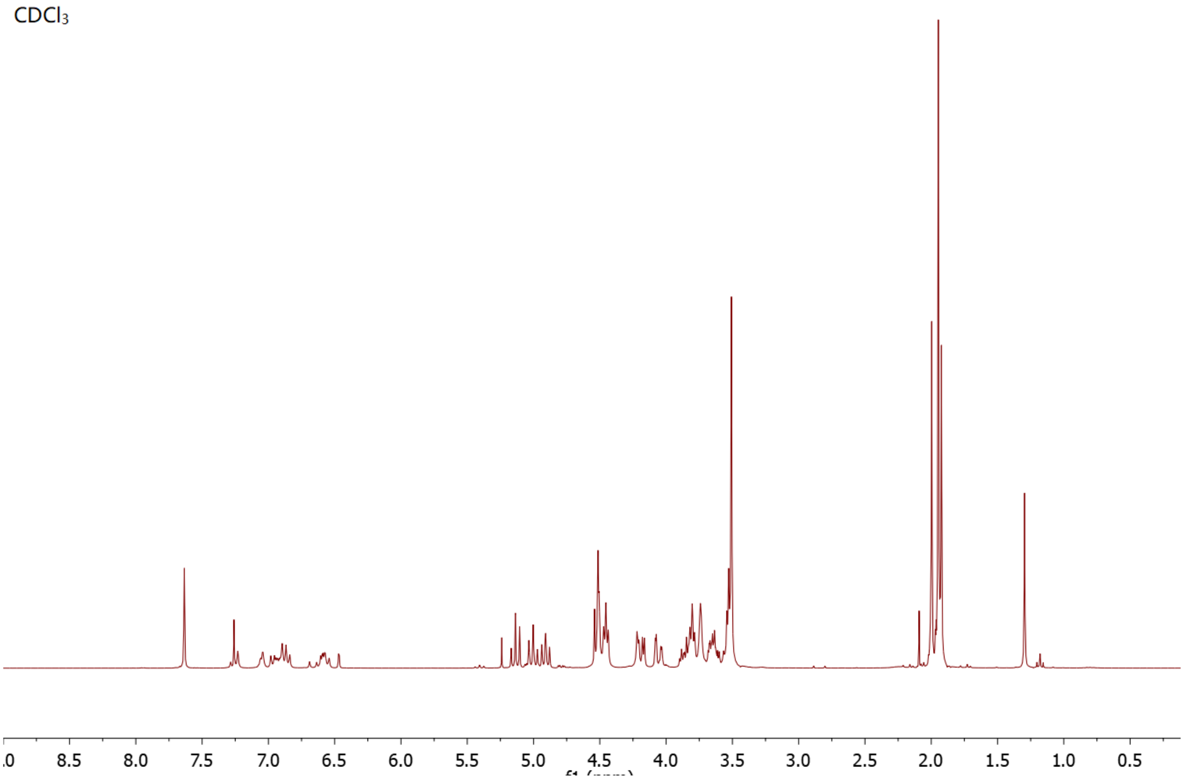
CDCl3
f1 (ppm)

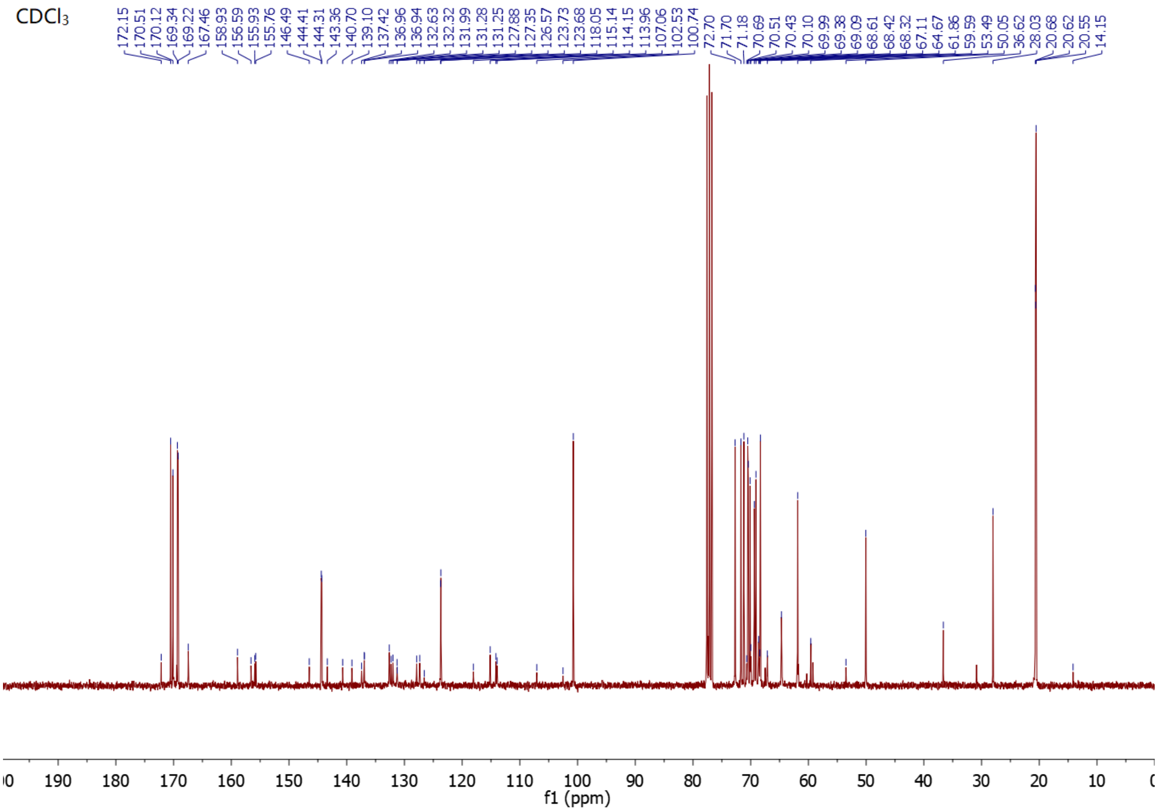
CDCl3
172.15
170.51
170.12
169.34
169.22
167.46
158.93
156.59
155.93
155.76
146.49
144.41
144.31
143.36
140.70
139.10
137.42
136.96
136.94
132.63
132.32
131.99
131.28
131.25
127.88
127.35
126.57
123.73
123.68
118.05
115.14
114.15
113.96
107.06
102.53
100.74
72.70
71.70
71.18
70.69
70.51
70.43
70.10
69.99
69.38
69.09
68.61
68.42
68.32
67.11
64.67
61.86
59.59
53.49
50.05
36.62
28.03
20.68
20.62
20.55
14.15
f1 (ppm)

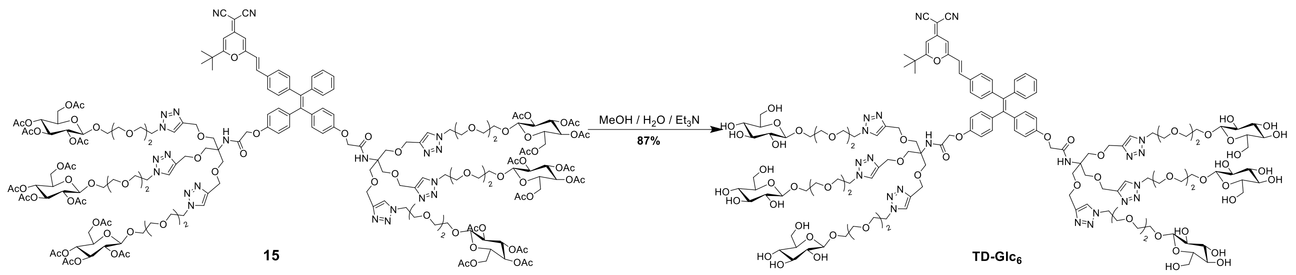

(*E*)-2-{2-[4-(2,2-di-{4-[*N*-tris({1-[1-(β-D-glucopyranosyloxy)-3,6-dioxaoct-8-yl]-1,2,3-triazol-4-yl-meth-oxy}methyl)methamido-eth-2-yl]oxy}phenyl-1-phenyl-1-vinyl)styryl]-6-(*tert*-butyl)-4*H*-pyran-4-ylidene}-propanedinitrile (TD-Glc$_6$):

To a solution of compound **15** (260 mg, 0.062 mmol, 1 eq.) in mixed solvent with MeOH (14 mL) and H_2O (3 mL) was added Et_3N (3 mL). The resulting mixture was stirred under argon at 35°C during 20 h. The solution was evaporated Et_3N after starting materials disappeared by TLC tracing. The crude product was dissolved with H_2O (4 mL) and purified with C^{18} reverse phase column (H_2O:MeOH = 1:0 to 0:1) to afford compound **TD-Glc$_6$** (170 mg, 87%) as a red amorphous solid.

^{1}H NMR (300 MHz, D_2O/CD_3OD): 1.60 (s, 9H, C(C$\underline{H}_3$)$_3$), 3.43-3.49 (m, 24H, H-2, H-3, H-4, H-5), 3.78-3.87 (m, 36H, OCH_2), 3.89-3.98 (m, 12H, Glc-OCH_2, H-6'), 4.00-4.06 (m, 12H, (O-C$\underline{H}_2$)$_3$-C), 4.08-4.15 (m, 18H, H-6, OC$\underline{H}_2$CH$_2$N), 4.17-4.23 (m, 6H, Glc-OCH_2), 4.57 (dd, 6H, J = 2.1 Hz, 6.0 Hz, H-1), 4.61 (s, 4H, OC$\underline{H}_2$CO), 4.78-4.79 (m, 24H, OCH$_2$C$\underline{H}_2$N, OCH$_2$-triaz), 6.79 (d, 1H, J = 1.5 Hz, H-3pyran), 6.90-6.99 (m, 6H, H-5pyran, $\underline{H}$C=CH-Ph, 2×H-Ar), 7.12-7.26 (m, 10H, 2×NH, 8×H-Ar), 7.33-7.37 (m, 3H, H-Ar), 7.60-7.66 (m, 3H, 2×H-Ar, HC=C$\underline{H}$-Ph), 8.21, 8.23 (bs, 6H, H-triaz).

^{13}C NMR (75 MHz, D_2O/CD_3OD): 28.5 (C($\underline{C}$H$_3$)$_3$), 37.9 ($\underline{C}$(CH$_3$)$_3$), 51.6 (OCH$_2\underline{C}$H$_2$N), 58.4 ($\underline{C}$(CN)$_2$), 61.3 ((O-CH$_2$)$_3$-$\underline{C}$), 62.8 (Gal-OCH$_2$), 65.4 (O$\underline{C}$H$_2$-triaz), 68.2 (O$\underline{C}$H$_2$CO), 69.8 (C-6), 69.9 ((O-$\underline{C}$H$_2$)$_3$-C), 70.3 (O$\underline{C}$H$_2$CH$_2$N), 71.3 (OCH$_2$), 71.36 (OCH$_2$), 71.4 (OCH$_2$), 71.6 (C-2), 75.0 (C-5), 77.85 (C-3), 77.89 (C-4), 103.6 (C-3pyran), 104.3 (C-1), 108.1 (C-5pyran), 115.5 (CH-Ar), 115.7 (CH-Ar), 116.7 (2×CN), 119.7 (H$\underline{C}$=CH-Ph), 126.2 (CH^{triaz}), 127.9 (CH-Ar), 128.8 (CH-Ar), 129.2 (CH-Ar), 132.4 (CH-Ar), 133.1 (CH-Ar), 133.74 (CH-Ar), 133.79 (CH-Ar), 134.2 ($\underline{C}^{Ar}$-CH=CH-Pyran), 138.56 (C^{Ar}-C=C), 138.62 (C^{Ar}-C=C), 139.1 (HC=C$\underline{H}$-Ph), 141.0 ($\underline{C}^{Ar}$-C=C), 142.2 ($\underline{C}^{Ar}$-C=C), 144.8 (C^{triaz}), 147.8 (C=C), 157.7 ($\underline{C}^{Ar}$-O), 157.8 ($\underline{C}^{Ar}$-O), 158.9 (C-6pyran), 161.4 (C-2pyran), 170.6 (NH$\underline{C}$OCH$_2$), 174.9 (C-4pyran).

HR-ESI-MS m/z: calcd. for $C_{142}H_{207}N_{22}O_{59}$ $[M+3H]^{3+}$ 1054.7952, found 1054.7942.

D$_2$O/CD$_3$OD

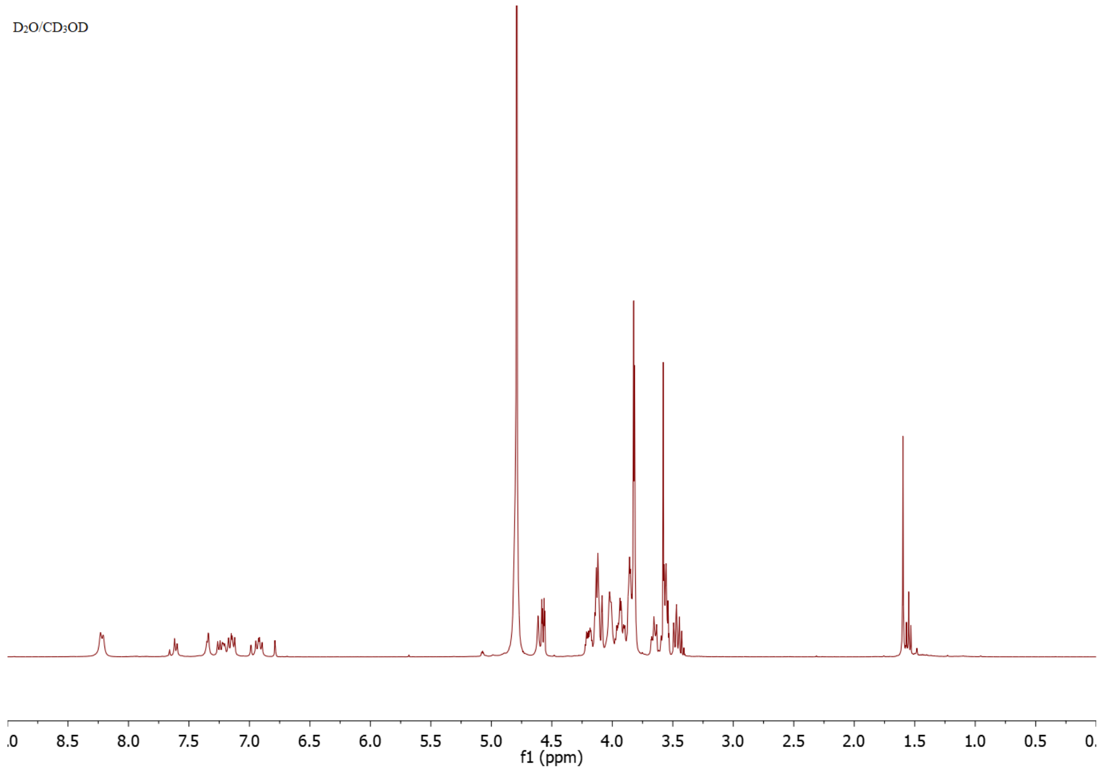

D$_2$O/CD$_3$OD

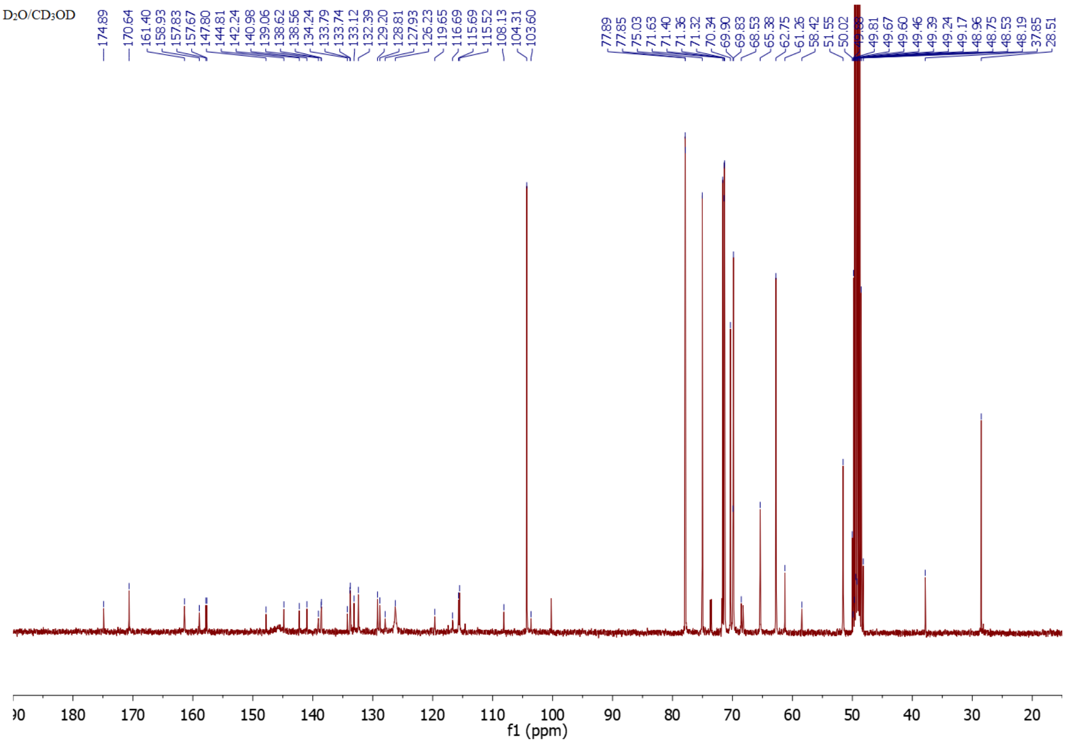

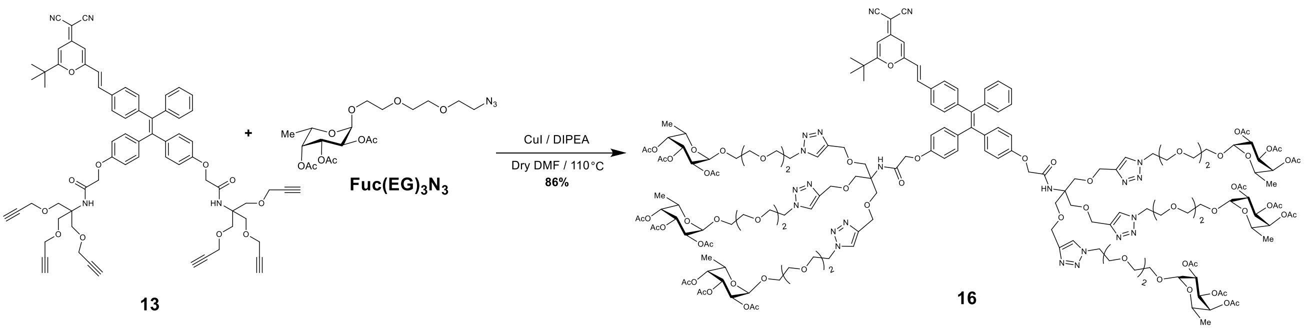

13 16

(*E*)-2-{2-[4-(2,2-di-{4-[*N*-tris({1-[1-(2,3,4-tri-*O*-acetyl-*α*-L-fucopyranosyloxy)-3,6-dioxaoct-8-yl]-1,2,3-triazol-4-yl-methoxy}methyl)methamido-eth-2-yl]oxy}phenyl-1-phenyl-1-vinyl)styryl]-6-(*tert*-butyl)-4*H*-pyran-4-ylidene}-propanedinitrile (16):

To a solution of compound **13** (35 mg, 0.031 mmol, 1 eq.), compound **Fuc(EG)$_3$N$_3$** (97 mg, 0.215 mmol, 7 eq.) and copper iodide (CuI, 4 mg, 0.019 mmol, 0.6 eq.) in dry DMF (4 mL) was added N,N-diisopropylethylamine (28 mg , 0.215 mmol, 7 eq., 0.036 mL). The resulting mixture was stirred under microwaves activation at 110°C during 60 min. Then the mixture was diluted with EtOAc (50 mL), washed with saturated EDTA aqueous solution (50 mL x 3). The combined aqueous fractions were extracted with EtOAc (30 mL). The organic layer was combined, dried ($MgSO_4$), concentrated and purified with silica gel column chromatography (CH_2Cl:MeOH = 30:1) to obtain compound **16** (101 mg, 86%) as red amorphous solid.

R_f = 0.20 (CH_2Cl:MeOH = 30:1)

^{1}H NMR (300 MHz, $CDCl_3$): 1.04 (d, 18H, *J* = 6.6 Hz, H-6), 1.29 (s, 9H, C(C<u>H</u>$_3$)$_3$),1.90, 1.97, 2.08 (3s, 3×18H, CH_3CO), 3.50-3.59 (m, 36H, OCH_2CH_2O, Fuc-OCH_2), 3.66-3.73 (m, 6H, Fuc-OCH_2), 3.74 (s, 12H, (O-C<u>H</u>$_2$)$_3$-C), 3.81 (d, 12H, *J* = 5.1 Hz, OC<u>H</u>$_2$CH$_2$N), 4.14 (q, 6H, *J* = 6.6 Hz, H-5), 4.21 (s, 4H, OC<u>H</u>$_2$CO), 4.45 (t, 12H, *J* = 5.4 Hz, OCH$_2$C<u>H</u>$_2$N), 4.51 (bs, 12H, OCH$_2$-triaz), 4.99-5.05 (m, 12H, H-1, H-2), 5.19-5.21 (m, 6H, H-4), 5.24-5.30 (m, 6H, H-3), 6.47 (d, 2H, *J* = 2.1 Hz, H-3pyran), 6.54-6.69 (m, 6H, H-5pyran, <u>H</u>C=CH-Ph, 2×H-Ar), 6.84-6.98 (m, 10H, 2×NH, 8×H-Ar), 7.04-7.06 (m, 3H, H-Ar), 7.23-7.28 (m, 3H, 2×H-Ar, HC=C<u>H</u>-Ph), 7.63 (s, 6H, H-triaz).

^{13}C NMR (75 MHz, $CDCl_3$): 15.8 (C-6), 20.6, 20.63, 20.7 (3×CO<u>C</u>H$_3$), 28.0 (C(<u>C</u>H$_3$)$_3$), 36.9 (<u>C</u>(CH$_3$)$_3$), 50.0 (OCH$_2$<u>C</u>H$_2$N), 53.5 ((O-CH$_2$)$_3$-<u>C</u>), 59.6(<u>C</u>(CN)$_2$), 64.2 (C-5), 64.61 and 64.67 (OCH$_2$-triaz), 67.1 (O<u>C</u>H$_2$CO), 67.3 (Fuc-OCH$_2$), 67.9 (C-3), 68.1 (C-2), 68.6 (O-C<u>H</u>$_2$)$_3$-C), 69.4 (O<u>C</u>H$_2$CH$_2$N), 70.1 (OCH$_2$), 70.44 (OCH$_2$), 70.48(OCH$_2$), 71.1 (C-4), 96.1 (C-1), 102.5 (C-3pyran), 107.0 (C-5pyran), 113.9 (CH-Ar), 114.1 (CH-Ar), 115.1 (2×CN), 118.0 (H<u>C</u>=CH-Ph), 123.62 and 123.66 (CH^{triaz}), 126.5 (CH-Ar), 127.3 (CH-Ar), 127.9 (CH-Ar), 131.3 (CH-Ar), 132.0 (CH-Ar), 132.3 (<u>C</u>^{Ar}-CH=CH-Pyran), 132.6 (CH-Ar), 132.61 (CH-Ar), 136.91 (C^{Ar}-C=C), 136.93 (C^{Ar}-C=C), 137.4 (HC=C<u>H</u>-Ph), 139.1 (<u>C</u>^{Ar}-C=C), 140.7 (<u>C</u>^{Ar}-C=C), 143.3 (C=C), 144.3 and 144.4 (C^{triaz}), 146.5 (C=C), 155.8 (C-6pyran), 155.9 (<u>C</u>^{Ar}-O), 156.6 (<u>C</u>^{Ar}-O), 158.9 (C-2pyran), 167.4 (NH<u>C</u>OCH$_2$) 169.9, 170.3, 170.5 (3×<u>C</u>OCH$_3$), 172.1 (C-4pyran).

HR-ESI-MS *m*/*z*: calcd. for $C_{178}H_{244}N_{22}O_{71}$ $[M+4H]^{4+}$ 956.4034, found 956.4035.

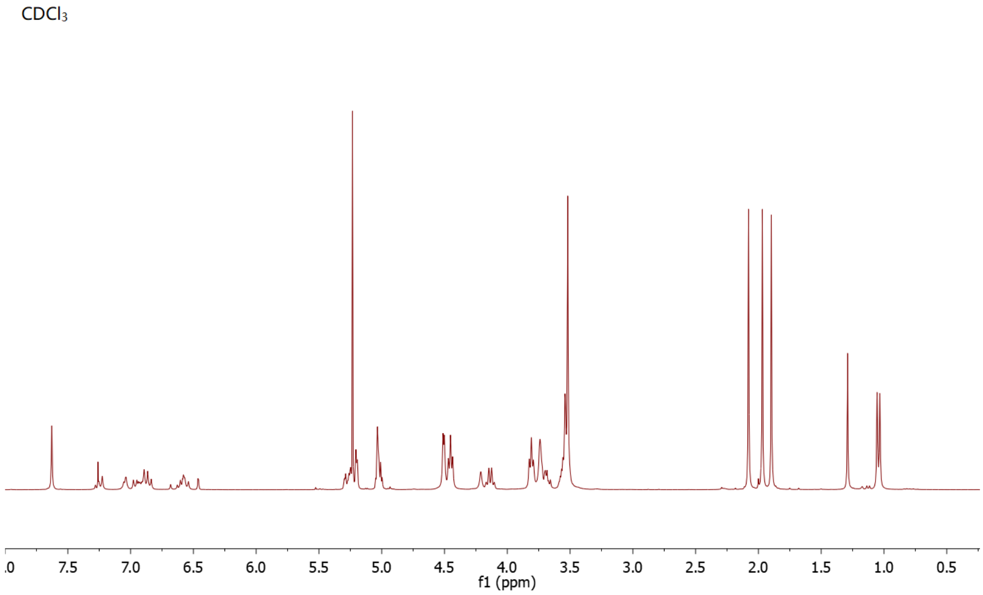
CDCl3
.0 7.5 7.0 6.5 6.0 5.5 5.0 4.5 4.0 3.5 3.0 2.5 2.0 1.5 1.0 0.5
f1 (ppm)

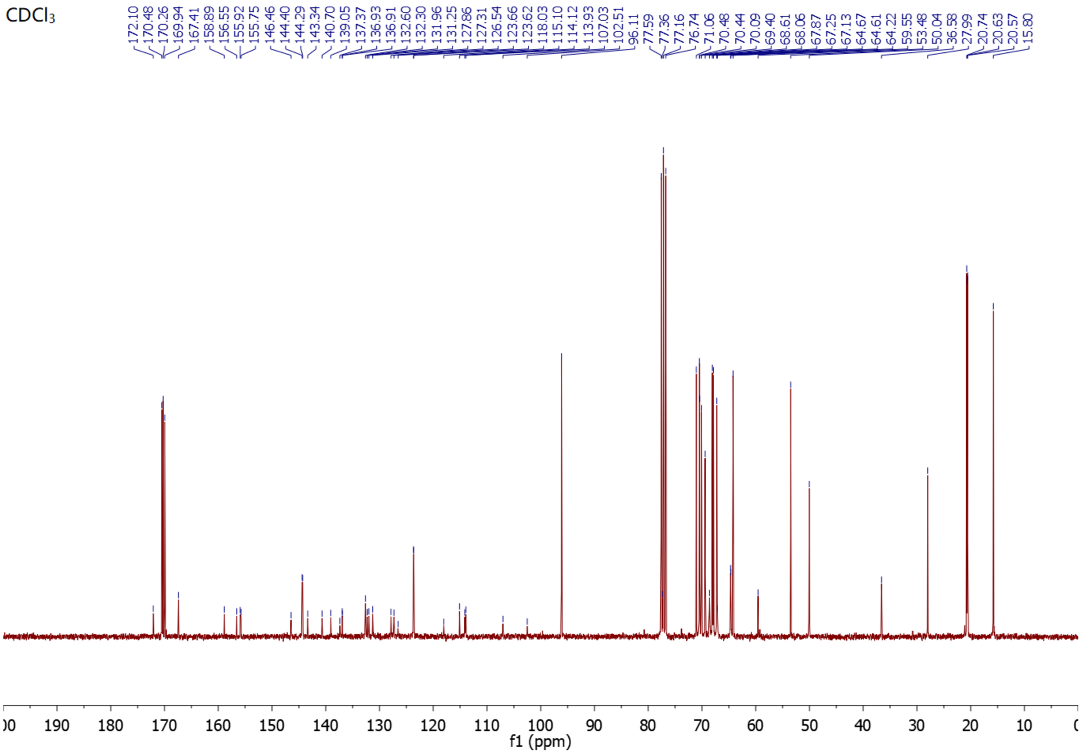
CDCl3
172.10 170.48 170.26 169.94 167.41 158.89 156.55 155.92 155.75 146.46 144.40 144.29 143.34 140.70 139.05 137.37 136.93 136.91 132.60 132.30 131.96 131.25 127.86 127.31 126.54 123.66 123.62 118.03 115.10 114.12 113.93 107.03 102.51 96.11 77.59 77.36 77.16 76.74 71.06 70.48 70.44 70.09 69.40 68.61 68.06 67.87 67.25 67.13 64.67 64.61 64.22 59.55 53.48 50.04 36.58 27.99 20.74 20.63 20.57 15.80
00 190 180 170 160 150 140 130 120 110 100 90 80 70 60 50 40 30 20 10 0
f1 (ppm)

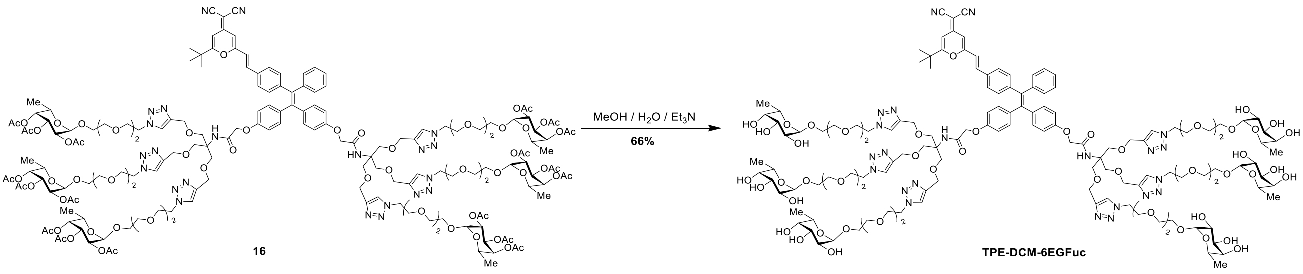

(E)-2-{2-[4-(2,2-di-{4-[N-tris({1-[1-(α-L-fucopyranosyloxy)-3,6-dioxaoct-8-yl]-1,2,3-triazol-4-yl-methoxy}methyl)methamido-eth-2-yl]oxy}phenyl-1-phenyl-1-vinyl)styryl]-6-(*tert*-butyl)-4H-pyran-4-ylidene}-propanedinitrile (TD-Fuc$_6$) :

To a solution of compound **16** (101 mg, 0.026 mmol, 1 eq.) in mixed solvent with MeOH (8 mL) and H_2O (2 mL) was added Et_3N (2 mL). The resulting mixture was stirred under argon at RT during 20 h. The solution was evaporated Et_3N after starting materials disappeared by TLC tracing. The crude product was dissolved with H_2O (2 mL) and purified with C^{18} reverse phase column (H_2O:MeOH = 1:0 to 0:1) to afford compound **TD-Fuc$_6$** (52 mg, 66%) as a red amorphous solid.

^{1}H NMR (300 MHz, D_2O/CD_3OD): 1.40 (d, 9H, J = 1.8 Hz, C-6), 1.42 (d, 9H, J = 1.8 Hz, C-6), 1.61 (s, 9H, $C(C\underline{H}_3)_3$), 3.79-3.87 (m, 44H, Fuc-OCH_2, OCH_2), 3.92-3.93 (m, 6H, C-4), 3.95-4.03 (m, 30H, C-2, C-3, Fuc-OCH_2, $(O\text{-}C\underline{H}_2)_3$-C), 4.13 (m, 12H, $OC\underline{H}_2CH_2N$), 4.18-4.24 (m, 6H, H-5), , 4.61 (s, 4H, $OC\underline{H}_2CO$), 4.78-4.79 (m, 24H, $OCH_2C\underline{H}_2N$, OCH_2-triaz), 5.04 (t, 6H, J = 2.4 Hz, H-1), 6.78 (d, 1H, J = 1.5 Hz, H-3pyran), 6.90-7.00 (m, 6H, H-5pyran, $\underline{H}$C=CH-Ph, 2×H-Ar), 7.13-7.26 (m, 10H, 2×NH, 8×H-Ar), 7.32-7.35 (m, 3H, H-Ar), 7.60-7.66 (m, 3H, 2×H-Ar, HC=C$\underline{H}$-Ph), 8.20, 8.22 (bs, 6H, H-triaz).

^{13}C NMR (75 MHz, D_2O/CD_3OD): 16.7 (C-6), 28.3 ($C(\underline{C}H_3)_3$), 37.7 ($\underline{C}(CH_3)_3$), 51.4 ($OCH_2\underline{C}H_2N$), 58.4 ($\underline{C}(CN)_2$), 61.1 ($(O\text{-}CH_2)_3\text{-}\underline{C}$), 65.2 ($O\underline{C}H_2$-triaz), 67.6 (C-5), 68.2 (Fuc-OCH_2), 68.4 ($O\underline{C}H_2CO$), 68.4 ($(O\text{-}\underline{C}H_2)_3$-C), 69.9 (C-2), 70.2 ($O\underline{C}H_2CH_2N$), 71.17 ($OCH_2$), 71.19 ($OCH_2$), 71.2 ($OCH_2$), 71.5 (C-3), 73.4 (C-4), 100.4 (C-1), 103.4 (C-3pyran), 108.0 (C-5pyran), 115.32 (CH-Ar), 115.5 (CH-Ar), 116.4 (2×CN), 119.4 (H$\underline{C}$=CH-Ph), 125.9 (CH^{triaz}), 127.7 (CH-Ar), 128.7 (CH-Ar), 129.0 (CH-Ar), 132.2 (CH-Ar), 133.0 (CH-Ar), 133.56 (CH-Ar), 133.62 (CH-Ar), 134.1 ($\underline{C}^{Ar}$-CH=CH-Pyran), 138.36 (C^{Ar}-C=C), 138.42 (C^{Ar}-C=C), 138.9 (HC=C$\underline{H}$-Ph), 141.8 ($\underline{C}^{Ar}$-C=C), 142.1 ($\underline{C}^{Ar}$-C=C),144.6 (C=C), 145.5 (C^{triaz}), 147.6 (C=C), 157.5 ($\underline{C}^{Ar}$-O), 157.7 ($\underline{C}^{Ar}$-O), 158.7 (C-6pyran), 161.2 (C-2pyran), 170.4 ($NH\underline{C}OCH_2$), 174.7 (C-4pyran).

HR-ESI-MS m/z: calcd. for $C_{142}H_{207}N_{22}O_{53}$ $[M+3H]^{3+}$ 1022.8054, found 1022.8077.

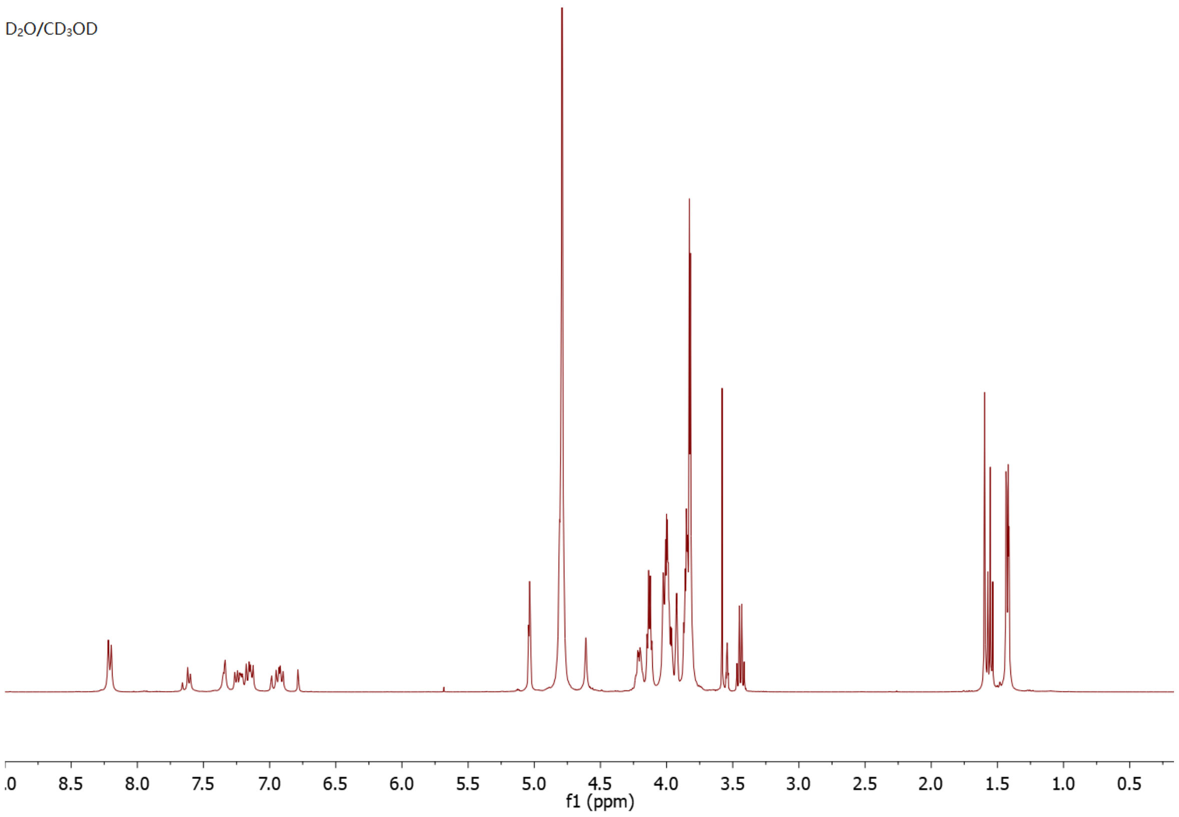
D2O/CD3OD
9.0 8.5 8.0 7.5 7.0 6.5 6.0 5.5 5.0 4.5 4.0 3.5 3.0 2.5 2.0 1.5 1.0 0.5
f1 (ppm)

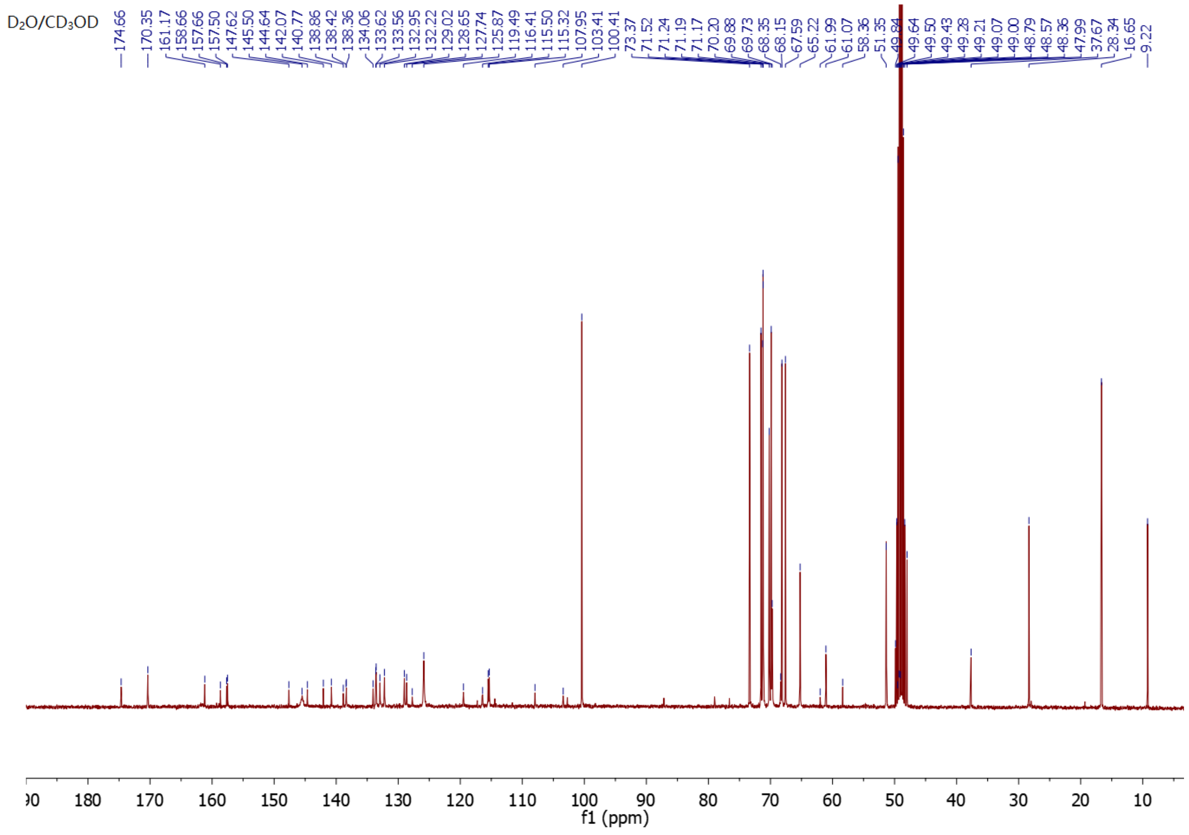
D2O/CD3OD
174.66 170.35 161.17 158.66 157.66 157.50 147.62 145.50 144.64 142.07 140.77 138.86 138.42 138.36 134.06 133.62 133.56 132.95 132.22 129.02 128.65 127.74 125.87 119.49 116.41 115.50 115.32 107.95 103.41 100.41
73.37 71.52 71.24 71.19 71.17 70.20 69.88 69.73 68.35 68.15 67.59 65.22 61.99 61.07 58.36 51.35 49.84 49.64 49.50 49.43 49.28 49.21 49.07 49.00 48.79 48.57 48.36 47.99 37.67 28.34 16.65 9.22
190 180 170 160 150 140 130 120 110 100 90 80 70 60 50 40 30 20 10
f1 (ppm)

Photophysical and biological measurements

UV-vis absorption. The UV-vis absorption spectra were measured at room temperature using a Varian Cary 60 UV-vis spectrophotometer. All spectra were corrected for background intensities by subtracting the spectra of pure solvent measured under identical conditions. UV-vis-NIR absorption spectra ranging 0-800 nm were measured.

Fluorescence spectroscopy. The fluorescence measurements were carried out at room temperature using an Agilent Cary Eclipse fluorescence spectrophotometer (slit width 5-5 nm, 650V) in distilled water or in PBS (0.01 M, pH 7.4). All fluorescence-based experiments were repeated three times with representative data shown.

Calculation of fluorescence quantum yields. TD-Gal$_6$ (with a final concentration of 10 μM) was dissolved in PBS (0.01 M, pH 4.0 or 7.4). Then, β-Gal (with a final concentration of 10 U mL^{-1}) was added, and the resulting mixture was incubated at 37 °C for 50 min. A PBS (0.01 M, pH 7.4) solution of Rhodamine B (with a final concentration of 5 μM) was also prepared. The UV-vis absorbance was measured at room temperature on a Varian Cary 60 UV-vis spectrophotometer. The UV absorption intensity at 420 nm was collected. The fluorescence measurements were carried out at room temperature on an Agilent Cary Eclipse fluorescence spectrophotometer with an excitation wavelength of 420 nm. Integrated fluorescence curves between 450 nm and 850 nm were measured. Finally, the fluorescence quantum yield was calculated according to equation (1).

$$Y_u = Y_r \cdot \frac{F_u}{F_r} \cdot \frac{A_r}{A_u} \cdot \frac{n_u^2}{n_r^2} \qquad (1)$$

where Y_u = fluorescence quantum yield of the probe, Y_r = fluorescence quantum yield of rhodamine B, F_u = integrated fluorescence intensity of the probe, F_r = integrated fluorescence intensity of rhodamine B, A_u = absorbance of the probe, A_r = absorbance of rhodamine B, and n = refractive index.

Dynamic light scattering. Dynamic light scattering was carried out on a Horiba LB-550 Dynamic Light Scattering Nano-Analyzer.

Cell culture. SKOV-3 cells were cultured at 37 °C under a humidified 5% CO_2 atmosphere in McCoy's 5A (Gibco, Gland Island, NY, USA), which were supplemented with 10% fetal bovine serum (Gibco, Gland Island, NY, USA) and 1% penicillin-streptomycin. HUVEC and 293T cells were maintained in a Dulbecco's Modified Eagle's Medium (Invitrogen, Carlsbad, CA, USA) supplemented with 10% fetal bovine serum (Gibco, Gland Island, NY, USA) in a humidified atmosphere of 5% CO_2 and 95% air at 37 °C. Wi-38 cells (human embryonic lung diploid fibroblasts) were cultured in 5% CO_2 atmosphere under 37°C in Eagle's Minimum Essential Medium (Sigma, USA) supplemented with 10% fetal bovine serum (Gibco, Gland Island, NY, USA).

Oxidative stress-induced cell senescence by treatment with exogenous H_2O_2. Exponentially-growing WI-38 cells at the 6th passage were plated into 6 cm cell culture dish (Corning, USA) and treated with H_2O_2 (150 μM, Sinopharm Chemical Reagent Co., Ltd, Shanghai, China) for 2h, followed by 48 h continuous culture.

Confocal laser-scanning microscopy for the fluorescence imaging o β-Gal and AFU in cells. Cells cultured in growth medium supplemented with 10% FBS were added to a 24-well microplate. Cells were maintained in

a humidified atmosphere of 5% CO_2 and 95% air at 37 °C overnight. For β-Gal imaging, the cells were incubated with **TD-Gal$_6$** (20 μM) for 30 min. For the colocalization experiments, the cells were incubated with **TD-Gal$_6$** (20 μM) for 30 min, followed by the treatment of an organelle-specific tracker (Mito-Tracker or Lyso-Tracker, 100 nM) for 30 min. For long-term fluorescence-based cell tracking, the cells were incubated with **TD-Gal$_6$** (20 μM) at different time points. For AFU imaging, the cells were pretreated with an AFU inhibitor (1-deoxy-fuconojirimycin) for 3 h, and then incubated with **TD-Fuc$_6$** (20 μM) for 30 min. Then, cells on the microplate were rinsed using warm PBS and fixed by 4% paraformaldehyde for 20 min at room temperature. The cell nuclei were stained with Hoechst 33342 (5 μg mL^{-1}) for 5 min. Immediately after sealing, the fluorescence was detected and photographed with a confocal laser-scanning microscope (Olympus, Japan, Hoechst 33342 excitation: 405 nm, emission: 440-480 nm; **TD-Gal$_6$** and **TD-Fuc$_6$** excitation: 488 nm, emission: 580-620 nm; Mito-Tracker or Lyso-Tracker dye excitation: 559 nm, emission: 570-590 nm). Statistical analysis was performed using Student's unpaired t-test. All experiments were repeated three times with representative data shown.

Cell viability and phototoxicity assay. Cells were plated on 96-well plates in growth medium overnight and then treated with different concentrations of **TD-Gal$_6$**. After 24 h incubation, the 3-(4,5-dimethylthiazol-2-yl)-5-(3-carboxymethoxyphenyl)-2-(4-sulfophenyl)-2H-tetrazolium (MTS)/phenazine methosulfate (PMS) solution was added. After 2 h, the absorbance of each well was measured at 490 nm using a M5 microplate reader (Molecular Device, USA). The optical density from the MTS assay was directly proportional to the number of viable cells. All experiments were repeated three times with representative data shown.
